# *In silico* characterization of hypothetical proteins from *Orientia tsutsugamushi* str. Karp uncovers virulence genes

**DOI:** 10.1016/j.heliyon.2019.e02734

**Published:** 2019-11-01

**Authors:** Nikhat Imam, Aftab Alam, Rafat Ali, Mohd Faizan Siddiqui, Sher Ali, Md. Zubbair Malik, Romana Ishrat

**Affiliations:** a Institute of Computer Science and Information Technology, Magadh University, Bodhgaya, India; b Centre for Interdisciplinary Research in Basic Science, Jamia Millia Islamia, New Delhi, India; c International Medical Faculty, Osh State University, Osh City, 723500, Kyrgyz Republic (Kyrgyzstan); d School of Computational and Integrative Sciences, Jawaharlal Nehru University, New Delhi, Delhi, 110067, India

**Keywords:** Biochemistry, Bioinformatics, Genetics, Microbiology, Functional annotation, Hypothetical proteins (HPs), Scrub typhus, ROC analysis, Virulence

## Abstract

Scrub typhus also known as bush typhus is a disease with symptoms similar to Chikungunya infection. It is caused by a gram-negative bacterium *Orientia tsutsugamushi* which resides in its vertebrate host, Mites. The genome of *Orientia tsutsugamushi* str. Karp encodes for 1,563 proteins, of which 344 are characterized as hypothetical ones. In the present study, we tried to identify the probable functions of these 344 hypothetical proteins (HPs). All the characterized hypothetical proteins (HPs) belong to the various protein classes like enzymes, transporters, binding proteins, metabolic process and catalytic activity and kinase activity. These hypothetical proteins (HPs) were further analyzed for virulence factors with 62 proteins identified as the most virulent proteins among these hypothetical proteins (HPs). In addition, we studied the protein sequence similarity network for visualizing functional trends across protein superfamilies from the context of sequence similarity and it shows great potential for generating testable hypotheses about protein structure-function relationships. Furthermore, we calculated toplogical properties of the network and found them to obey network power law distributions showing a fractal nature. We also identifed two highly interconnected modules in the main network which contained five hub proteins (KJV55465, KJV56211, KJV57212, KJV57203 and KJV57216) having 1.0 clustering coefficient. The structural modeling (2D and 3D structure) of these five hub proteins was carried out and the catalytic site essential for its functioning was analyzed. The outcome of the present study may facilitate a better understanding of the mechanism of virulence, pathogenesis, adaptability to host and up-to-date annotations will make unknown genes easy to identify and target for experimentation. The information on the functional attributes and virulence characteristic of these hypothetical proteins (HPs) are envisaged to facilitate effective development of novel antibacterial drug targets of *Orientia tsutsugamushi*.

## 1 Introduction

*Orientia tsutsugamushi*, an obligate intracellular bacterium, is a causative agent of scrub typhus or *tsutsugamushi* disease. The clinical manifestation of Scrub typhus is diverse, ranging from a nonspecific febrile illness to severe multiorgan dysfunction [1, 2]. There is an estimated one million new scrub typhus cases each year, and over one billion individuals around the world are at risk. Without appropriate treatment, the case fatality rate of scrub typhus can reach up to 30% or even higher [3]. Scrub typhus is an endemic disease to a sect of the world known as ‘the *tsutsugamushi* triangle’, which extends from Northern Japan and far East Russia in the North, to Northern Australia in the South and to Pakistan in the West [4]. India is an integral component of this triangle and it has been shown that during 2007–2017, in India, while dengue and Chikungunya are targeted as a focused disease meanwhile, scrub typhus affected a large number of the population of northern India (Himachal Pradesh) and other states including Rajasthan, Jammu and Kashmir, Uttrakhand, Puducherry, Sikkim, Bihar, West Bengal, Meghalaya, Rajasthan, Maharashtra, Karnataka, Andhra Pradesh and Tamil Nadu [5].

It is an occupational disease normally found amongst farmers working in the fields [4]. The possibilities of *tsutsugamushi* disease are higher in fruit farmers and chestnut gatherers. Similarly, the prevalence of infection is more likely to occur in tropical areas, especially agricultural lands, people living close to bushes and wood piles, farmers, rodent observers and those rearing domestic animals [6]. It mostly occures in the rainy season, However, epidemic periods have been reported during the winter season in southern India [7]. Certain areas such as forest clearings, river banks, bushy areas and grassy lands provide optimal conditions for the infected mites to survive. Thus, the best measure would be to avoid going to such places. The symptoms of *tsutsugamushi* disease are very similar to that of other arboviral infections like Dengue, Chikungunya and West Nile causing high grade fever (>104 °F) of 7–14 days duration, having symptoms like severe headache, anorexia, myalgia, maculopapular rash, profuse sweating and swelling of major lymph nodes (neck region and groin). The genome sequence of *O. tsutsugamushi* str. Karp is available in the NCBI database (Taxonomy ID: 1359185, GenBank Accession #: NZ_LYMA00000000.2) containing 1563 genes encoding proteins. The proteins with unknown functions are referred to as hypothetical proteins (HPs). The HPs are predicted to be expressed from an open reading frame (ORF), but have no experimental evidence of translation. These proteins constitute a substantial fraction in both prokaryotes and eukaryotes, including humans [8]. Annotation of HPs assist in finding newer structures and functions enabling their classification into other pathways and cascades. They also aid as markers and pharmacological targets for drug design, discovery, and in screening [9].

Amongst prokaryotes, the proteins from a number of bacteria remain uncharacterized despite the fact that their genome sequences are known. This provides an opportunity to annotate these putative proteins with respect to their functions. Many “putative proteins” are shared by a number of bacterial species, which suggest their much broader biological roles within and across species. Proteins that occur in various species are represented as orthologous groups that are useful for functional analyses and annotations of the newly sequenced genomes [10]. The ability to predict the function of a gene based on its sequence is an important area of biological research.

The main objective of our study is to annotate the putative functions, determine its classification and identify the most virulent proteins of all 344 HPs. So we have analysed the sequences of all 344 HPs from *O. tsutsugamushi* str. Karp. The systematic analysis began with the prediction of physiochemical properties, sub-cellular localizations, domain/motif predictions, and function annotation using established bioinformatics databases and tools. The ROC (Receiver operating characteristic) analysis was used to assess the performance of approaches used (integrated bioinformatics tools) in the predictions on the basis of confidence and precision levels. If the confidence level is high for more than three tools, this indicates the same functions. So, we have successfully annotated functions of all 344 HPs of *O. tsutsugamushi* str. Karp. All 344 HPs were classified according to their functional properties like binding proteins, transporter proteins, kinase, hydrolae, transferase, etc. We analysed most virulent proteins for their involvement in defining different cellular fates. We believe that such analysis expands our understanding regarding, HPs and their functional role in the cell and provides an opportunity to discover novel potential drug targets.

## 2 Methods

### 2.1 Sequence retrieval

We analysed the genome of *O. tsutsugamushi* str. Karp and found 1,563 protein coding genes (http://www.ncbi.nlm.nih.gov/genome) and a total of 344 proteins as hypothetical proteins (HPs). The sequences of all 344 HPs proteins were retrieved from Uniprot (http://www.uniprot.org) in the FASTA format. The sequence similarity search was performed via pBLAST against the non-redundant database. The similarity search against the Protein Data Bank (PDB) yield no potential structural templates. Generally, HPs contain low identity as compared to other known or annotated proteins.

### 2.2 Physicochemical properties of HPs

The physicochemical parameters of all 344 HPs were studied using Expasy's ProtParam server [11] (www.web.expasy.org/protparam), which was then used for theoretical measurements such as molecular weight, isoelectric point, extinction coefficient [12], instability index [13], aliphatic index and grand average of hydropathicity (GRAVY) [14]. The extinction coefficient is the measure of the amount of light that proteins absorb at a certain wavelength. An estimation of the stability of a protein in a test tube is provided by the instability index. The aliphatic index of a protein is defined as the relative volume occupied by aliphatic side chain amino acids. The GRAVY score for a peptide or protein is calculated as the sum of the hydropathy values of all of the amino acids, divided by the number of residues in the query sequence. The predicted properties of HPs are listed in S1_ Table.

### 2.3 Sub-cellular localization & protein classification

The functions of proteins are usually related to its sub-cellular localization. Thus, the ability to predict sub-cellular localization directly from protein sequences will be useful for inferring its functions at the cellular level. It is a well known fact that a protein present in the cytoplasm can act as a possible drug target, while membrane proteins found on the surface are considered as vaccine targets [15]. The sub-cellular localization of HPs were predicted using PSLpred (it is a hybrid approach-based method that integrates PSI-BLAST and three SVM (Support vector machine) modules based on compositions of residues, dipeptides and physico-chemical properties [16] and CELLO (multi-class SVM classification system) (S2_ Table) [17].

### 2.4 Functional domain/motif prediction

The aim of the functional domain prediction is to find out the conserved part of a protein because domains often form functional units. The functional domains of HPs were predicted by using various publicly available databases such as ScanProsite [18], SMART [19], Motif Scan (including peroxiBase profiles, HAMAP, PROSITE patterns, Pfam HMMs for local & global models) [20] and PFP-FunDSeqE [21]. ScanProsite provides a web interface to identify protein matches against signatures from the PROSITE database. The Simple Modular Architecture Research Tool (SMART) [19] was used for similarity search based on domain architecture and profiles rather than by direct sequence similarity. PFP-FunDSeqE server [22] covers the protein fold types and compared with the existing predictors tested by the same stringent benchmark dataset, the new predictor can, for the first time, achieve high success rate. The detailed results are given in (S3_ Table).

### 2.5 Virulence protein prediction

The identification of virulent proteins in bacterial protein sequences is useful in estimating its pathogenic ability and understanding the complex virulence mechanism of pathogenesis [23]. Here, we used VirulentPred tool (Bi-layer cascade Support Vector Machine) (http://bioinfo.icgeb.res.in/virulent/) for the identification of virulence factors among HPs. It is a Support Vector Machine (SVM) based method to predict virulence proteins with accuracy. We considered five modules based on protein features such as Amino Acid Compositions, Dipeptide Composition, PSI-BLAST created PSSM Profiles, Higher Order Dipeptide Composition Based and Cascade of SVMs and PSI-BLAST. These modules gave SVM predicted scores and similarity-search based information for each of the 344 sequences (S4_ Table). We selected the average of highly significant values from each module i. e; average values >1.0.

### 2.6 Function prediction

The accurate annotation of protein function is a key to understand the processes of life at the molecular level. In our study, we have predicted the gene ontology (Moleculer function and Biological process) of most virulent proteins. We used **PFP** (Protein function prediction) and **Argot2** (Annotation Retrieval of Genel Ontology Terms), which quickly process thousands of sequences for functional inference.

### 2.7 Performance assessment

The predicted functions of 344 HPs from the *O. tsutsugamushi* str*.* Karp were validated using the Receiver Operating Characteristic (ROC) analysis. We predicted the function of 60 proteins (functions already known) to check the accuracy of a tools which were used to annotate our 344 HPs. The details are given in (S5_Table). The diagnostic efficacy was evaluated at twelve levels. The two binary numerals ‘‘0’’ or ‘‘1’’ were used to classify the prediction as true positive (‘‘1’’) or true negative (‘‘0’’).

### 2.8 Sequence similarity networks (SSN)

The dramatic increase in heterogeneous types of biological data—in particular, the abundance of new protein sequences—requires fast and user-friendly methods for organizing this information in a way that enables functional inference. The most widely used strategy to link sequence or structure to function, homology-based function prediction, relies on the fundamental assumption that sequence or structural similarity implies functional similarity. We also calculated topological properties of the parent network, the topological analysis helps to understand the structure of a network which facilitates in understanding the hidden mechanisms. The networks properties (*Degree distribution, Neighborhood connectivity, Clustering co-efficient, Betweenness centrality and Closeness centrality*) were analysed to seek the important behaviours of the network. In our study we have taken all the top 62 virulent proteins to construct the sequence similarity networks (SSN) using STRING database (V-10.0) [24] and find clusters (highly interconnected regions) in the network using MCODE(1.5.1) in Cytoscape [25].

### 2.9 Secondary and tertiary structure prediction of proteins

The sequences of five hub proteins, namely KJV55465 (OTBS_1583), KJV56211 (OTBS_0920), KJV57212 (OTBS_0674), KJV57203 (OTBS_0675) and KJV57216 (OTBS_0676) were considered for predicting secondary and tertiary structures. To obtain the probable 2D structure for hub proteins, we used PSIPRED server [26], which is a simple and accurate 2D structure prediction server, incorporating two feed-forward neural networks which perform an analysis on output obtained from PSI-BLAST [27].

The 3D prediction was made with through homology modelling using the **MODELLER** (version 9.21) [28] and best model structure was choosen based on DOPE score and GA341 assessment score, This methodology is very accurate for modelling 3D protein structures according to the sequence identity between the target sequence and the template proteins. Thus, the final predicted model was validated using the PROCHECK programs [29]. It analyzes the stereochemical quality of the protein model by analyzing residue-by-residue geometry and overall structure geometry.

## 3 Results

**Fig. 1:**
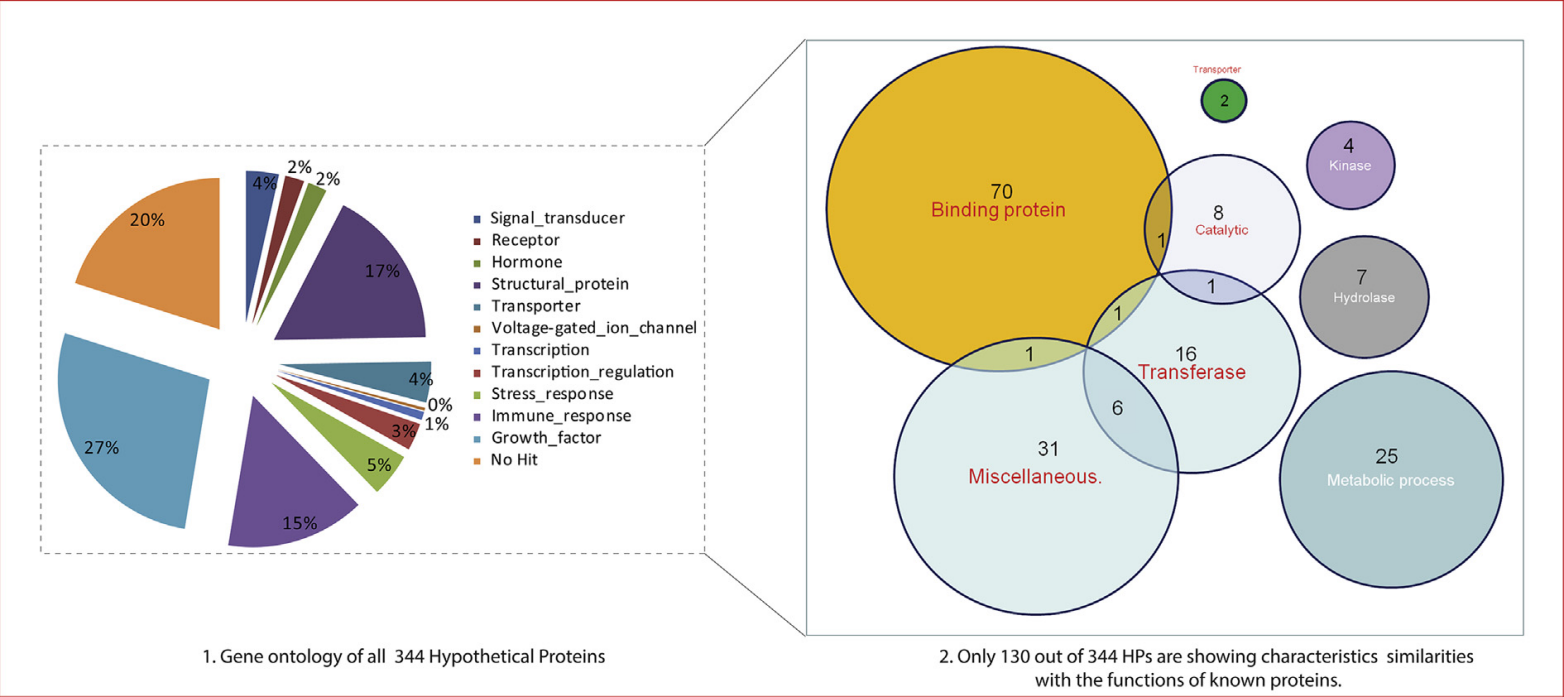
(1) The gene ontology of all 344 hypothetical proteins. (2) Only 130 out 0f 344 HPs showed characteristic similarities with the functions of known proteins including binding proteins, transporter proteins, enzymes, metabolic pathways and other miscellaneous classes.

Our study analysed the sequence from *O. tsutsugamushi* str. Karp genome using advanced bioinformatics tools. After retrieval of all the 344 HPs sequences, we calculated the physiochemical properties and found that most of the HPs are localized in the cytoplasmic region of the cell. Generally, the cytoplasmic and inner membrane proteins are considered as potential drug targets but extracellular and outer membrane proteins are effective for vaccine [30]. Finally, We have classified all the 344 HPs into different protein classes like Signal_transducer (12 HPs), Receptor (07 HPs), Hormone (07 HPs), Structural_protein (59 HPs), Transporter (15 HPs), Voltage-gated_ion_channel (01 HP), Transcription (03 HPs), Transcription_regulation (10 HPs), Stress_response (16 HPs), Immune_response (51 HPs), Growth_factor (94 HPs). There was no evidence to classify the remaining 69 HPs into protein classes. Furthermore, we found that 130 out of 344 HPs are showed similar characteristics with the functions of known proteins. All the characterized hypothetical proteins (HPs) belong to various protein classes like enzymes, transporters, binding proteins, metabolic process and catalytic activity, kinase activity etc. The details of HPs are given in Fig. 1.

### 3.1 Protein classification

#### 3.1.1 Binding proteins

We characterized a total of 70proteins that showed properties like DNA binding (47 HPs), nucleotide binding (02 HPs), metal binding (02 HPs), protein binding (08 HPs), ATP- binding (04 HPs), Zinc ion binding (02 HPs), Cation binding (01 HP), calcium ion binding (02 HPs) and RanGTPase binding (01 HP). The DNA binding protein class was found to be a major class which contained 47 hypothetical proteins (HPs). It is well known fact that bacterial DNA binding protein plays a crucial role in DNA replication; the protein is involved in stabilizing the lagging strand as well as interacting with DNA polymerase III [31]. Currently, many more functions of bacterial DNA binding proteins have been identified, including the regulation of gene expression by histone-like nucleoid-structuring protein [32].

#### 3.1.2 Transporter

Two HPs (KJV56037 and KJV55034) are annotated to be involved in protein and hydrogen transporation respectively. Bacterial transport proteins mediate passive and active transport of small solutes across membranes. Protein involved in the transport of hydrogen ions across a membrane, used to power processes such as ATP synthesis in the bacteria. The transporter systems of *O. tsutsugamushi* comprise of secondary transporters, in which transport activity and ABC-type transporters are driven by an ion gradient across the membrane and ATP hydrolysis respectively [33].

#### 3.1.3 Kinase activity

We identified four ​proteins that have kinase activity including KJV54167, KJV54168, KJV56574 and KJV51784. It has been shown that bacteria have a versatile repertoir of protein kinases like histidine and aspartic acid kinases, serine/threonine kinases, and more recently tyrosine and arginine kinases. Currently, Tyrosine phosphorylation is known to be a key regulatory device of bacterial physiology, linked to exopolysaccharide production, virulence, stress response and DNA metabolism [34].

#### 3.1.4 Hydrolase activity

Seven hypothetical proteins showed hydrolase activity that catalyze the hydrolysis of a chemical bond. Hydrolytic enzymes play key roles in the invasion of the host tissue and evading the host defense mechanism [35]. The genomes of gram-negative and gram-positive bacterial species all encode an inclusive variability of hydrolase enzymes that accounts for the specific cleavage of different peptidoglycan (PG) bonds; hydrolases are involved in several critical functions, including peptidoglycan (PG) maturation, turnover, recycling, autolysis, and cleavage of the septum during cell division [36].

#### 3.1.5 Transferase activity

A total of 16hypothetical proteins (HPs) were found to exhibit transferase activity. A transferase is a class of enzyme that performs the transfer of specific functional groups (e.g. a methyl or glycosyl group) from one molecule to another. Bacterial interactions with the host mostly depend on the bacterial glycome. Particularly the bacterial glycome is largely determined by glycosyltransferases (GTs) [37].

#### 3.1.6 Metabolic process and catalytic activity

A total of ​8 HPs were found which were involved in both biosynthetic and catabolic metabolic processes. *O. tsutsugamushi* has a large genome size, and its metabolic pathways have been well organised due to the existence of repeated sequences. Overall, *O. tsutsugamushi* and Rickettsia have similar metabolic pathways but they are slightly different in many pathways like in the TCA-cycle, carbohydrate metabolism, bacterial cell wall synthesis and in transportations. The *O. tsutsugamushi* has five copies of ATP/ADP translocase. Recently, it has been shown that the translocases have differential transportation properties for nucleotides. There are several components of salvage pathways of purine and pyrimidine biosynthesis present in *O. tsutsugamushi*. The absence of enzymes for the interconversion of adenine and guanine suggests that these bacteria depend on the host for both purines and may import them via different subtypes of ATP/ADP translocases [33]. In contrast to *O. tsutsugamushi*, the members of anaplasmataceae is well equipped with the enzymes for the de novo nucleotide synthetic pathways and the pentose phosphate pathway. The majority of genes for fatty acid biosynthesis were present in *O. tsutsugamushi* but the β-oxidation system of fatty acids for energy generation was absent in *O. tsutsugamushi*.

#### 3.1.7 Miscellaneous functions

We have found 31proteins exhibiting miscellaneous functions, such as peptide activity, oxidoreductase activity, NADH dehydrogenase (quinone) activity, Thiosulfate sulphur transference activity, RNA directed DNA polymerase, Structural molecule activity, RNA polymerase II transcription cofactor activity etc. Identification of bacterial virulent protein sequences has implications for characterization of novel virulence-associated factors, finding novel drug/vaccine targets against proteins indispensable to pathogenicity, and understanding the complex virulence mechanism in pathogens. So, the VirulentPred tool was used to predict the virulence factors amongst 344 HPs and it was found that 62 proteins were most virulent (virulent score >1.0). The results are shown in Table 1. We have successfully assigned a proposed function to most virulent HPs from *O. tsutsugamushi* str. Karp*.* We used PFP and ARGOT^2^ server, which are sequence similarity-based protein function prediction tools designed to predict GO annotations from protein sequences [38]. PFP server gives the results on the basis of PFP scores (Very high confidence: >20K, High confidence: >10K, and Low confidence: ≥ 100), while the ARGOT^2^ gives the results on the basis of confidence scores. After extensive analysis and compilation of annotated functions, we selected top scoring GO-annotation terms (Moleculer functions and Biological processs) from both tools, The details are given in Table 2.

**Table 1:** A list showing the properties of the 62 most virulent proteins among the 344 HPs which had virulence scores >1.0.

S.No	Accession No.	Amino acid Composition based		Dipeptide Composition Based		PSI-BLAST created PSSM Profiles		Higher order Dipeptide Composition Based		Cascade of SVMs and PSI-BLAST		Average Scores
		Results	Scores	Results	Scores	Results	Scores	Results	Scores	Results	Scores	
1	KJV57131	Virulent	0.9178	Virulent	2.2957	Virulent	1.2206	Virulent	2.6358	Virulent	0.9184	1.59766
2	KJV57379	Virulent	1.5868	Virulent	1.7882	Virulent	1.4959	Virulent	1.2774	Virulent	1.5373	1.53712
3	KJV57416	Virulent	1.511	Virulent	1.3622	Virulent	1.5987	Virulent	2.0421	Virulent	0.9692	1.49664
4	KJV50735	Virulent	1.0381	Virulent	1.8509	Virulent	1.0601	Virulent	2.2208	Virulent	0.8616	1.4063
5	KJV55958	Virulent	1.0906	Virulent	1.6763	Virulent	1.4196	Virulent	1.8863	Virulent	0.9477	1.4041
6	KJV50994	Virulent	0.9864	Virulent	1.8571	Virulent	1.0425	Virulent	2.2675	Virulent	0.8448	1.39966
7	KJV53007	Virulent	0.9266	Virulent	1.8414	Virulent	1.1017	Virulent	2.2594	Virulent	0.8394	1.3937
8	KJV52681	Virulent	1.4108	Virulent	1.873	Virulent	1.1693	Virulent	1.4896	Virulent	1.0128	1.3911
9	KJV56935	Virulent	0.9485	Virulent	1.8072	Virulent	1.1589	Virulent	2.1562	Virulent	0.8693	1.38802
10	KJV54168	Virulent	1.2096	Virulent	1.6667	Virulent	1.2093	Virulent	1.7423	Virulent	1.021	1.36978
11	KJV53284	Virulent	1.3103	Virulent	1.0041	Virulent	1.2612	Virulent	2.2527	Virulent	0.9769	1.36104
12	KJV57348	Virulent	0.9076	Virulent	1.772	Virulent	1.4456	Virulent	1.6504	Virulent	0.9341	1.34194
13	KJV54735	Virulent	1.4234	Virulent	1.5279	Virulent	1.4345	Virulent	1.1387	Virulent	1.085	1.3219
14	KJV53188	Virulent	1.08	Virulent	1.5717	Virulent	1.1006	Virulent	1.8276	Virulent	1.0163	1.31924
15	KJV55734	Virulent	0.9327	Virulent	1.1912	Virulent	1.2217	Virulent	2.2464	Virulent	0.963	1.311
16	KJV53939	Virulent	1.3339	Virulent	1.511	Virulent	1.0233	Virulent	1.551	Virulent	1.0762	1.29908
17	KJV55659	Virulent	1.2404	Virulent	1.8791	Virulent	0.8758	Virulent	1.4898	Virulent	0.9889	1.2948
18	KJV54587	Virulent	1.6516	Virulent	1.4787	Virulent	1.2528	Virulent	1.019	Virulent	1.0679	1.294
19	KJV54139	Virulent	1.4621	Virulent	1.2146	Virulent	1.1112	Virulent	1.51	Virulent	1.1151	1.2826
20	KJV56583	Virulent	1.3002	Virulent	0.9569	Virulent	1.5489	Virulent	1.4896	Virulent	1.0944	1.278
21	KJV54508	Virulent	1.3796	Virulent	1.0061	Virulent	1.4037	Virulent	1.439	Virulent	1.1215	1.26998
22	KJV55874	Virulent	1.3446	Virulent	0.9225	Virulent	1.5398	Virulent	1.3943	Virulent	1.1028	1.2608
23	KJV55533	Virulent	1.239	Virulent	1.8973	Virulent	0.8824	Virulent	1.2263	Virulent	1.0019	1.24938
24	KJV56683	Virulent	1.3132	Virulent	1.2083	Virulent	0.9426	Virulent	1.6811	Virulent	1.075	1.24404
25	KJV57311	Virulent	0.9809	Virulent	1.7789	Virulent	1.1815	Virulent	1.3329	Virulent	0.8875	1.23234
26	KJV56036	Virulent	1.3556	Virulent	1.8793	Virulent	0.8037	Virulent	1.1186	Virulent	0.9595	1.22334
27	KJV53935	Virulent	1.3824	Virulent	0.9454	Virulent	1.142	Virulent	1.4598	Virulent	1.1236	1.21064
28	KJV55746	Virulent	1.0308	Virulent	1.5521	Virulent	0.9978	Virulent	1.373	Virulent	1.0921	1.20916
29	KJV56143	Virulent	1.1156	Virulent	1.0511	Virulent	1.1646	Virulent	1.5631	Virulent	1.1072	1.20032
30	KJV57393	Virulent	0.9855	Virulent	1.159	Virulent	1.2236	Virulent	1.5162	Virulent	1.0911	1.19508
31	KJV52864	Virulent	1.2226	Virulent	1.4901	Virulent	1.0702	Virulent	1.0677	Virulent	1.102	1.19052
32	KJV57120	Virulent	1.4178	Virulent	0.9323	Virulent	1.4383	Virulent	0.967	Virulent	1.0375	1.15858
33	KJV54670	Virulent	1.1082	Virulent	1.3983	Virulent	0.9956	Virulent	1.1537	Virulent	1.1142	1.154
34	KJV57203	Virulent	1.023	Virulent	1.0318	Virulent	1.0671	Virulent	1.5494	Virulent	1.0971	1.15368
35	KJV56211	Virulent	1.086	Virulent	1.2792	Virulent	1.08	Virulent	1.1946	Virulent	1.124	1.15276
36	KJV51002	Virulent	1.1881	Virulent	1.1188	Virulent	1.0868	Virulent	1.2358	Virulent	1.1316	1.15222
37	KJV56684	Virulent	1.0339	Virulent	0.9725	Virulent	1.195	Virulent	1.4313	Virulent	1.1025	1.14704
38	KJV50818	Virulent	1.2394	Virulent	1.081	Virulent	0.9281	Virulent	1.2893	Virulent	1.1164	1.13084
39	KJV53916	Virulent	1.1855	Virulent	0.9014	Virulent	1.0891	Virulent	1.3351	Virulent	1.1172	1.12566
40	KJV52751	Virulent	1.2581	Virulent	0.6884	Virulent	1.3906	Virulent	1.1715	Virulent	1.0925	1.12022
41	KJV57626	Virulent	1.1033	Virulent	0.9177	Virulent	1.2272	Virulent	1.104	Virulent	1.0994	1.09032
42	KJV50787	Virulent	0.821	Virulent	1.134	Virulent	0.8971	Virulent	1.4924	Virulent	1.0829	1.08548
43	KJV54906	Virulent	1.1008	Virulent	1.138	Virulent	0.9251	Virulent	1.1233	Virulent	1.1113	1.0797
44	KJV57212	Virulent	0.736	Virulent	1.3844	Virulent	0.7176	Virulent	1.4391	Virulent	1.0767	1.07076
45	KJV57216	Virulent	1.199	Virulent	1.4498	Virulent	1.3006	Virulent	0.4069	Virulent	0.9915	1.06956
46	KJV52478	Virulent	1.1052	Virulent	0.6497	Virulent	1.2606	Virulent	1.2378	Virulent	1.082	1.06706
47	KJV56675	Virulent	0.9893	Virulent	1.0009	Virulent	0.7595	Virulent	1.507	Virulent	1.0611	1.06356
48	KJV50671	Virulent	1.1341	Virulent	0.8286	Virulent	1.0583	Virulent	1.1183	Virulent	1.0939	1.04664
49	KJV54785	Virulent	0.9488	Virulent	0.9501	Virulent	0.9662	Virulent	1.2497	Virulent	1.0915	1.04126
50	KJV55465	Virulent	1.2074	Virulent	0.6517	Virulent	0.8766	Virulent	1.4166	Virulent	1.0525	1.04096
51	KJV52046	Virulent	1.125	Virulent	0.4258	Virulent	1.0812	Virulent	1.5058	Virulent	1.0433	1.03622
52	KJV53129	Virulent	1.3451	Virulent	0.9869	Virulent	1.2255	Virulent	0.5632	Virulent	1.055	1.03514
53	KJV54170	Virulent	1.0829	Virulent	0.9067	Virulent	1.0249	Virulent	1.0456	Virulent	1.0894	1.0299
54	KJV57117	Virulent	1.2103	Virulent	1.004	Virulent	1.1659	Virulent	0.6582	Virulent	1.0633	1.02034
55	KJV57230	Virulent	0.7618	Virulent	1.4071	Virulent	0.9733	Virulent	0.8748	Virulent	1.0668	1.01676
56	KJV57144	Virulent	0.7842	Virulent	0.9406	Virulent	0.8525	Virulent	1.4167	Virulent	1.0648	1.01176
57	KJV56401	Virulent	1.3022	Virulent	1.3618	Virulent	0.821	Virulent	0.5723	Virulent	0.9869	1.00884
58	KJV54671	Virulent	1.4225	Virulent	0.7659	Virulent	0.9325	Virulent	0.8532	Virulent	1.0672	1.00826
59	KJV51409	Virulent	0.9735	Virulent	0.8943	Virulent	1.05	Virulent	1.0433	Virulent	1.0738	1.00698
60	KJV57217	Virulent	1.0185	Virulent	0.8513	Virulent	0.9405	Virulent	1.1414	Virulent	1.0786	1.00606
61	KJV53065	Virulent	0.9979	Virulent	0.7991	Virulent	0.9951	Virulent	1.1638	Virulent	1.0733	1.00584
62	KJV54489	Virulent	0.9321	Virulent	1.2993	Virulent	0.7014	Virulent	1.0264	Virulent	1.069	1.00564

**Table 2:** Molecular functions (MF) and Biological Processes (BP) of the 62 most virulent proteins.

S.No	ACC.NO	Molecular Functions (MF)	Biological Processes (BP)
1	KJV57131	Protein binding, transferase activity, protein serine/threonine kinase activity	Cell adhesion, protein phosphorylation & ubiquitination
2	KJV57379	Protein binding, protein tyrosine phosphatase activity	Response to stress, peptidyl-tyrosine dephosphorylation
3	KJV57416	Cation binding	Porphyrin-containing compound biosynthetic process
4	KJV50735	Manganese ion binding	Proteolysis, fatty acid metabolic process
5	KJV55958	Nucleotide binding	Protein phosphorylation
6	KJV50994	Protein binding, zinc ion binding	Proteolysis, protein phosphorylation
7	KJV53007	Protein binding, ATP binding	Transcription, DNA-dependent, protein phosphorylation,
8	KJV52681	Nucleotide binding, 3′-5′ exonuclease activity, catalytic activity	Ribonucleoprotein complex biogenesis, nucleic acid phosphodiester bond hydrolysis
9	KJV56935	Nucleic acid binding	Transcription, DNA-dependent, protein phosphorylation
10	KJV54168	Protein kinase activity, transcription factor activity, sequence-specific DNA binding	Protein phosphorylation, regulation of transcription, DNA-templated
11	KJV53284	Protein binding	Transcription, DNA-dependent
12	KJV57348	Nucleotide binding, pullulanase activity, hydrolase activity	Transcription, DNA-dependent, carbohydrate metabolic process
13	KJV54735	Catalytic activity	L-methionine salvage, nucleoside metabolic process
14	KJV53188	Nucleotide binding	Metabolic process, translational termination
15	KJV55734	Protein binding, kinase activity, transferase activity	Transcription, DNA-dependent
16	KJV53939	Nucleotide binding, NAD + ADP-ribosyl transferase activity	Macromolecule metabolic process, protein phosphorylation
17	KJV55659	Protein binding, transferase activity, transferring acyl groups	Metabolic process, cell adhesion
18	KJV54587	Structural constituent of ribosome, DNA binding	Translation, cell redox homeostasis
19	KJV54139	Nucleotide binding, cell surface receptor signaling pathway	Signaling, signal transducer activity
20	KJV56583	Nucleic acid binding,	Transcription, DNA-dependent, cellular biogenic amine metabolic process
21	KJV54508	Nucleotide binding, cysteine-type peptidase activity	Amino acid activation, proteolysis
22	KJV55874	Ligase activity, calcium ion binding	Metabolic process, axial cellular bud site selection
23	KJV55533	Nucleotide binding, transferase activity, transferring acyl groups	Amino acid activation, RNA phosphodiester bond hydrolysis, endonucleolytic
24	KJV56683	Nucleotide binding, peptidoglycan binding	Transcription, DNA-dependent, cell division
25	KJV57311	Protein binding, hydrolase activity	Metabolic process
26	KJV56036	Structural molecule activity, DNA binding, hydrolase activity	Viral penetration into host nucleus, protein polyubiquitination
27	KJV53935	Catalytic activity, nucleic acid binding	RNA processing, phosphatidylinositol phosphorylation proteolysis
28	KJV55746	Nucleic acid binding, sequence-specific DNA binding	Transcription, DNA-dependent
29	KJV56143	Uroporphyrinogen-III synthase activity	Tetrapyrrole biosynthetic process
30	KJV57393	Nucleotide binding	Chorismate metabolic process
31	KJV52864	Protein binding, translation elongation factor activity	Transcription, translational elongation, peptide biosynthetic process
32	KJV57120	ATP binding, peptidase activity	Purine nucleotide biosynthetic process, proteolysis
33	KJV54670	ATP binding	Metabolic process
34	KJV57203	Nucleotide binding, DNA binding	Chorismate metabolic process, transposition, DNA-mediated
35	KJV56211	DNA binding, DNA-directed RNA polymerase activity	Transcription, DNA-dependent
36	KJV51002	ATP binding, phosphorylation	Transcription, DNA-dependent, kinase activity
37	KJV56684	Galactosyltransferase activity, aminoacyl-trna ligase activity, glycine-trna ligase activity	Metabolic process
38	KJV50818	Protein binding, lysozyme activity, hydrolase activity	Nucleic acid metabolic process, peptidoglycan catabolic process
39	KJV53916	Receptor binding	Defense response
40	KJV52751	ATP binding, nucleotide binding	Transcription, DNA-dependent
41	KJV57626	Phosphotransferase activity, nitrogenous group as acceptor, kinase activity	Signal transduction, phosphorylation
42	KJV50787	Nucleotide binding, metallopeptidase activity, metalloendopeptidase activity, hydrolase activity, metallopeptidase activity	Purine nucleotide biosynthetic process, oxidation-reduction process, methanogenesis
43	KJV54906	Catalytic activity, DNA-directed DNA polymerase activity, nucleotidyl transferase activity	Tetrapyrrole metabolic process, oxidation-reduction process, DNA biosynthetic process, DNA replication
44	KJV57212	Transporter activity, aspartic-type endopeptidase activity, hydrolase activity, peptidase activity	Transport, protein processing, proteolysis
45	KJV57216	Ion binding, hydrolase activity	Proteolysis, transcription, DNA-templated
46	KJV52478	Ion binding, transferase activity	Metabolic process, drug transmembrane transport, regulation of transcription
47	KJV56675	Nucleotide binding, mannose binding, carbohydrate binding	Metabolic process, regulation of defense response to virus by virus,
48	KJV50671	Transferase activity, transferring phosphorus-containing groups, signal transducer activity	Phosphatidylinositol-mediated signaling, cell surface receptor signaling pathway
49	KJV54785	Protein binding, actin binding, tropomyosin binding, sequence-specific DNA binding	Transcription, DNA-dependent, cellular component organization
50	KJV55465	Electron carrier activity, NADH dehydrogenase (ubiquinone) activity	Electron transport chain, oxidation-reduction process
51	KJV52046	Zinc ion binding	Proteolysis
52	KJV53129	Protein binding, metalloendopeptidase activity, hydrolase activity, metallopeptidase activity	Cellular response to stimulus, transcription, DNA-templated
53	KJV54170	ATP binding, protein kinase activity	Trehalose metabolic process, protein phosphorylation
54	KJV57117	DNA binding, DNA-directed RNA polymerase activity, proteolysis	Transcription, DNA-dependent, peptidase activity
55	KJV57230	Nucleotide binding, hydrogen ion transmembrane transporter activity	Amino acid activation, proton transport
56	KJV57144	Nucleotide binding, zinc ion binding	Metabolic process, protein phosphorylation
57	KJV56401	Nucleotide binding, transferase activity	Amino acid activation, peptidyl-aspartic acid modification, protein phosphorylation
58	KJV54671	ATP binding	Metabolic process
59	KJV51409	Nucleotide binding, single-stranded DNA binding	Transcription, DNA-dependent, SOS response, DNA replication, DNA repair
60	KJV57217	Nucleotide binding, hydrolase activity, nuclease activity, exonuclease activity	Metabolic process, cellular response to DNA damage stimulus, vesicle fusion with Golgi apparatus, intracellular protein transport
61	KJV53065	Transferase activity	Pyrimidine-containing compound biosynthetic process
62	KJV54489	ATP binding,	Transcription, DNA-dependent, protein deubiquitination

### 3.2 ROC (Receiver Operating Characteristic) performance measurement

**Fig. 2:**
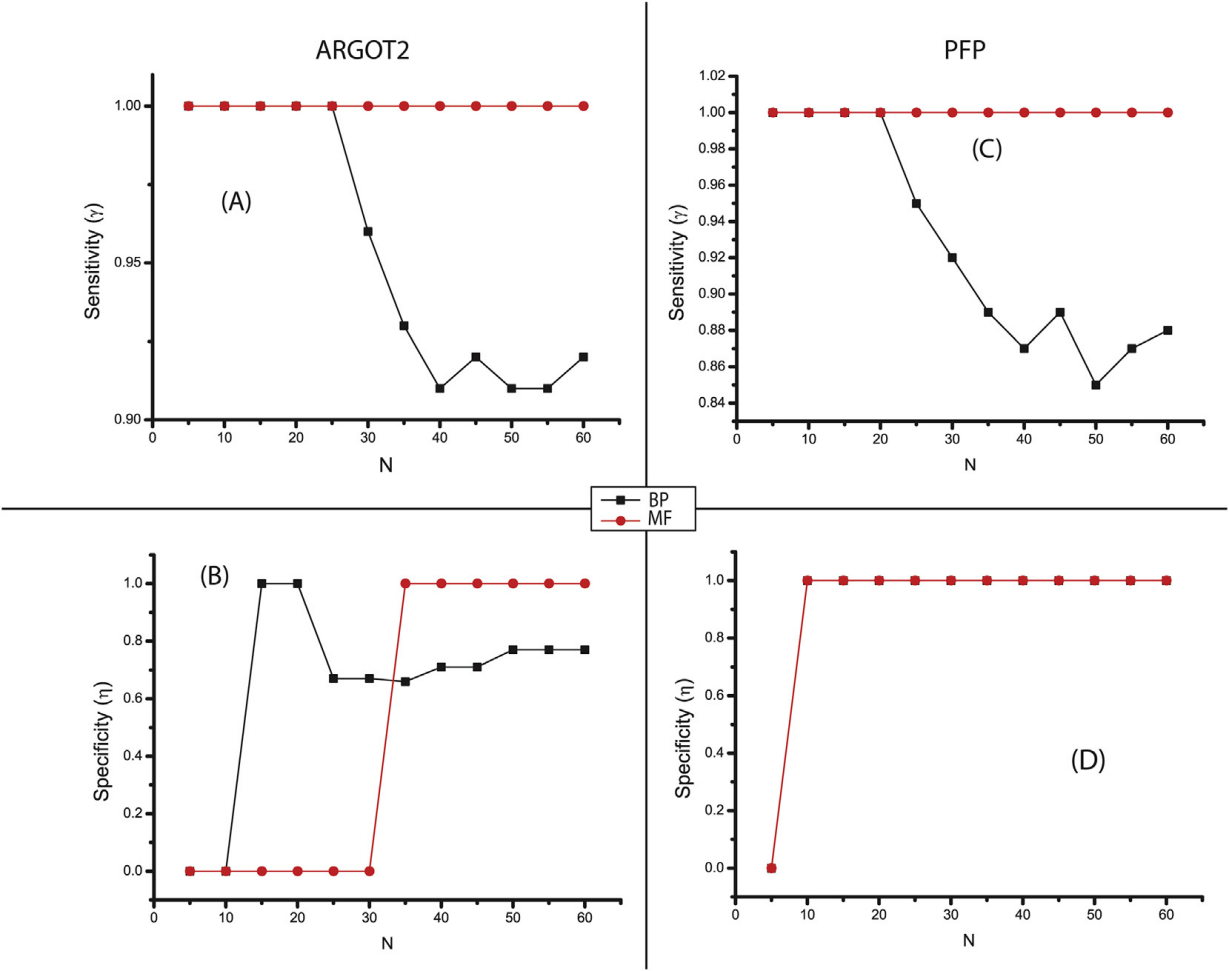
ROC plots presenting the change of trend of specificity and sensitivity at different sample size (N) respectively for both Argot2 and PFP tools. Where A,B refers to Argot2 and C,D to PFP. Black lines refer to Biological process (BP) and red lines refer to Molecular function (MF).

As the number of genome sequences available increases, more protein products that can be computationaly processed for further study become available. If autonomic computing predictions are solely depended on, it is important that the accuracy in the methods is high. There are various available conventional methods for comparing the tool's accuracy, but the ROC analysis is a widely used method. As suggested from the plots (Fig. 2a–d) the effect of sample size (N) can not be seen on both sensitivity (γ) and specificity (η) for both the tools as far as Molecular function (MF) is concerned. The decrease in γ is followed by an increase in η for biological process (BP). As seen in the plot 2A (for ARGOT2) the $\gamma^{MF}=1.0$, while $\gamma^{BP}$ ranges between 0.94<γ<1.0; in case of specificity (plot 2B) $\eta^{MF}$ shows a triphasic pattern that increases to a value of 1.0 then decreases in the range of 0.6<η<0.75 while$\;\eta^{BP}$ showing a biphasic pattern that does not showing an effect up to N=30; while above this (i.e. N>30) it becomes highly specific. In addition to this plot 2C (for PFP) the $\gamma^{MF}=1.0$ while $\gamma^{BP}$ ranges between 0.86<γ<1.0; in case of specificity (plot 2D) $\eta^{MF}$ & $\eta^{BP}$ show constancy in their behaviour. Thus, it can be said that for the prediction of MF, the sample size (N) behaves as an independent variable. On the other hand, for BP the trend shown by the plots satisfies N to be an intrinsic property. Thus, from these plots and the ranges they fall in we can infer that large sample size and a combination of these tools can offer the promise of predicting accuracy for both MF and BP. The average sensitivity are 1.00 (Molecular function) and 0.955 (Biological processes) and the average specificity are 0.5 (moleculaer function) and 0.64 (biological processes) from ARGOT^2^ tool. Similarly, for PFP server, the the average sensitivity are 1.00 (Molecular function) and 0.922 (Biological processes) and the average specificity are 0.916 (moleculaer function) and 0.916 (biological processes), shown in Table 3. So, the overall accuracy of both the tools, PFP and ARGOT^2^ server was found to be satisfactory.

**Table 3:** Sensitivity and specificity at various cut-off points for the prediction of functionally, annotated HPs and Biological processes.

S.No.	SAMPLE SIZE		TRUE POSITIVE(a)		TRUE NEGATIVE(d)				FALSE POSITIVE(c)				FALSE NEGATIVE(b)				Sensitivity				Specificity	
**ARGOT2**																						
1	0		**MF**	**BP**	**MF**		**BP**		**MF**		**BP**		**MF**		**BP**			**MF**		**BP**	**MF**	**BP**
2	5		5	5	0		0		0		0		0		0			1		1.0	0.0	0.0
3	10		10	10	0		0		0		0		0		0			1		1.0	0.0	0.0
4	15		15	14	0		1		0		0		0		0			1		1.0	0.0	1.0
5	20		20	19	0		1		0		0		0		0			1		1.0	0.0	1.0
6	25		25	22	0		2		0		1		0		0			1		1.0	0.0	0.67
7	30		30	24	0		3		0		2		0		1			1		0.96	0.0	0.67
8	35		34	27	1		4		0		2		0		2			1		0.93	1.0	0.66
9	40		37	30	3		5		0		2		0		3			1		0.91	1.0	0.71
10	45		42	35	3		5		0		2		0		3			1		0.92	1.0	0.71
11	50		46	37	4		7		0		2		0		4			1		0.91	1.0	0.77
12	55		50	42	5		7		0		2		0		4			1		0.91	1.0	0.77
13	60		54	47	6		7				2				4			1		0.92	1.0	0.77
**PFP Function**																						
1	0	**MF**		**BP**		**MF**		**BP**		**MF**		**BP**		**MF**		**BP**			**Sensitivity**		**Specificity**	
2	5	5		5		0		0		0		0		0		0			1	1.0	0.0	0.0
3	10	9		8		1		2		0		0		0		0			1	1.0	1.0	1.0
4	15	13		11		2		4		0		0		0		0			1	1.0	1.0	1.0
5	20	18		16		2		4		0		0		0		0			1	1.0	1.0	1.0
6	25	22		18		3		6		0		0		0		1			1	0.95	1.0	1.0
7	30	27		22		3		6		0		0		0		2			1	0.92	1.0	1.0
8	35	31		24		4		8		0		0		0		3			1	0.89	1.0	1.0
9	40	36		26		4		10		0		0		0		4			1	0.87	1.0	1.0
10	45	41		31		4		10		0		0		0		4			1	0.89	1.0	1.0
11	50	45		34		5		10		0		0		0		6			1	0.85	1.0	1.0
12	55	50		39		5		10		0		0		0		6			1	0.87	1.0	1.0
13	60	55		41		5		11		0		0		0		6			1	0.88	1.0	1.0

MF:Molecular Function, BP:Biological Process

### 3.3 Sequence similarity networks (SSN)

Sequence similarity networks (SSN) have emerging importance because they allow comparative analysis of mammoth datasets without the need for multiple sequence alignments (MSA). Currently, it has become more dependable as datasets constantly increase in size. The greater number of pairwise relationships determined within the networks leads to a more accurate placement of sequences among putative homologs [39]. In this study, we constructed a network with 62 most virulent proteins on which only 21 proteins interacted with others and the rest were determined as outlier proteins in the network, so we eliminated them from the network. These 21 highly identical proteins are from the same *O. tsutsugamushi* (strain Boryong) family. The topological parameters of the network obey power law distributions. The probability of clustering co-efficient *C(k)*, degree distributions *P(k)*, and neighborhood connectivity *C*_*N*_*(k)* exhibit a fractal nature**.** The power law fits on the data points of the network's topological parameters were done and confirmed by following the standard statistical fitting method given by Clauset et al [40] where the p values for all data sets were calculated (against 2500 random samplings) and found to be greater than 0.1 and data fitting goodness was less. **P(k)** and **C(k)** have negative values (-0.556 and -0.245 respectively) which implies the network follows a hierarchical pattern and positive-value of (**C**_**N**_**(k)** = 0.756) that means the network follows the assortativity that identifies a huge cluster of degree-nodes (rich club) which regulates the network. The centrality parameters: betweenness (C_B_ = 1.170) and closeness (C_C_ = 0.152) of the network also showed fractal behaviour and good connectivity of nodes in a network.

**Fig. 3:**
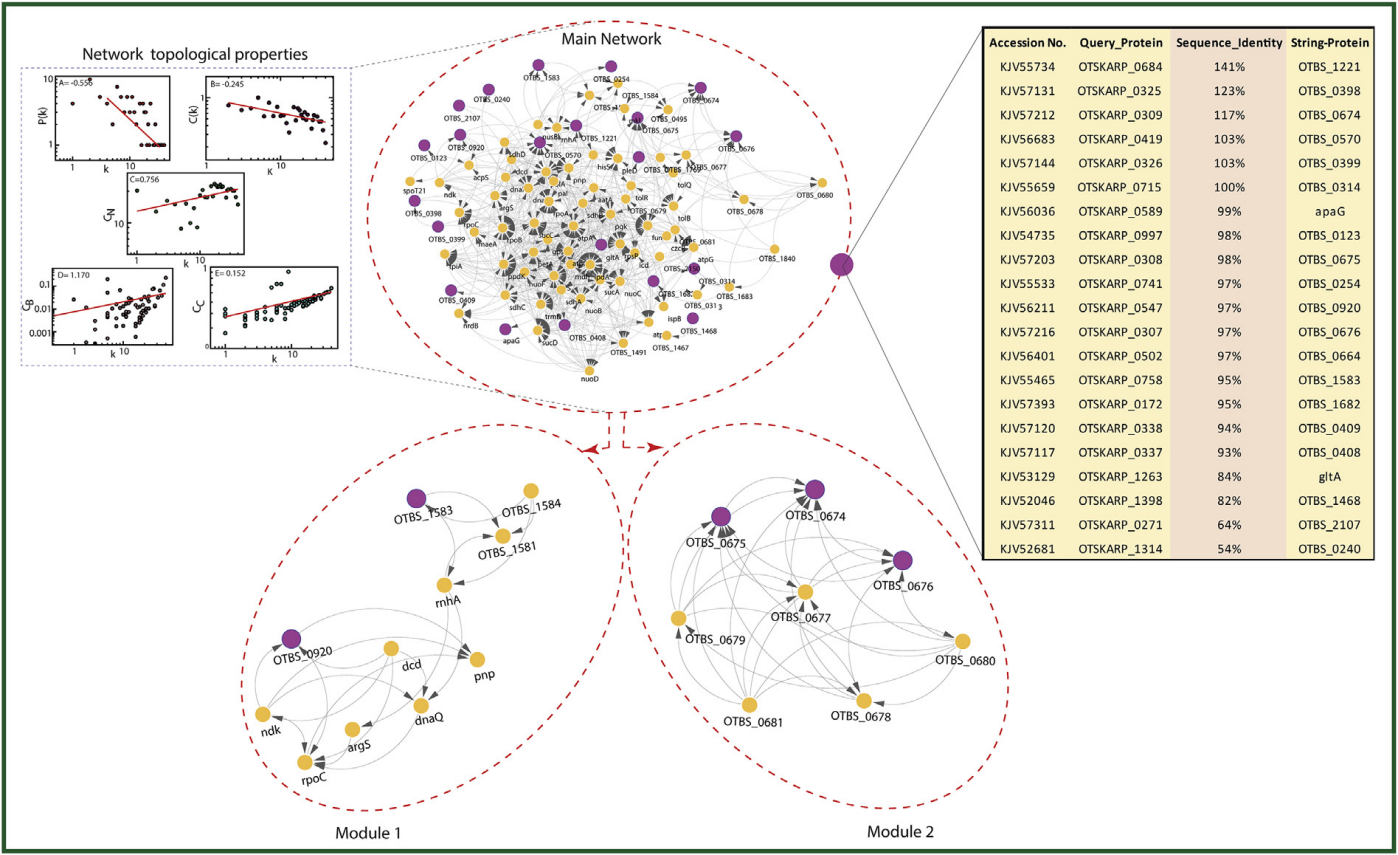
Protein-Protein interaction: Sequence similarity network (cluster coff. = 0.6) was constructed by STRING database using 62 most virulent proteins, but out of which only 21 proteins (Magenta colour nodes) interact with other proteins (yellow nodes). The toplogical properteies show the hierarchical pattern of the network, The behaviours of degree distributions (P(k)), clustering co-efficient (C(k)), neighborhood connectivity (CN(k)), betweenness (CB(k)) and closeness (CC(k)) measurements as a function of degree k. The lines are fitted lines with power laws in the data sets. The parent network was broken into 2 highly interconnected modules (cluster coff. = 1) which contains five hub proteins namely KJV55465(OTBS_1583),KJV56211(OTBS_0920), KJV57212(OTBS_0674), KJV57203 (OTBS_0675) and KJV57216 (OTBS_0676).

The characterizing modular structure of a biological network is an important way to identify novel genes for targeted therapeutics. So, we have identified 2 modules (highly interconnected regions) in the parent network using MCODE(v1.5.1) in Cytoscape. These modules contain five proteins (*OTBS_1583, OTBS_0920, OTBS_0674, OTBS_0675, OTBS_0676*) that were considered the hub proteins in the network, shown in Fig. 3. Since the popularity of leading hubs gets changed according to the protein activities and its regulation, it cannot be determined whether these hub proteins are key regulators but some may play a significant role in pathogen survival. In the first module, the hub proteins OTBS_1583 and OTBS_0920 interacted with nine other proteins including *OTBS_1584, OTBS_1581, mhA, pnP, dcD, ndK, rpoC, argS, dnaQ.* While in the second moldule, the hub proteins are OTBS_0674, OTBS_0675 and OTBS_0676 interatced with *OTBS_0677, OTBS_0678, OTBS_0679, OTBS_0680*, and *OTBS_0681.*

### 3.4 Protein structures prediction

**Fig. 4:**
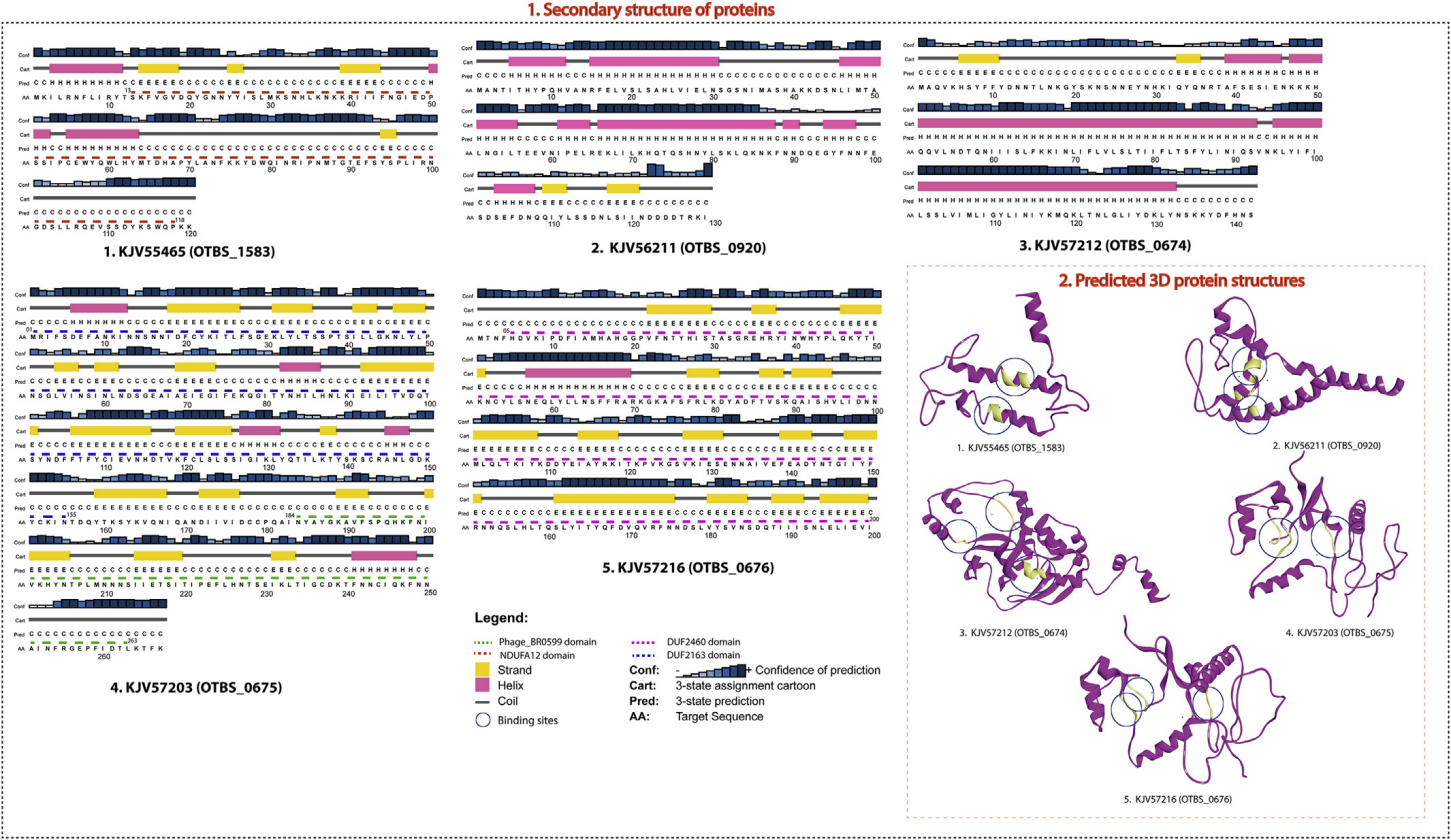
Secondary structure of hub proteins predicted with the Psipred server. (1) The Psipred model showed; alpha helices, strand and coil regions and result accuracy. The confidence prediction scores are shown in the blue, grey or black bars. The red dashed line shows the conserved functional domain in the proteins. (2) The 3D structure of the five hub proteins are modelled by MODELLER and the predicted probable binding sites are circled (oily yellow) in the structures.

Secondary structures were predicted with the Psipred server. The protein *KJV55465* was observed to be organized in long coil regions interrupted with short beta sheets and alpha helices, while proteins *KJV57203* and *KJV57216* were organised in large strands and coils. Two proteins *KJV56211* and *KJV57212* were well organised in long alfa helices interrupted with short coil regions. For prediction of proteins strutcurs, the most identical templates (PDB_Blast) against each of the hub proteins was considered then tertiary structures were built using the Modeller. On the basis of the lowest value of the DOPE assessment score, or the highest GA341 assessment score, the “best” model was selected. The details are given in Table 4. Since it is known that Ligand binding is required for many proteins to function properly, the most probable binding sites amongst the hub proteins identified in this study using the ASP server (Active Site Prediction) [41]. The binding site of the five hub proteins, indicate that the residues in the active site are as follows: (i) KJV55465 (*L-104, L-105, R-106*; *I-41, I-42, F-44,N-45*). (ii) KJV56211 (*H-77, L-80, Y-111; H-72, Q-73, Q-75; K-71, T-74, S-76*). (iii) KJV57212 (*N-171, D-172, I-174, V-175; K-77, Q-78, G-79; N-14, S-15, N-16, N-17, T-18*). (iv) KJV57203 (*M-101, L-102, Q-103,L-104*; *D-81, Y-82, A-83*; *E-140, A-141, D-142*). (V) KJV57216 (*D-81, N-176, D-177*; *M-101*, *L-102, Q-103*; *F-139, D-142*), the details are given in Fig. 4. The functional domains of the hub proteins were predicted using Pfam databse [42]. In the protein *“KJV55465”*, the NDUFA12 domain was found between residues 13-118, which is an accessory subunit of the mitochondrial membrane respiratory chain NADH dehydrogenase (Complex I) [43]. Complex-I is found in bacteria and cyanobacteria (as a NADH-plastoquinone oxidoreductase) that is a main source of reactive oxygen species (ROS) predominantly formed by electron transfer from FMNH. Similarly, the *“KJV57203”* protein has two domains: (i) DUF2163 (between 01-155 residue) (ii) Phage_BR0599 (between 184-263 residue). Phage conserved hypothetical protein “BR0599” is found almost exclusively in phage or in prophage regions of bacterial genomes, including the phage-like *Rhodobacter capsulatus* gene transfer agent, which packages DNA. The protein *“KJV57216”* has only one domain namely DUF2460. The domains “DUF2163” and “DUF2460” are uncharacterized conserved proteins but their functions are still unknown. However, no positive or negative evidence was found regarding the functional domain of these proteins *“KJV56211”* and *“KJV57212”*.

**Table 4:** Summary of the best model produced with the lowest DOPE score and highest GA341 assessment score.

S.No.	Modelled Proteins	DOPE score	GA341 score
1	*KJV55465* (OTBS_1583)	-6911.375	0.00210
2	*KJV56211* (OTBS_0920)	-8583.088	0.04392
3	*KJV57212* (OTBS_0674)	-12852.596	0.04440
4	*KJV57203* (OTBS_0675)	-20830.900	0.16169
5	*KJV57216* (OTBS_0676)	-13358.363	0.03311

## 4 Discussion

*Orientia tsutsugamushi* is an obligate intracellular bacterium transmitted to humans via the bite of an infected trombiculid mite [44]. Here, the main goal of our study was to determe the protein functions from the hypothetical protein sequences. Functional annotation of 344 hypothetical proteins are of major importance in providing insight into their molecular functions and will help us in the identification of new drugs against the disease. The subcellular localization of these proteins is important to understand their interactions with drug molecules and other proteins. In our study, we found that most of the HPs exist in the cytoplasmic region (75%), inner membrane regions (3.45%), outer membrane regions (8.43%), extra cellular regions (8.13%) and in the periplasmic region (4.36%). Further we classified these 344 HPs into various protein classes like DNA binding proteins, transporter proteins, enzymes, outer membrane proteins, and many other proteins. It has been studied that the DNA-binding proteins play an important role in bacterial stress tolerance and survival in the host and may be responsible for virulence [45, 46]. It is known that enzymes play important roles in pathogen virulence (like bacterial cell-wall & ubiquinone biosynthesis, antibiotic resistance (β-lactam), invasion, and intracellular replication) [47, 48, 49]. Besides enzymes, transporter proteins are also crucial for the intracellular survival of bacteria as they efflux drug molecules out of the bacterial cell. Therefore, all the classified proteins are important for the survival and pathogenicity of the pathogen. The present investigation mainly focused only on identifying virulence factors among these hypothetical proteins. From 344 hypothetical proteins only 62 proteins were found to be most virulent and these virulence properties may help in the pathogen's survival. Therefore these proteins may be therapeutic drug-targets to combat pathogens. It has already been reported that virulent proteins were targeted for drug discovery and development [50]. Further, these 62 virulent proteins were considered for sequence similarity network analysis. In network analysis, two modules and 05 hub genes were identified which closely interacted with important proteins:-

• **KJV55465 (OTBS_1583):** Is a protein 120 residues long known as “Putative NADH ubiquinone oxidoreductase”. It is mainly involed in electron transporter activity and NADH dehydrogenase (ubiquinone) activity. In network module, it was found to be interacting with *rnhA*, *OTBS_1581* and *OTBS_1584.* It well known that *rnhA* (Ribonuclease H) is involved in repairing processes in cases where RNA/DNA duplexes are generated during DNA replication [51]. The protein OTBS_1584 is a putative ABC transporter substrate binding protein which play roles in nutrient uptake and drug resistance. However, there is increasing evidence that these transport systems play either direct or indirect roles in the virulence of bacteria [52].
• **KJV56211 (OTBS_0920):** It is 120 residues long protein which intercted with ndK, rpoC, dcD and pnP. Nucleoside diphosphate kinase (Ndk) is an important enzyme which plays an important role in bacterial growth, signal transduction and pathogenicity [53]. The rpoC protein, which encodes the RNA polymerase β′ subunit that help to bacterial pathogen growth. The dcD is know as dCTP deaminase which catalyzes the deamination of dCTP to dUTP. The primary source of dUMP, the precursor for dTTP in the gram-negative bacteria is getting throgh a pathway where dCTP is deaminated by dcD to produce ammonia and dUTP that later hydrolyzed by dUTPase to generate dUMP and pyrophosphatea Similarly, the pnP is known as polyribonucleotide nucleotidyltransferase which is widely conserved and plays a major role in RNA decay in both gram-negative and gram-positive bacteria [54].
• **KJV57212 (OTBS_0674):** It is 142 long residues protein, which interacted with two hub proteins **OTBS_0675** (270 residue), **OTBS_0676** (199 residue) and these three proteins are synergistically interacted with other proteins like OTBS_0677 (uncharacterized protein), OTBS_0677 (uncharacterized protein), OTBS_0679 (uncharacterized protein), OTBS_0680 and OTBS_0681. In which OTBS_0680 and OTBS_0681 are known as phage major capsid protein (HK97) and phage prohead HK97 respectively.

## 5 Conclusion

The strategies used in our study to annotate functions of hypothetical proteins can be useful for designing experimental approaches geared towards the evolution of the exact function of the corresponding gene. Here, we have characterized and functionally annotated the 344 hypothetical proteins from *O. tsutsugamushi* str*.* Karp and categorized them into different protein classes. Among these 344 HPs, 62 proteins were found to be most virulent. Virulence refers to the severity of infection, and different toxins are produced by pathogenic bacteria to withstand the host immune system. In addition, we constructed a sequence similarity network to understand the interaction of these virulent proteins and identifed five hub proteins (*KJV55465, KJV56211, KJV57212, KJV57203 and KJV57216)* among them which play key regulatory and co-regulatory roles in the network. The conserverd domains of these hub proteins necessary for functional information, experimental design and genome-level annotation were analyzed. Conclusively secondary structure prediction and 3D modelling further provided insight into the spatial arrangement of the amino acids in the proteins to find the most probale binding sites for drugs. These HPs may serve as potential therapeutic targets and may be considered as a milestone in the emerging field of drug discovery. We hope that the information of HPs from *O. tsutsugamushi* will be innovative for further in-vitro analysis of this disease.

## Declarations

### Author contribution statement

Nikhat Imam: Conceived and designed the experiments; Performed the experiments; Wrote the paper.

Aftab Alam: Analyzed and interpreted the data; Wrote the paper.

Rafat Ali: Analyzed and interpreted the data.

Mohd Faizan Siddiqui: Performed the experiments; Analyzed and interpreted the data.

Sher Ali, Romana Ishrat: Conceived and designed the experiments.

Md. Zubbair Malik: Contributed reagents, materials, analysis tools or data.

### Funding statement

This research did not receive any specific grant from funding agencies in the public, commercial, or not-for-profit sectors.

### Competing interest statement

The authors declare no conflict of interest.

### Additional information

No additional information is available for this paper.
