# Supplementary material for: In silico characterization of hypothetical proteins from Orientia tsutsugamushi str. Karp uncovers virulence genes
Source: Heliyon. 2019 Nov 1;5(10):e02734. doi: 10.1016/j.heliyon.2019.e02734 (PMC6838952; doi:10.1016/j.heliyon.2019.e02734)
Supplement: Supplementary file 1 [file mmc1.pdf]

S1_Table: List of Computed Physicochemical Parameters of 344 HPs from *Orientia tsutsugamushi str. Karp*

S.no	Accession No.	Number of amino acids	Molecular weight	Theoretical PI	Extinction coefficients (M^{-1} cm^{-1})	Instability index		Aliphatic index	GRAVY
1.	KJV50500	35	4177.74	4.51	5960	16.74	Stable	100.29	-0.131
2.	KJV50518	87	10402.66	8.99	9970	22.3	Stable	55.98	-1.309
3.	KJV50599	107	11816.57	10.23	15595	18.96	Stable	122.99	0.501
4.	KJV50624	75	8911.67	10.09	14440	14.09	Stable	103.87	-0.304
5.	KJV50625	437	50414.32	5.88	48625	33.09	Stable	96.2	-0.421
6.	KJV50645	90	10390.44	10.15	5960	33	Stable	113.78	-0.128
7.	KJV50671	204	23644.56	4.31	24785	47.51	Unstable	97.01	-0.289
8.	KJV50672	133	15380.54	8.3	11710	44.47	Unstable	79.92	-0.725
9.	KJV50707	131	14970.42	9.67	9065	39.58	Stable	95.42	-0.21
10.	KJV50735	446	50848.12	5.72	39935	54.2	Unstable	93.14	-0.413
11.	KJV50787	179	19176.12	4.06	9190	32.25	Stable	85.42	-0.206
12.	KJV50815	177	20718.15	5.22	26610	30.04	Stable	88.7	-0.588
13.	KJV50818	188	22163	5.27	23295	41.49	Unstable	97.98	-0.431
14.	KJV50905	75	8839.61	10.19	14440	15.61	Stable	103.87	-0.263
15.	KJV50906	63	7536.01	11.66	4470	67.36	Unstable	117.62	-0.287
16.	KJV50907	55	6407.81	10.63	2980	27.34	Stable	118.55	0.12
17.	KJV50939	54	6292.21	10.18	6990	16.38	Stable	84.81	-0.6
18.	KJV50940	42	4593.53	5.51	12490	23.11	Stable	160	1.067
19.	KJV50970	117	13887.44	9.64	17545	61.61	Unstable	116.67	0.385
20.	KJV50994	443	50317.57	5.04	34560	60.69	Unstable	91.13	-0.384
21.	KJV50999	267	30774.86	5.41	41495	27.33	Stable	97.15	-0.346
22.	KJV51000	129	14469.91	5.59	11460	41.36	Unstable	110.39	-0.105
23.	KJV51002	91	10083.67	9.15	1615	13.53	Stable	104.95	-0.126
24.	KJV51003	87	10311.22	9.94	16960	41.28	Unstable	106.44	-0.04
25.	KJV51004	54	6360.33	4.27	4470	53.51	Unstable	137.04	0.428
26.	KJV51035	38	4205.9	9.63	1490	-4.37	Stable	64.21	0.066
27.	KJV51076	66	7813.38	10.29	8480	46.57	Unstable	109.24	-0.198

28.	KJV51080	42	5009.83	6.1	3105	63.89	Unstable	111.43	-0.119
29.	KJV51128	170	19774.7	9.39	13200	52.75	Unstable	70.53	-0.932
30.	KJV51131	622	72406.96	5.72	71140	39.73	Stable	94.82	-0.4
31.	KJV51134	196	23045.47	5.04	14900	69.35	Unstable	69.69	-1.056
32.	KJV51142	197	23360.75	9.2	17545	51.12	Unstable	84.06	-0.763
33.	KJV51205	266	29143.26	4.63	26735	29.76	Stable	73.08	-0.28
34.	KJV51252	344	38500.87	6.85	16305	42.36	Unstable	109.65	0.014
35.	KJV51253	76	9116.97	10	7450	15.79	Stable	130.79	-0.105
36.	KJV51286	45	5450.28	9.7	4470	53.51	Unstable	75.78	-0.951
37.	KJV51289	43	5074.76	4.99	5960	17.4	Stable	86.05	-0.379
38.	KJV51373	50	5637.37	4.53	4470	23.63	Stable	97.4	-0.322
39.	KJV51375	207	24721.81	9.02	30495	42.38	Unstable	84.35	-0.454
40.	KJV51376	622	71089.31	8.84	64540	47.75	Unstable	91.46	-0.501
41.	KJV51409	46	5317.29	9.14	4595	56.28	Unstable	95.43	-0.111
42.	KJV51411	75	8839.61	10.19	14440	15.61	Stable	103.87	-0.263
43.	KJV51578	136	16113.67	9.9	16180	30.97	Stable	91.76	-0.465
44.	KJV51579	107	11880.61	10.13	15595	16.5	Stable	125.7	0.534
45.	KJV51581	248	28205.89	9.29	33265	24.56	Stable	103.02	-0.128
46.	KJV51649	207	24586.4	8.57	30495	42.81	Unstable	81.98	-0.494
47.	KJV51694	44	4945.63	4.94	2980	37.19	Stable	92.95	-0.364
48.	KJV51695	82	9363.08	9.91	8940	1.86	Stable	96.22	-0.235
49.	KJV51776	42	4821.54	6.01	0	39.76	Stable	102.14	-0.595
50.	KJV51784	58	6868.8	7.84	4470	68.27	Unstable	53.79	-0.905
51.	KJV51788	95	10921.18	9.92	15720	15.36	Stable	120	0.057
52.	KJV51829	65	6550.8	10.11	11000	4.26	Stable	121.79	-0.264
53.	KJV51877	99	11659.74	9.68	11460	26.38	Stable	109.49	-0.174
54.	KJV51880	72	8510.29	10.47	16960	25.54	Stable	104.17	-0.204
55.	KJV51881	71	8674.05	6.37	16055	43.43	Unstable	111.13	0.201
56.	KJV51919	43	4748.43	4.53	3105	48.01	Unstable	115.58	0.184
57.	KJV51964	225	26245.1	6.1	38850	43.09	Unstable	98.04	-0.23
58.	KJV52046	95	11223.74	9.13	12950	33.3	Stable	85.05	-0.557
59.	KJV52048	94	11270.17	5.64	7575	33.93	Stable	107.87	-0.25

60.	KJV52143	25	2934.64	10.18	5500	8.65	Stable	101.2	-0.392
61.	KJV52144,	53	6232.55	11.05	4470	48.45	Unstable	119.3	0.328
62.	KJV52230	608	69920.19	6.07	65265	33.44	Stable	98.82	-0.314
63.	KJV52234	136	16228.5	5.95	21430	47.08	Unstable	103.09	-0.525
64.	KJV52319	31	3733.94	4.87	4470	36.17	Stable	43.87	-1.681
65.	KJV52373	266	30004.33	5.02	24005	39.84	Stable	95.3	-0.027
66.	KJV52376	113	13267.06	7.74	14565	34.24	Stable	91.33	-0.496
67.	KJV52426	46	5606.75	11.57	1490	93.5	Unstable	99.57	-0.65
68.	KJV52428	35	4218.93	8.03	2980	53.35	Unstable	80.86	-0.369
69.	KJV52477	178	20542.95	4.78	25120	37.69	Stable	88.2	-0.526
70.	KJV52478	35	4061.9	11.48	1490	26.68	Stable	111.43	-0.074
71.	KJV52571	52	6002.28	4.89	4470	27.03	Stable	151.73	0.885
72.	KJV52622	606	69531.36	5.14	54625	52.16	Unstable	87.9	-0.514
73.	KJV52681	35	4164.91	9.36	4470	14.06	Stable	89.14	-0.38
74.	KJV52748	122	13414.4	6.81	9970	31.03	Stable	111.97	0.154
75.	KJV52749	70	8485.79	6.27	16960	45.68	Unstable	90.57	0.21
76.	KJV52750	364	42214.31	4.77	46675	45.45	Unstable	83.57	-0.49
77.	KJV52751	222	25089.12	4.62	7575	41.66	Unstable	86.49	-0.351
78.	KJV52864	48	5661.74	9.16	4470	57.68	Unstable	117.71	0.054
79.	KJV52869	203	24322.94	9.69	20650	39.06	Stable	85.42	-0.78
80.	KJV52928	47	5791.6	7.84	15470	37.2	Stable	66.38	-0.866
81.	KJV53004	357	40221.98	4.82	44615	32.4	Stable	90.95	-0.388
82.	KJV53007	100	11406.95	7.78	13535	46.98	Unstable	91.6	0.062
83.	KJV53065	312	36193.47	5.78	37485	20.72	Stable	91.03	-0.55
84.	KJV53066	139	15955.15	8.6	10680	34.09	Stable	72.23	-0.59
85.	KJV53067	126	14580.44	4.6	18575	38.83	Stable	91.35	-0.357
86.	KJV53068	179	20153.47	9.06	7700	33.58	Stable	102.35	0.135
87.	KJV53073	113	13335.27	9.17	13075	40.72	Unstable	92.21	-0.491
88.	KJV53125	199	22693.95	9.32	7575	36.33	Stable	88.69	-0.65
89.	KJV53129	152	17839.54	9.24	16055	44.84	Unstable	93.55	-0.461
90.	KJV53187	129	14921.81	7.89	8940	48.86	Unstable	89.15	-0.75
91.	KJV53188	38	4635.46	9.52	1490	112.14	Unstable	97.37	-0.589

92.	KJV53189	139	16669.81	9.43	15275	51.2	Unstable	103.09	-0.219
93.	KJV53192	315	36484.44	6.67	53860	43.29	Unstable	95.56	-0.35
94.	KJV53203	183	20325.68	4.39	19535	46.32	Unstable	75.63	-0.296
95.	KJV53284	58	6958.12	9.99	6990	45.23	Unstable	97.59	-0.216
96.	KJV53363	107	11792.51	10.23	15595	17.34	Stable	122.06	0.512
97.	KJV53442	66	7782.35	9.76	2980	58.59	Unstable	125.45	0.042
98.	KJV53524	186	20287.23	7.86	4470	36.4	Stable	96.45	-0.231
99.	KJV53634	41	4749.62	9.1	1490	41.87	Unstable	99.76	0.324
100.	KJV53715	122	14361.06	10.37	5960	50.57	Unstable	99.84	-0.545
101.	KJV53716	188	21876.95	5.68	40910	41.64	Unstable	92.34	-0.289
102.	KJV53819	72	7996.26	4.02	1490	56.63	Unstable	65	-1.108
103.	KJV53820	59	8044.61	10.46	10095	23.65	Stable	97.39	-0.297
104.	KJV53914	97	11523.91	6.14	14440	50.34	Unstable	83.3	-0.822
105.	KJV53916	52	6065.32	10.84	1490	57.22	Unstable	105	0.023
106.	KJV53919	832	95465	6.97	68815	37.77	Stable	88.12	-0.58
107.	KJV53935	76	9141.88	11.43	4470	74.8	Unstable	115.39	-0.289
108.	KJV53937	861	102023.13	5.87	173720	40.31	Unstable	93.11	-0.439
109.	KJV53939	391	45153.09	4.83	57590	38.32	Stable	92.94	-0.287
110.	KJV54139	134	15412.75	7.58	13200	24.64	Stable	87.31	-0.581
111.	KJV54140	94	11020.27	10.64	5500	6.51	Stable	94.36	-0.657
112.	KJV54141	50	5760.78	9.82	11460	11.15	Stable	105.2	-0.176
113.	KJV54143	66	7687.3	5.25	2980	45.77	Unstable	144.7	0.435
114.	KJV54146	59	6622.49	9.82	43.29	43.29	Unstable	52.88	-1.207
115.	KJV54147	325	37604.55	5.7	73450	40.98	Unstable	99.85	-0.177
116.	KJV54167	859	98907.65	4.73	51200	46.13	Unstable	88.2	-0.55
117.	KJV54168	115	13424.75	4.34	23045	67.97	Unstable	68.61	-0.675
118.	KJV54170	50	5959.18	10.58	1490	15.04	Stable	83.8	-0.738
119.	KJV54341	40	4577.45	9.99	2980	9.93	Stable	107.25	-0.065
120.	KJV54343	85	20868.95	4.85	11125	42.23	Unstable	103.24	-0.311
121.	KJV54362	158	18188.86	5.93	12950	42.92	Unstable	94.37	-0.391
122.	KJV54368	79	9283.21	9.13	8940	65.94	Unstable	35.95	-1.449
123.	KJV54370	289	33790.71	5.37	40465	32.91	Stable	111.9	-0.072

124.	KJV54388	46	4990.81	8.25	0	50.95	Unstable	112.39	0.365
125.	KJV54398	40	4798.77	9.17	3105	12.81	Stable	102.25	-0.275
126.	KJV54413	170	20072.24	8.97	41410	29.9	Stable	84.94	-0.417
127.	KJV54438	102	11605.78	10.02	8605	44.12	Unstable	89.8	-0.419
128.	KJV54459	38	4378.19	4.17	2980	37.16	Stable	112.63	0.126
129.	KJV54463	97	10697.11	9.97	15595	21.75	Stable	121.65	0.602
130.	KJV54464	22	2455.99	10.66	0	21.16	Stable	88.64	-0.959
131.	KJV54465	66	7642.15	9.92	1490	47.5	Unstable	113.64	0.048
132.	KJV54466	42	4844.66	4.78	2980	25.12	Stable	113.81	0.357
133.	KJV54488	408	47984.24	8.97	37165	47.51	Unstable	79.34	-0.813
134.	KJV54489	86	10004.42	6.02	4470	45.62	Unstable	115.7	-0.328
135.	KJV54492	186	21938.73	4.39	33140	38.13	Stable	96.34	-0.285
136.	KJV54506	2219	251414.54	5.65	238265	35.25	Stable	87.12	-0.369
137.	KJV54508	47	5402.15	3.8	2980	63.4	Unstable	149.15	0.445
138.	KJV54527	50	5777.68	11.04	2980	42.9	Unstable	70	-0.954
139.	KJV54528	77	8575.95	9.45	6085	35.69	Stable	106.23	0.019
140.	KJV54529	90	9705.62	6.5	5500	36.07	Stable	135.33	1.147
141.	KJV54530	39	4468.15	5.98	5960	17.54	Stable	127.44	0.197
142.	KJV54535	49	5519.54	7.84	4470	13.06	Stable	115.31	0.167
143.	KJV54539	58	6600.03	9.41	2980	60.76	Unstable	141.38	0.69
144.	KJV54540	81	9259.63	5.41	4470	45.79	Unstable	101.11	-0.474
145.	KJV54555	53	6064.4	10.51	0	19.64	Stable	125.09	-0.323
146.	KJV54556	35	3975.67	4.95	0	71.87	Unstable	128	0.249
147.	KJV54581	56	6691.72	8.03	5960	26.94	Stable	95.71	-0.264
148.	KJV54582	180	19984.89	7.74	12740	45.77	Stable	93.17	-0.142
149.	KJV54587	180	20855.05	5.03	13410	36.48	Stable	81.22	-0.781
150.	KJV54614	494	47501.64	8.94	34060	49.43	Unstable	76.51	-0.838
151.	KJV54616	79	8928.51	9.02	8730	30.77	Stable	109.75	0.137
152.	KJV54666	464	54215.8	5.37	38655	41.06	Unstable	104.57	-0.314
153.	KJV54670	115	13224.05	6.56	12950	43.32	unstable	89.91	-0.313
154.	KJV54671	36	4464.4	9.06	6085	31.77	Stable	83.89	-0.308
155.	KJV54701	160	18135.32	7.7	8940	22.85	Stable	120	0.127

156.	KJV54705	32	3558.21	4.86	3558.21	35.63	Stable	136.88	0.078
157.	KJV54707	417	46281.55	642	51715	31.7	Stable	93.76	-0.158
158.	KJV54735	87	10282.72	9.43	7575	21.66	Stable	-0.522	79.54
159.	KJV54779	163	19304.35	9.7	1490	34.86	stable	93.31	-0.464
160.	KJV54783	255	27693.34	9.57	4595	49.56	Unstable	66.63	-1.001
161.	KJV54785	31	3609.28	8.1	1490	20.76	stable	122.58	-0.1
162.	KJV54789	265	29160.61	5.34	23880	52.14	Unstable	100.08	0.108
163.	KJV54829	56	6745.98	10.27	1490	43.82	Unstable	90.54	-1.041
164.	KJV54870	41	4701.52	4.35	1490	65.76	Unstable	130.73	0.515
165.	KJV54874	49	5558.52	5.97	9970	32.88	Stable	121.22	0.322
166.	KJV54877	47	5554.35	9.3	13980	67.26	Unstable	83.19	-0.838
167.	KJV54878	107	11976.65	9.91	15595	18.24	Stable	128.41	0.548
168.	KJV54879	107	12446.56	9.59	18910	23.3	Stable	108.32	-0.252
169.	KJV54906	226	25895.51	9.38	14565	33.22	Stable	114.25	0.083
170.	KJV54907	29	3277.82	3.49	0	42.2	Unstable	127.59	1.145
171.	KJV54908	30	3331.9	10	5500	-4.11	stable	71.33	-0.363
172.	KJV54909	40	4552.12	9.82	6990	15.85	Stable	61	-0.878
173.	KJV54913	44	5293.09	8.14	5960	24.48	Stable	110.68	0.043
174.	KJV54946	56	6866.1	10.75	2980	53.12	Unstable	92.32	-0.811
175.	KJV54971	113	12368.7	4.6	8480	33.41	stable	75.04	-0.608
176.	KJV54975	81	8979.45	6.03	8480	33.61	stable	97.41	-0.017
177.	KJV54977	33	3893.42	4.51	2980	62.7	Unstable	56.06	-0.588
178.	KJV54978	63	7435.84	9.47	10095	25.3	stable	111.43	0.279
179.	KJV55027	224	25720.98	9.55	25120	27.1	stable	139.24	0.877
180.	KJV55028	52	6266.35	9.3	10095	26.61	Stable	99.42	-0.56
181.	KJV55033	279	30141.23	5.5	13450	44.26	Unstable	88.49	-0.24
182.	KJV55034	123	14356.56	6.9	13410	33.13	Stable	108.54	-0.376
183.	KJV55035	36	4099.82	9.3	2980	46.42	Unstable	103.06	-0.392
184.	KJV55037	616	70199.99	5.85	81710	35.38	Stable	86.09	-0.387
185.	KJV55079	53	6273.46	9.47	4595	35.43	stable	95.66	-0.606
186.	KJV55080	47	5324.39	10.39	1490	70.93	Stable	114.04	-0.174
187.	KJV55165	142	16597.37	8.67	13535	43.68	Unstable	97.39	-0.135

188.	KJV55217	85	9787.73	9.6	9065	47.4	Unstble	115.76	0.329
189.	KJV55220	73	8602.12	8.85	9970	23.04	Stable	128.08	-0.077
190.	KJV55222	585	65971.02	7.19	31330	42.28	unstable	110.67	-0.194
191.	KJV55225	50	5758.63	5.18	2980	24.24	stable	84	-0.316
192.	KJV55228	356	40464.37	6.6	37485	16.77	Stable	87.78	-0.61
193.	KJV55230	50	5758.63	5.18	2980	24.24	Stable	84	-0.316
194.	KJV55231	356	40464.37	6.6	37485	16.77	Stable	87.78	-0.61
195.	KJV55284	48	5118.12	7.68	1615	46.81	Unstable	121.88	0.715
196.	KJV55290	70	8148	9.22	4470	31.01	Stable	175.43	0.89
197.	KJV55293	126	14672.31	9.37	28880	26.2	stable	115.32	0.387
198.	KJV55294	53	6662.93	10.01	10430	64.92	Unstable	108.68	-0.198
199.	KJV55300	35	4061.9	11.48	1490	26.68	Stable	111.43	-0.074
200.	KJV55334	31	3843.46	9.4	0	35.12	Stable	84.84	-1.048
201.	KJV55336	157	17482.53	9.64	2980	41.27	Unstable	113.76	0.029
202.	KJV55342	108	12249.23	4.9	7450	32.74	stable	123.61	0.166
203.	KJV55344	107	11806.53	10.23	15595	17.34	stable	122.99	0.508
204.	KJV55346	50	5760.78	9.82	11460	11.15	stable	105.2	-0.176
205.	KJV55409	76	8460.73	5.85	2980	31.8	stable	92.37	-0.187
206.	KJV55456	48	4226.08	9.6	2980	27.57	stable	107.37	0.358
207.	KJV55458	33	3735.3	4.08	2980	17.96	Stable	130	0.509
208.	KJV55462	66	7856.45	10.01	5960	45.73		128.48	0.152
209.	KJV55465	68	8073.55	10.52	9970	50.11	Unstable	136.18	0.187
210.	KJV55474	120	14438.55	9.52	36900	38.22	Stable	73.92	-0.69
211.	KJV55527	82	9631.11	6.69	11710	61.02	Unstable	109.27	-0.118
212.	KJV55533	249	28093.77	5.56	21890	24.7	Stable	93.33	-0.314
213.	KJV55535	830	93400.46	8.77	69360	29.38	stable	110.9	-0.03
214.	KJV55597	44	4881.91	9.9	0	41.45	Unstable	110.68	0.332
215.	KJV55599	379	44699.13	7.1	43110	42.25	Unstable	89.05	-0.682
216.	KJV55659	55	6321.65	8.99	4595	32.97	Stable	131.09	0.904
217.	KJV55666	601	70016.82	8.4	72450	46.4	Unstable	108.35	-0.158
218.	KJV55667	42	4755.78	10.52	1490	19.93	Stable	97.38	-0.581
219.	KJV55680	39	4629.53	10.42	4470	32.87	Stable	62.56	-1.177

220.	KJV55734	69	8249.6	9.76	7450	-6.63	Stable	78.84	-0.539
221.	KJV55744	412	45560.16	8.92	25565	55.59	Unstable	78.62	-0.576
222.	KJV55746	50	5717.97	9.46	1490	16.37	Stable	117	0.108
223.	KJV55751	829	96072.11	7.27	79650	37.42	Stable	81.97	-0.575
224.	KJV55806	65	7451.56	7.91	5960	32.72	Stable	90.15	0.471
225.	KJV55810	112	12874.17	10.11	18450	39.32	Stable	126.25	0.066
226.	KJV55821	72	8446.03	9.94	9970	36.79	Stable	87.92	-0.435
227.	KJV55871	248	29664.89	9.24	39435	32.58	Stable	118.75	0.114
228.	KJV55874	75	8911.67	10.09	14440	14.09	Stable	103.87	-0.304
229.	KJV55882	252	28091.68	5.23	20900	28.29	Stable	87.39	-0.232
230.	KJV55884	399	44086.61	5.64	50810	30.94	Stable	82.86	-0.294
231.	KJV55885	29	3482.22	9.82	5960	55.8	Unstable	70.69	-0.693
232.	KJV55951	188	22314.77	9.48	19035	49.4	Unstable	89.15	-0.568
233.	KJV55957	64	7892.61	9.35	10555	46.43	Unstable	97.34	0.091
234.	KJV55958	203	24205.7	9.75	20650	41.58	Unstable	85.02	-0.773
235.	KJV55962	231	26313.6	4.67	23420	51.6	Unstable	81.86	-0.437
236.	KJV56036	54	6382.63	9.67	9970	15.43	Stable	91.85	-0.294
237.	KJV56037	141	16380.73	8.83	21430	38.63	Stable	84.89	-0.323
238.	KJV56040	36	4411.14	8.74	8605	43.93	Unstable	83.89	-0.419
239.	KJV56045	86	10058.94	6.52	11585	28.31	Stable	104.3	0.535
240.	KJV56047	146	17402.65	10.36	10095	28.88	Stable	85.41	-0.91
241.	KJV56053	58	6347.07	4.43	2980	36.76	Stable	94.31	-0.26
242.	KJV56131	38	4509.3	8.98	1490	23	Stable	69.21	-0.479
243.	KJV56132	128	14428.84	9.78	6085	25.2	Stable	90	0.116
244.	KJV56137	44	17369.07	9.43	34380	47.81	Unstable	114.93	0.683
245.	KJV56142	52	6230.23	9.39	4595	88.08	Unstable	88.08	-0.158
246.	KJV56143	109	11043.43	7.98	1490	43.32	Unstable	80.64	0.168
247.	KJV56203	427	47746.98	7.16	52050	36.12	Stable	102.95	0.11
248.	KJV56204	65	7604.05	9.1	7575	21.95	Stable	112.46	0.349
249.	KJV56205	227	25842.54	9.74	26150	32.3	Stable	121.01	0.095
250.	KJV56209	174	20095.62	9.7	28420	35.39	Stable	102.64	0.033
251.	KJV56211	47	5116.31	5.79	125	42.6	Unstable	147.45	1.264

252.	KJV56214	129	14793.42	5.22	5960	48.39	Unstable	89.22	-0.633
253.	KJV56219	65	6997.39	4.68	5500	46.93	Unstable	133.54	1.049
254.	KJV56296	57	6751.79	7.9	8480	48.61	Unstable	66.49	-0.079
255.	KJV56300	85	10219.03	10.02	4595	16.76	stable	85.88	-0.578
256.	KJV56304	59	6531.43	9.9	6990	12.94	stable	72.88	-0.495
257.	KJV56305	236	27495.22	8.81	24995	57.54	Stable	80.21	-0.677
258.	KJV56385	97	11005.92	7.91	8940	37.37	stable	130.62	0.247
259.	KJV56401	147	17235.52	5.33	33920	49.31	Unstable	84.29	-0.402
260.	KJV56402	225	25618.38	7.69	20985	22.92	Stable	101.87	-0.168
261.	KJV56404	54	6328.7	8.53	6085	37.03	Stable	135.37	0.969
262.	KJV56408	436	50276.2	8.77	56520	39.58	Stable	99.54	-0.172
263.	KJV56474	38	4320.99	4.44	1490	17.84	stable	107.63	0.232
264.	KJV56480	41	4902.04	9.73	2980	6.3	Stable	128.29	0.227
265.	KJV56570	33	3779.49	8.8	1615	84.86	Unstable	109.39	-0.127
266.	KJV56573	659	75473.16	8.78	47010	44.95	Unstable	85.83	-0.502
267.	KJV56574	197	23332.65	9.11	17545	49.32	Unstable	83.55	-0.763
268.	KJV56575	1420	163618.36	5.3	113930	38.32	Stable	95.76	-0.299
269.	KJV56581	44	5118.96	7.84	125	83.91	Unstable	99.55	-0.1
270.	KJV56583	39	4166.47	5	4470	72.19	Unstable	27.69	-1.185
271.	KJV56669	251	27679.26	4.56	24785	41.63	Unstable	96.73	-0.008
272.	KJV56673	87	10311.22	9.94	16960	41.28	Unstable	106.44	-0.04
273.	KJV56675	96	11537.92	4.94	14440	61.06	Unstable	90.21	-0.697
274.	KJV56683	210	25024.43	9.48	29590	54.74	Unstable	110	-0.248
275.	KJV56684	251	28858.6	8.42	31080	43.6	Unstable	114.18	-0.035
276.	KJV56688	533	62253.35	8.43	60795	45.51	Unstable	116.55	0.068
277.	KJV56690	39	4511.25	6.11	0	24.01	Stable	127.44	-0.472
278.	KJV56780	54	6360.33	4.27	4470	53.51	Unstable	137.04	0.428
279.	KJV56783	37	4276.2	6.51	8480	27.01	Stable	147.3	0.832
280.	KJV56789	129	15104.63	9.82	14440	42.68	Unstable	102.79	-0.441
281.	KJV56930	350	39469.71	9.18	49195	33.03	Stable	159.11	1.08
282.	KJV56935	58	6819.89	10.2	4470	27.78	stable	99.14	-0.343
283.	KJV57117	356	40069.75	482	40145	31.02	stable	92.84	-0.355

284.	KJV57120	632	72691.95	6.34	96970	37.13	Stable	92.1	-0.338
285.	KJV57129	642	73523.59	8.19	90900	32.17	Stable	100.98	-0.285
286.	KJV57131	56	6016.8	8.59	3105	55.47	Unstable	59.29	-0.371
287.	KJV57139	616	69496.14	4.36	34505	35.4	Stable	75.23	-0.992
288.	KJV57144	38	4450.38	9.25	2980	35.81	Stable	110.53	-0.158
289.	KJV57200	187	21233.58	5.26	6990	56.07	Unstable	97.38	-0.83
290.	KJV57203	69	8026.86	10.1	11585	46.34	Unstable	138.55	0.7
291.	KJV57204	267	30453.84	7.52	21360	37.45	Stable	93.86	-0.157
292.	KJV57206	141	16544.96	6.82	15930	44.68	Unstable	108.58	-0.05
293.	KJV57207	62	6829.06	9.59	1615	45.7	Unstable	94.52	0.234
294.	KJV57212	50	5713.58	8.14	2980	13.38	Stable	99.4	0
295.	KJV57216	142	16869.63	9.62	17880	40.74	Unstable	110.49	-0.125
296.	KJV57217	200	23458.62	7.82	27850	26.22	Stable	90.65	-0.325
297.	KJV57219	496	57241.08	5.4	33390	43.19	unstable	98.51	-0.594
298.	KJV57225	231	25321.29	4.8	14900	35.61	Stable	84.11	-0.198
299.	KJV57230	181	21011.55	9.17	15025	51.29	Unstable	117.96	0.014
300.	KJV57297	323	35669.38	5.24	14690	44.93	Unstable	90.34	-0.337
301.	KJV57301	77	9047.97	10.03	9065	23.73	Stable	111.56	0.262
302.	KJV57311	85	10351.83	4.83	16055	48.4	Unstable	95.06	-0.176
303.	KJV57315	435	49889.33	4.54	34185	46.34	Unstable	89.63	-0.605
304.	KJV57318	46	5350.16	4.02	1490	76.97	unstable	133.48	-0.043
305.	KJV57330	65	7763.22	8.73	10430	32.54	Stable	138	0.366
306.	KJV57343	193	20718.79	5.42	4720	64.67	Unstable	53.63	-0.749
307.	KJV57346	195	22161.63	8.81	22710	30.27	Stable	98.05	-0.432
308.	KJV57347	95	11068.87	6.72	14440	31.89	Stable	114.95	-0.041
309.	KJV57348	240	27591.67	5.35	13410	34.8	Stable	81.75	-0.964
310.	KJV57353	341	38410.59	5.06	46340	49.37	Unstable	81.88	-0.526
311.	KJV57356	56	6313.38	9.36	2980	22.03	Stable	95.89	-0.14
312.	KJV57360	75	8562.49	10.46	5960	41.67	Unstable	116.93	0.107
313.	KJV57361	52	5867.64	6.7	6990	28.83	Stable	95.58	-0.352
314.	KJV57362	45	5537.27	9.63	2980	44.27	Unstable	45.56	-1.798
315.	KJV57365	112	13363.55	9.69	27055	38.2	Stable	106.25	-0.032

316.	KJV57366	34	3831.5	5.5	1490	1.92	Stable	122.94	0.359
317.	KJV57369	57	6882.15	9.52	5960	34.94	Stable	109.47	-0.004
318.	KJV57375	56	6549.71	8.85	1490	38.72	Stable	102.68	-0.225
319.	KJV57379	55	6739.95	9.2	6085	38.86	Stable	88.55	-0.129
320.	KJV57382	714	80160.83	9.49	37860	39.23	Stable	83.1	-0.635
321.	KJV57383	45	5376.28	10.09	4470	60.48	Unstable	84.44	-0.704
322.	KJV57385	49	5890.97	9.78	9970	4.61	Stable	77.55	-0.612
323.	KJV57393	492	56002.19	7.01	66490	18.65	Stable	100.47	-0.185
324.	KJV57403	595	66081.16	6.45	44280	35.91	Stable	93.48	-0.259
325.	KJV57410	34	3775.33	4.3	0	6.98	Stable	134.71	0.229
326.	KJV57412	88	10362.99	6.07	125	48.67	Unstable	122.84	-0.343
327.	KJV57416	43	4976.93	9.73	1490	16.45	Stable	88.6	-0.384
328.	KJV57418	460	40512.29	4.7	38125	40.69	Unstable	83.64	-0.262
329.	KJV57432	52	6234.3	8.85	2980	28.55	Stable	78.85	-0.121
330.	KJV57451	30	3668.37	9.3	8480	-21.28	Stable	78	-0.21
331.	KJV57461	55	6306.38	8.57	4595	82.96	Unstable	97.64	0.171
332.	KJV57462	32	3873.57	10.52	1490	64.51	Unstable	73.12	-0.9
333.	KJV57464	38	4513.31	7.79	1615	16.72	Stable	79.47	0.005
334.	KJV57571	559	68495.37	8.83	49320	45.55	Unstable	85.04	0.546
335.	KJV57572	31	3352.65	5.36	1490	16.47	Stable	43.87	-0.913
336.	KJV57574	255	27658.9	5.74	18450	48.66	Unstable	74.71	-0.687
337.	KJV57580	174	19184.36	9.02	13325	37.41	Stable	157.47	1.248
338.	KJV57582	73	8090.24	9.52	9970	33.43	Stable	80.14	-0.499
339.	KJV57585	447	52045.79	9.36	73480	47.85	Unstable	102.95	-0.185
340.	KJV57590	69	8495.96	9.37	17420	37.46	Stable	94.64	0.122
341.	KJV57610	32	3774.6	10	5960	34.39	Stable	82.19	-0.191
342.	KJV57616	50	5769.72	11.57	1615	42.74	Unstable	72	-0.842
343.	KJV57620	62	7411.44	5.19	9065	18.41	Stable	80.16	-0.424
344.	KJV57626	196	21769.15	6.68	15400	42.42	Unstable	132.91	0.912
