# Supplementary material for: In silico characterization of hypothetical proteins from Orientia tsutsugamushi str. Karp uncovers virulence genes
Source: Heliyon. 2019 Nov 1;5(10):e02734. doi: 10.1016/j.heliyon.2019.e02734 (PMC6838952; doi:10.1016/j.heliyon.2019.e02734)
Supplement: Supplementary file 2 [file mmc2.pdf]

S2_Table: List of predicted sub cellular localizations of 344 HPs from *Orientia tsutsugamushi str. Karp.*

S.NO	Accession No.	PSLpred Prediction	CELLO Prediction	Proteins with common predictions	Accession No.	PSLpred Prediction	CELLO Prediction
1.	KJV50500	Cytoplasmic	Cytoplasmic				
2.	KJV50518	Cytoplasmic	Cytoplasmic		KJV50500	Cytoplasmic	Cytoplasmic
3.	KJV50599	Inner-membrane	Cytoplasmic		KJV50518	Cytoplasmic	Cytoplasmic
4.	KJV50624	Cytoplasmic	Cytoplasmic		KJV50624	Cytoplasmic	Cytoplasmic
5.	KJV50625	Outer Membrane	Outer Membrane		KJV50625	Outer Membrane	Outer Membrane
6.	KJV50645	Cytoplasmic	Cytoplasmic		KJV50645	Cytoplasmic	Cytoplasmic
7.	KJV50671	Extracellular	Cytoplasmic		KJV50672	Cytoplasmic	Cytoplasmic
8.	KJV50672	Cytoplasmic	Cytoplasmic		KJV50818	Cytoplasmic	Cytoplasmic
9.	KJV50707	Cytoplasmic	Periplasmic		KJV50905	Cytoplasmic	Cytoplasmic
10.	KJV50735	Cytoplasmic	Extracellular		KJV50906	Cytoplasmic	Cytoplasmic
11.	KJV50787	Cytoplasmic	Extracellular		KJV50907	Cytoplasmic	Cytoplasmic
12.	KJV50815	Outer Membrane	Cytoplasmic		KJV50939	Cytoplasmic	Cytoplasmic
13.	KJV50818	Cytoplasmic	Cytoplasmic		KJV50940	Cytoplasmic	Cytoplasmic
14.	KJV50905	Cytoplasmic	Cytoplasmic		KJV50999	Cytoplasmic	Cytoplasmic
15.	KJV50906	Cytoplasmic	Cytoplasmic		KJV51000	Cytoplasmic	Cytoplasmic
16.	KJV50907	Cytoplasmic	Cytoplasmic		KJV51002	Cytoplasmic	Cytoplasmic
17.	KJV50939	Cytoplasmic	Cytoplasmic		KJV51003	Cytoplasmic	Cytoplasmic
18.	KJV50940	Cytoplasmic	Cytoplasmic		KJV51004	Cytoplasmic	Cytoplasmic
19.	KJV50970	Cytoplasmic	Inner-membrane		KJV51035	Cytoplasmic	Cytoplasmic
20.	KJV50994	Cytoplasmic	Extracellular		KJV51076	Cytoplasmic	Cytoplasmic
21.	KJV50999	Cytoplasmic	Cytoplasmic		KJV51080	Cytoplasmic	Cytoplasmic
22.	KJV51000	Cytoplasmic	Cytoplasmic		KJV51128	Periplasmic	Periplasmic
23.	KJV51002	Cytoplasmic	Cytoplasmic		KJV51131	Cytoplasmic	Cytoplasmic
24.	KJV51003	Cytoplasmic	Cytoplasmic		KJV51142	Cytoplasmic	Cytoplasmic
25.	KJV51004	Cytoplasmic	Cytoplasmic		KJV51205	Extracellular	Extracellular
26.	KJV51035	Cytoplasmic	Cytoplasmic		KJV51252	Cytoplasmic	Cytoplasmic
27.	KJV51076	Cytoplasmic	Cytoplasmic		KJV51253	Outer Membrane	Outer Membrane
28.	KJV51080	Cytoplasmic	Cytoplasmic		KJV51286	Cytoplasmic	Cytoplasmic
29.	KJV51128	Periplasmic	Periplasmic		KJV51289	Cytoplasmic	Cytoplasmic
30.	KJV51131	Cytoplasmic	Cytoplasmic		KJV51373	Cytoplasmic	Cytoplasmic
31.	KJV51134	Outer Membrane	Cytoplasmic		KJV51375	Cytoplasmic	Cytoplasmic
32.	KJV51142	Cytoplasmic	Cytoplasmic		KJV51376	Cytoplasmic	Cytoplasmic
33.	KJV51205	Extracellular	Extracellular		KJV51411	Cytoplasmic	Cytoplasmic
34.	KJV51252	Cytoplasmic	Cytoplasmic		KJV51578	Cytoplasmic	Cytoplasmic
35.	KJV51253	Outer Membrane	Outer Membrane		KJV51649	Cytoplasmic	Cytoplasmic
36.	KJV51286	Cytoplasmic	Cytoplasmic		KJV51694	Cytoplasmic	Cytoplasmic
37.	KJV51289	Cytoplasmic	Cytoplasmic		KJV51695	Cytoplasmic	Cytoplasmic
38.	KJV51373	Cytoplasmic	Cytoplasmic		KJV51776	Cytoplasmic	Cytoplasmic
39.	KJV51375	Cytoplasmic	Cytoplasmic		KJV51784	Cytoplasmic	Cytoplasmic
40.	KJV51376	Cytoplasmic	Cytoplasmic		KJV51788	Cytoplasmic	Cytoplasmic
41.	KJV51409	Outer Membrane	Cytoplasmic		KJV51829	Cytoplasmic	Cytoplasmic
42.	KJV51411	Cytoplasmic	Cytoplasmic		KJV51877	Cytoplasmic	Cytoplasmic
43.	KJV51578	Cytoplasmic	Cytoplasmic		KJV51880	Cytoplasmic	Cytoplasmic
44.	KJV51579	Outer Membrane	Cytoplasmic		KJV51881	Cytoplasmic	Cytoplasmic
45.	KJV51581	Inner-membrane	Cytoplasmic		KJV51919	Cytoplasmic	Cytoplasmic
46.	KJV51649	Cytoplasmic	Cytoplasmic		KJV52046	Outer Membrane	Outer Membrane
47.	KJV51694	Cytoplasmic	Cytoplasmic		KJV52143	Cytoplasmic	Cytoplasmic
48.	KJV51695	Cytoplasmic	Cytoplasmic		KJV52144,	Cytoplasmic	Cytoplasmic
49.	KJV51776	Cytoplasmic	Cytoplasmic		KJV52230	Cytoplasmic	Cytoplasmic
50.	KJV51784	Cytoplasmic	Cytoplasmic		KJV52234	Cytoplasmic	Cytoplasmic
51.	KJV51788	Cytoplasmic	Cytoplasmic		KJV52376	Cytoplasmic	Cytoplasmic
52.	KJV51829	Cytoplasmic	Cytoplasmic		KJV52428	Cytoplasmic	Cytoplasmic
53.	KJV51877	Cytoplasmic	Cytoplasmic		KJV52477	Cytoplasmic	Cytoplasmic
54.	KJV51880	Cytoplasmic	Cytoplasmic		KJV52478	Outer Membrane	Outer Membrane
55.	KJV51881	Cytoplasmic	Cytoplasmic		KJV52571	Cytoplasmic	Cytoplasmic
56.	KJV51919	Cytoplasmic	Cytoplasmic		KJV52622	Cytoplasmic	Cytoplasmic
57.	KJV51964	Cytoplasmic	Extra Cellular		KJV52681	Extracellular	Extracellular
58.	KJV52046	Outer Membrane	Outer Membrane		KJV52748	Cytoplasmic	Cytoplasmic
59.	KJV52048	Outer Membrane	Extracellular		KJV52749	Outer Membrane	Outer Membrane
60.	KJV52143	Cytoplasmic	Cytoplasmic		KJV52751	Outer Membrane	Outer Membrane
61.	KJV52144,	Cytoplasmic	Cytoplasmic		KJV52869	Cytoplasmic	Cytoplasmic
62.	KJV52230	Cytoplasmic	Cytoplasmic		KJV52928	Cytoplasmic	Cytoplasmic

63.	KJV52234	Cytoplasmic	Cytoplasmic		KJV53004	Cytoplasmic	Cytoplasmic
64.	KJV52319	Outer Membrane	Cytoplasmic		KJV53007	Outer Membrane	Outer Membrane
65.	KJV52373	Periplasmic	Cytoplasmic		KJV53066	Cytoplasmic	Cytoplasmic
66.	KJV52376	Cytoplasmic	Cytoplasmic		KJV53067	Cytoplasmic	Cytoplasmic
67.	KJV52426	Outer Membrane	Cytoplasmic		KJV53068	Cytoplasmic	Cytoplasmic
68.	KJV52428	Cytoplasmic	Cytoplasmic		KJV53073	Outer Membrane	Outer Membrane
69.	KJV52477	Cytoplasmic	Cytoplasmic		KJV53189	Cytoplasmic	Cytoplasmic
70.	KJV52478	Outer Membrane	Outer Membrane		KJV53192	Cytoplasmic	Cytoplasmic
71.	KJV52571	Cytoplasmic	Cytoplasmic		KJV53203	Cytoplasmic	Cytoplasmic
72.	KJV52622	Cytoplasmic	Cytoplasmic		KJV53284	Extracellular	Extracellular
73.	KJV52681	Extracellular	Extracellular		KJV53363	Cytoplasmic	Cytoplasmic
74.	KJV52748	Cytoplasmic	Cytoplasmic		KJV53524	Cytoplasmic	Cytoplasmic
75.	KJV52749	Outer Membrane	Outer Membrane		KJV53634	Extracellular	Extracellular
76.	KJV52750	Cytoplasmic	Cytoplasmic		KJV53715	Cytoplasmic	Cytoplasmic
77.	KJV52751	Outer Membrane	Outer Membrane		KJV53716	Cytoplasmic	Cytoplasmic
78.	KJV52864	Extracellular	Cytoplasmic		KJV53819	Cytoplasmic	Cytoplasmic
79.	KJV52869	Cytoplasmic	Cytoplasmic		KJV53820	Cytoplasmic	Cytoplasmic
80.	KJV52928	Cytoplasmic	Cytoplasmic		KJV53914	Cytoplasmic	Cytoplasmic
81.	KJV53004	Cytoplasmic	Cytoplasmic		KJV53916	Cytoplasmic	Cytoplasmic
82.	KJV53007	Outer Membrane	Outer Membrane		KJV53919	Cytoplasmic	Cytoplasmic
83.	KJV53065	Cytoplasmic	Extracellular		KJV53937	Cytoplasmic	Cytoplasmic
84.	KJV53066	Cytoplasmic	Cytoplasmic		KJV53939	Cytoplasmic	Cytoplasmic
85.	KJV53067	Cytoplasmic	Cytoplasmic		KJV54139	Cytoplasmic	Cytoplasmic
86.	KJV53068	Cytoplasmic	Cytoplasmic		KJV54140	Cytoplasmic	Cytoplasmic
87.	KJV53073	Outer Membrane	Outer Membrane		KJV54141	Cytoplasmic	Cytoplasmic
88.	KJV53125	Outer Membrane	Cytoplasmic		KJV54143	Cytoplasmic	Cytoplasmic
89.	KJV53129	Outer Membrane	Cytoplasmic		KJV54147	Cytoplasmic	Cytoplasmic
90.	KJV53187	Outer Membrane	Cytoplasmic		KJV54170	Cytoplasmic	Cytoplasmic
91.	KJV53188	Outer Membrane	Periplasmic		KJV54341	Cytoplasmic	Cytoplasmic
92.	KJV53189	Cytoplasmic	Cytoplasmic		KJV54343	Cytoplasmic	Cytoplasmic
93.	KJV53192	Cytoplasmic	Cytoplasmic		KJV54368	Cytoplasmic	Cytoplasmic
94.	KJV53203	Cytoplasmic	Cytoplasmic		KJV54398	Cytoplasmic	Cytoplasmic
95.	KJV53284	Extracellular	Extracellular		KJV54413	Cytoplasmic	Cytoplasmic
96.	KJV53363	Cytoplasmic	Cytoplasmic		KJV54459	Cytoplasmic	Cytoplasmic
97.	KJV53442	Inner-membrane	Cytoplasmic		KJV54463	Cytoplasmic	Cytoplasmic
98.	KJV53524	Cytoplasmic	Cytoplasmic		KJV54465	Cytoplasmic	Cytoplasmic
99.	KJV53634	Extracellular	Extracellular		KJV54466	Cytoplasmic	Cytoplasmic
100.	KJV53715	Cytoplasmic	Cytoplasmic		KJV54488	Cytoplasmic	Cytoplasmic
101.	KJV53716	Cytoplasmic	Cytoplasmic		KJV54489	OuterMembrane	OuterMembrane
102.	KJV53819	Cytoplasmic	Cytoplasmic		KJV54492	Cytoplasmic	Cytoplasmic
103.	KJV53820	Cytoplasmic	Cytoplasmic		KJV54506	Cytoplasmic	Cytoplasmic
104.	KJV53914	Cytoplasmic	Cytoplasmic		KJV54508	OuterMembrane	OuterMembrane
105.	KJV53916	Cytoplasmic	Cytoplasmic		KJV54527	Cytoplasmic	Cytoplasmic
106.	KJV53919	Cytoplasmic	Cytoplasmic		KJV54528	Cytoplasmic	Cytoplasmic
107.	KJV53935	Extracellular	Outer Membrane		KJV54529	Cytoplasmic	Cytoplasmic
108.	KJV53937	Cytoplasmic	Cytoplasmic		KJV54535	Cytoplasmic	Cytoplasmic
109.	KJV53939	Cytoplasmic	Cytoplasmic		KJV54539	Cytoplasmic	Cytoplasmic
110.	KJV54139	Cytoplasmic	Cytoplasmic		KJV54540	Cytoplasmic	Cytoplasmic
111.	KJV54140	Cytoplasmic	Cytoplasmic		KJV54555	Cytoplasmic	Cytoplasmic
112.	KJV54141	Cytoplasmic	Cytoplasmic		KJV54556	Cytoplasmic	Cytoplasmic
113.	KJV54143	Cytoplasmic	Cytoplasmic		KJV54581	Cytoplasmic	Cytoplasmic
114.	KJV54146	Extracellular	Cytoplasmic		KJV54582	Cytoplasmic	Cytoplasmic
115.	KJV54147	Cytoplasmic	Cytoplasmic		KJV54587	Cytoplasmic	Cytoplasmic
116.	KJV54167	Cytoplasmic	Periplasmic		KJV54666	Cytoplasmic	Cytoplasmic
117.	KJV54168	Outer membrane	Cytoplasmic		KJV54670	Cytoplasmic	Cytoplasmic
118.	KJV54170	Cytoplasmic	Cytoplasmic		KJV54705	Cytoplasmic	Cytoplasmic
119.	KJV54341	Cytoplasmic	Cytoplasmic		KJV54783	Cytoplasmic	Cytoplasmic
120.	KJV54343	Cytoplasmic	Cytoplasmic		KJV54829	OuterMembrane	OuterMembrane
121.	KJV54362	Extracellular	Cytoplasmic		KJV54870	Cytoplasmic	Cytoplasmic
122.	KJV54368	Cytoplasmic	Cytoplasmic		KJV54874	Cytoplasmic	Cytoplasmic
123.	KJV54370	Cytoplasmic	Periplasmic		KJV54907	Cytoplasmic	Cytoplasmic
124.	KJV54388	Outer membrane	Cytoplasmic		KJV54908	Cytoplasmic	Cytoplasmic
125.	KJV54398	Cytoplasmic	Cytoplasmic		KJV55028	Inner membrane	Inner membrane
126.	KJV54413	Cytoplasmic	Cytoplasmic		KJV55217	cytoplasmic	Cytopplasmic
127.	KJV54438	Cytoplasmic	Periplasmic		KJV55222	cytoplasmic	Cytopplasmic
128.	KJV54459	Cytoplasmic	Cytoplasmic		KJV55228	Periplasmic	Cytopplasmic
129.	KJV54463	Cytoplasmic	Cytoplasmic		KJV55230	cytoplasmic	Cytopplasmic

130.	KJV54464	Inner membrane	Cytoplasmic	
131.	KJV54465	Cytoplasmic	Cytoplasmic	
132.	KJV54466	Cytoplasmic	Cytoplasmic	
133.	KJV54488	Cytoplasmic	Cytoplasmic	
134.	KJV54489	OuterMembrane	OuterMembrane	
135.	KJV54492	Cytoplasmic	Cytoplasmic	
136.	KJV54506	Cytoplasmic	Cytoplasmic	
137.	KJV54508	OuterMembrane	OuterMembrane	
138.	KJV54527	Cytoplasmic	Cytoplasmic	
139.	KJV54528	Cytoplasmic	Cytoplasmic	
140.	KJV54529	Cytoplasmic	Cytoplasmic	
141.	KJV54530	Cytoplasmic	Inner membrane	
142.	KJV54535	Cytoplasmic	Cytoplasmic	
143.	KJV54539	Cytoplasmic	Cytoplasmic	
144.	KJV54540	Cytoplasmic	Cytoplasmic	
145.	KJV54555	Cytoplasmic	Cytoplasmic	
146.	KJV54556	Cytoplasmic	Cytoplasmic	
147.	KJV54581	Cytoplasmic	Cytoplasmic	
148.	KJV54582	Cytoplasmic	Cytoplasmic	
149.	KJV54587	Cytoplasmic	Cytoplasmic	
150.	KJV54614	Extra cellular	Cytoplasmic	
151.	KJV54616	Extra cellular	OuterMembrane	
152.	KJV54666	Cytoplasmic	Cytoplasmic	
153.	KJV54670	Cytoplasmic	Cytoplasmic	
154.	KJV54671	OuterMembrane	Cytoplasmic	
155.	KJV54701	OuterMembrane	Cytoplasmic	
156.	KJV54705	Cytoplasmic	Cytoplasmic	
157.	KJV54707	Periplasmic	Cytoplasmic	
158.	KJV54735	Extra cellular	Periplasmic	
159.	KJV54779	OuterMembrane	Extra cellular	
160.	KJV54783	Cytoplasmic	Cytoplasmic	
161.	KJV54785	Inner membrane	Periplasmic	
162.	KJV54789	OuterMembrane	Cytoplasmic	
163.	KJV54829	OuterMembrane	OuterMembrane	
164.	KJV54870	Cytoplasmic	Cytoplasmic	
165.	KJV54874	Cytoplasmic	Cytoplasmic	
166.	KJV54877	OuterMembrane	Cytoplasmic	
167.	KJV54878	OuterMembrane	Cytoplasmic	
168.	KJV54879	Inner membrane	Cytoplasmic	
169.	KJV54906	OuterMembrane	Cytoplasmic	
170.	KJV54907	Cytoplasmic	Cytoplasmic	
171.	KJV54908	Cytoplasmic	Cytoplasmic	
172.	KJV54909	OuterMembrane	Cytoplasmic	
173.	KJV54913	Extra cellular	Cytoplasmic	
174.	KJV54946	OuterMembrane	Cytoplasmic	
175.	KJV54971	OuterMembrane	Cytoplasmic	
176.	KJV54975	Extra cellular	Periplasmic	
177.	KJV54977	Periplasmic	Cytoplasmic	
178.	KJV54978	OuterMembrane	Cytoplasmic	
179.	KJV55027	OuterMembrane	Cytoplasmic	
180.	KJV55028	Inner membrane	Inner membrane	
181.	KJV55033	OuterMembrane	Cytoplasmic	
182.	KJV55034	OuterMembrane	Extra cellular	
183.	KJV55035	OuterMembrane	Cytoplasmic	
184.	KJV55037	OuterMembrane	cytoplasmic	
185.	KJV55079	OuterMembrane	Outer membrane	
186.	KJV55080	Periplasmic	OuterMembrane	
187.	KJV55165	OuterMembrane	Periplasmic	
188.	KJV55217	cytoplasmic	Cytopplasmic	
189.	KJV55220	Inner membrane	Cytopplasmic	
190.	KJV55222	cytoplasmic	Cytopplasmic	
191.	KJV55225	Outer membrane	Extra cellular	
192.	KJV55228	Periplasmic	Cytopplasmic	
193.	KJV55230	cytoplasmic	Cytopplasmic	
194.	KJV55231	cytoplasmic	Cytopplasmic	
195.	KJV55284	Inner membrane	Cytopplasmic	
196.	KJV55290	Outer membrane	InnerMembrane	

KJV55231	cytoplasmic	Cytopplasmic
KJV55300	Cytopplasmic	Cytopplasmic
KJV55334	Cytopplasmic	Cytopplasmic
KJV55336	Cytopplasmic	Cytopplasmic
KJV55342	Cytopplasmic	Cytopplasmic
KJV55456	Cytoplasmic	Cytopplasmic
KJV55527	Cytoplasmic	Cytopplasmic
KJV55533	Outer membrane	Outer membrane
KJV55659	Outer membrane	Outer membrane
KJV55666	Cytoplasmic	Cytoplasmic
KJV55667	Periplasmic	Periplasmic
KJV55744	Cytoplasmic	Cytoplasmic
KJV55746	Extra cellular	Extra cellular
KJV55751	Cytoplasmic	Cytoplasmic
KJV55821	Cytoplasmic	Cytoplasmic
KJV55951	Cytoplasmic	Cytoplasmic
KJV55958	Extra cellular	Extra cellular
KJV56036	Outer membrane	Outer membrane
KJV56037	Cytoplasmic	Cytoplasmic
KJV56045	Cytoplasmic	Cytoplasmic
KJV56132	Inner-membrane	InnerMembrane
KJV56142	Inner-membrane	InnerMembrane
KJV56143	Cytoplasmic	Cytoplasmic
KJV56205	Cytoplasmic	Cytoplasmic
KJV56209	Inner-membrane	InnerMembrane
KJV56304	OuterMembrane	OuterMembrane
KJV56385	Cytoplasmic	Cytoplasmic
KJV56401	Cytoplasmic	Cytoplasmic
KJV56404	Cytoplasmic	Cytoplasmic
KJV56474	Cytoplasmic	Cytoplasmic
KJV56570	OuterMembrane	OuterMembrane
KJV56574	Cytoplasmic	Cytoplasmic
KJV56675	Cytoplasmic	Cytoplasmic
KJV56780	Cytoplasmic	Cytoplasmic
KJV56789	Inner-membrane	Inner-membrane
KJV56935	Outer Membrane	OuterMembrane
KJV57117	Outer Membrane	OuterMembrane
KJV57120	Outer Membrane	OuterMembrane
KJV57131	Extra cellular	Extra cellular
KJV57206	Periplasmic	Periplasmic
KJV57212	Cytoplasmic	Cytoplasmic
KJV57219	Extra cellular	Extracellular
KJV57225	Cytoplasmic	Cytoplasmic
KJV57230	Extra cellular	Extracellular
KJV57315	Cytoplasmic	Cytoplasmic
KJV57318	Cytoplasmic	Cytoplasmic
KJV57330	Extra cellular	Extracellular
KJV57346	Cytoplasmic	Cytoplasmic
KJV57347	Extra cellular	Extracellular
KJV57356	Cytoplasmic	Cytoplasmic
KJV57365	Cytoplasmic	Cytoplasmic
KJV57379	Extracellular	Extracellular
KJV57403	Cytoplasmic	Cytoplasmic
KJV57451	Cytoplasmic	Cytoplasmic
KJV57461	Cytoplasmic	Cytoplasmic
KJV57464	OuterMembrane	OuterMembrane
KJV57572	Extra cellular	Extra cellular
KJV57574	Innermembrane	Innermembrane
KJV57582	Cytoplasmic	Cytoplasmic
KJV57585	Cytoplasmic	Cytoplasmic
KJV57620	Innermembrane	Innermembrane

197.	KJV55293	Inner membrane	Cytopplasmic	
198.	KJV55294	Outer membrane	Cytopplasmic	
199.	KJV55300	Cytopplasmic	Cytopplasmic	
200.	KJV55334	Cytopplasmic	Cytopplasmic	
201.	KJV55336	Cytopplasmic	Cytopplasmic	
202.	KJV55342	Cytopplasmic	Cytopplasmic	
203.	KJV55344	Outer membrane	Cytopplasmic	
204.	KJV55346	Periplasmic	Cytopplasmic	
205.	KJV55409	Extra cellular	Cytopplasmic	
206.	KJV55456	Cytoplasmic	Cytopplasmic	
207.	KJV55458	Outer membrane	Cytopplasmic	
208.	KJV55462	Outer membrane	Cytopplasmic	
209.	KJV55465	Outer membrane	Cytopplasmic	
210.	KJV55474	Outer membrane	Cytopplasmic	
211.	KJV55527	Cytoplasmic	Cytopplasmic	
212.	KJV55533	Outer membrane	Outer membrane	
213.	KJV55535	Outer membrane	Cytopplasmic	
214.	KJV55597	Periplasmic	Cytopplasmic	
215.	KJV55599	Outer membrane	Cytopplasmic	
216.	KJV55659	Outer membrane	Outer membrane	
217.	KJV55666	Cytoplasmic	Cytoplasmic	
218.	KJV55667	Periplasmic	Periplasmic	
219.	KJV55680	Outer membrane	Cytoplasmic	
220.	KJV55734	Periplasmic	Extra cellular	
221.	KJV55744	Cytoplasmic	Cytoplasmic	
222.	KJV55746	Extra cellular	Extra cellular	
223.	KJV55751	Cytoplasmic	Cytoplasmic	
224.	KJV55806	Outer membrane	Cytoplasmic	
225.	KJV55810	Inner membrane	Periplasmic	
226.	KJV55821	Cytoplasmic	Cytoplasmic	
227.	KJV55871	Outer membrane	Cytoplasmic	
228.	KJV55874	Periplasmic	Extra cellular	
229.	KJV55882	Periplasmic	Extra cellular	
230.	KJV55884	Outer membrane	Cytoplasmic	
231.	KJV55885	Outer membrane	Cytoplasmic	
232.	KJV55951	Cytoplasmic	Cytoplasmic	
233.	KJV55957	Extra cellular	Cytoplasmic	
234.	KJV55958	Extra cellular	Extra cellular	
235.	KJV55962	Outer membrane	Cytoplasmic	
236.	KJV56036	Outer membrane	Outer membrane	
237.	KJV56037	Cytoplasmic	Cytoplasmic	
238.	KJV56040	Inner-membrane	Cytoplasmic	
239.	KJV56045	Cytoplasmic	Cytoplasmic	
240.	KJV56047	Extra cellular	Cytoplasmic	
241.	KJV56053	OuterMembrane	Cytoplasmic	
242.	KJV56131	OuterMembrane	Cytoplasmic	
243.	KJV56132	Inner-membrane	InnerMembrane	
244.	KJV56137	OuterMembrane	Cytoplasmic	
245.	KJV56142	Inner-membrane	InnerMembrane	
246.	KJV56143	Cytoplasmic	Cytoplasmic	
247.	KJV56203	OuterMembrane	Extra cellular	
248.	KJV56204	Inner-membrane	Cytoplasmic	
249.	KJV56205	Cytoplasmic	Cytoplasmic	
250.	KJV56209	Inner-membrane	InnerMembrane	
251.	KJV56211	OuterMembrane	Cytoplasmic	
252.	KJV56214	Inner-membrane	Cytoplasmic	
253.	KJV56219	OuterMembrane	Cytoplasmic	
254.	KJV56296	OuterMembrane	Cytoplasmic	
255.	KJV56300	OuterMembrane	Cytoplasmic	
256.	KJV56304	OuterMembrane	OuterMembrane	
257.	KJV56305	OuterMembrane	Cytoplasmic	
258.	KJV56385	Cytoplasmic	Cytoplasmic	
259.	KJV56401	Cytoplasmic	Cytoplasmic	
260.	KJV56402	Inner-membrane	Cytoplasmic	
261.	KJV56404	Cytoplasmic	Cytoplasmic	
262.	KJV56408	OuterMembrane	Cytoplasmic	
263.	KJV56474	Cytoplasmic	Cytoplasmic	

264.	KJV56480	OuterMembrane	Cytoplasmic	
265.	KJV56570	OuterMembrane	OuterMembrane	
266.	KJV56573	Extra cellular	Cytoplasmic	
267.	KJV56574	Cytoplasmic	Cytoplasmic	
268.	KJV56575	Extra cellular	Cytoplasmic	
269.	KJV56581	Extra cellular	Periplasmic	
270.	KJV56583	Cytoplasmic	OuterMembrane	
271.	KJV56669	OuterMembrane	Cytoplasmic	
272.	KJV56673	Extra cellular	Cytoplasmic	
273.	KJV56675	Cytoplasmic	Cytoplasmic	
274.	KJV56683	Cytoplasmic	Inner-membrane	
275.	KJV56684	Cytoplasmic	OuterMembrane	
276.	KJV56688	OuterMembrane	Cytoplasmic	
277.	KJV56690	Inner-membrane	Cytoplasmic	
278.	KJV56780	Cytoplasmic	Cytoplasmic	
279.	KJV56783	OuterMembrane	Cytoplasmic	
280.	KJV56789	Inner-membrane	Inner-membrane	
281.	KJV56930	OuterMembrane	Cytoplasmic	
282.	KJV56935	OuterMembrane	OuterMembrane	
283.	KJV57117	OuterMembrane	OuterMembrane	
284.	KJV57120	OuterMembrane	OuterMembrane	
285.	KJV57129	Extra cellular	Cytoplasmic	
286.	KJV57131	Extra cellular	Extra cellular	
287.	KJV57139	Periplasmic	Cytoplasmic	
288.	KJV57144	Extra cellular	Cytoplasmic	
289.	KJV57200	Inner-membrane	Cytoplasmic	
290.	KJV57203	OuterMembrane	Cytoplasmic	
291.	KJV57204	OuterMembrane	Inner-membrane	
292.	KJV57206	Periplasmic	Periplasmic	
293.	KJV57207	OuterMembrane	Cytoplasmic	
294.	KJV57212	Cytoplasmic	Cytoplasmic	
295.	KJV57216	OuterMembrane	Cytoplasmic	
296.	KJV57217	Extra cellular	Cytoplasmic	
297.	KJV57219	Extra cellular	Extracellular	
298.	KJV57225	Cytoplasmic	Cytoplasmic	
299.	KJV57230	Extra cellular	Extracellular	
300.	KJV57297	Inner-membrane	Periplasmic	
301.	KJV57301	OuterMembrane	Cytoplasmic	
302.	KJV57311	Extra cellular	Extracellular	
303.	KJV57315	Cytoplasmic	Cytoplasmic	
304.	KJV57318	Cytoplasmic	Cytoplasmic	
305.	KJV57330	Extra cellular	Extracellular	
306.	KJV57343	Periplasmic	Cytoplasmic	
307.	KJV57346	Cytoplasmic	Cytoplasmic	
308.	KJV57347	Extra cellular	Extracellular	
309.	KJV57348	OuterMembrane	Extracellular	
310.	KJV57353	OuterMembrane	Cytoplasmic	
311.	KJV57356	Cytoplasmic	Cytoplasmic	
312.	KJV57360	OuterMembrane	Cytoplasmic	
313.	KJV57361	Inner-membrane	Cytoplasmic	
314.	KJV57362	Inner-membrane	Cytoplasmic	
315.	KJV57365	Cytoplasmic	Cytoplasmic	
316.	KJV57366	Inner-membrane	Cytoplasmic	
317.	KJV57369	OuterMembrane	Cytoplasmic	
318.	KJV57375	OuterMembrane	Cytoplasmic	
319.	KJV57379	Extracellular	Extracellular	
320.	KJV57382	Extra cellular	Cytoplasmic	
321.	KJV57383	Periplasmic	Cytoplasmic	
322.	KJV57385	Cytoplasmic	OuterMembrane	
323.	KJV57393	Extra cellular	OuterMembrane	
324.	KJV57403	Cytoplasmic	Cytoplasmic	
325.	KJV57410	Extra cellular	Cytoplasmic	
326.	KJV57412	Periplasmic	Cytoplasmic	
327.	KJV57416	Extracellular	OuterMembrane	
328.	KJV57418	OuterMembrane	Cytoplasmic	
329.	KJV57432	OuterMembrane	Cytoplasmic	
330.	KJV57451	Cytoplasmic	Cytoplasmic	

331.	KJV57461	Cytoplasmic	Cytoplasmic	
332.	KJV57462	Periplasmic	Cytoplasmic	
333.	KJV57464	OuterMembrane	OuterMembrane	
334.	KJV57571	Extra celllular	Cytoplasmic	
335.	KJV57572	Extra cellular	Extra cellular	
336.	KJV57574	Innermembrane	Innermembrane	
337.	KJV57580	Outer membrane	Cytoplasmic	
338.	KJV57582	Cytoplasmic	Cytoplasmic	
339.	KJV57585	Cytoplasmic	Cytoplasmic	
340.	KJV57590	Outer membrane	Cytoplasmic	
341.	KJV57610	innermembrane	Cytoplasmic	
342.	KJV57616	Outer membrane	Cytoplasmic	
343.	KJV57620	Innermembrane	Innermembrane	
344.	KJV57626	Outer membrane	Cytoplasmic	

Total	Innermembrane—28	Innermembrane—12
	Outer membrane—98	Outer membrane—29
	Cytoplasmic---161	Cytoplasmic---260
	Extra cellular---39	Extra cellular---28
	Periplasmic---18	Periplasmic---15
