# Supplementary material for: In silico characterization of hypothetical proteins from Orientia tsutsugamushi str. Karp uncovers virulence genes
Source: Heliyon. 2019 Nov 1;5(10):e02734. doi: 10.1016/j.heliyon.2019.e02734 (PMC6838952; doi:10.1016/j.heliyon.2019.e02734)
Supplement: Supplementary file 3 [file mmc3.pdf]

**S3_Table:** List of predicted functional Domain/motif by ScanProsite, Smart, Motif Scan, PFP-FunDSeqE for 344 HPs from *Orientia tsutsugamushi str. Karp.*

| S.no | Accession No. | ScanProsite | Smart | Motif Scan | PFP-FunDSeqE |
|---|---|---|---|---|---|
| 1. | KJV50500 | No hit | None | 1. CK2_PHOSPHO_SITE (16-19)<br>2. MYRISTYL (12-17)<br>3. PKC_PHOSPHO_SITE (16-18) | 4-helical up-and-down bundle |
| 2. | KJV50518 | No hit | DNA binding protein, | 1. AMIDATION (78-81)<br>2. CK2_PHOSPHO_SITE (28-31)<br>3. MYRISTYL (6-11)) | Immunoglobulin-like |
| 3. | KJV50599 | No hit | Chromodomain-Helicase-DNA-binding protein 7 | 1.MYRISTYL (62-67)<br>2.UPF0546(49-67 | DNA-binding 3-helical bundle |
| 4. | KJV50624 | No hit | No hit | 1.ASN_GLYCOSYLATION(65-68)<br>2.Bacterial Ig-like domain2(1-8) | Immunoglobulin-like |
| 5. | KJV50625 | PTR2 family proton/oligo peptide symporters | No hit | 1.ASN_GLYCOSYLATION (105-108)<br>2.CK2_PHOSPHO_SITE (63-66)<br>3.MYRISTYL (207-212)<br>4.PKC_PHOSPHO_SITE (23-30)<br>5.NUMOD3(120-133) | OB-fold |
| 6. | KJV50645 | No hit | phage integrase family protein | 1.ASN_GLYCOSYLATION(20-23)<br>2.CK2_PHOSPHO_SITE(65-68)<br>3.PKC_PHOSPHO_SITE(70-72)<br>4.PTR2_2(45-57) | 4-helical cytokines |
| 7. | KJV50671 | No hit | EST/SMG like protein | 1.ASN_GLYCOSYLATION (27-30)<br>2.CK2_PHOSPHO_SITE (54-57)<br>3.MYRISTYL (84-89)<br>4.PKC_PHOSPHO_SITE (93-95)<br>5.Bacterial Ig-like domain2 (1-2)<br>6.ZF_THAP (100-189) | 4-helical cytokines |
| 8. | KJV50672 | No hit | No hit | 1.ASN_GLYCOSYLATION (113-116)<br>2.CK2_PHOSPHO_SITE(67-69)<br>3.MYRISTYL(82-87)<br>4.PKC_PHOSPHO_SITE(68-70)<br>5.BIG1(1-3)<br>6.PXA(1-15) | OB-fold |
| 9. | KJV50707 | No hit | No hit | 1.ASN_GLYCOSYLATION(112-115)<br>2.CK2_PHOSPHO_SITE(71-73)<br>3.MYRISTYL(86-89)<br>4.PKC_PHOSPHO_SITE(63-66)<br>5.BIG1(1-3)<br>6.PXA(1-15) | OB-fold |
| 10. | KJV50735 | No hit | Magnesium Transporter | 1.ASN_GLYCOSYLATION<br>2.CAMP_PHOSPHO_SITE<br>3.CK2_PHOSPHO_SITE<br>4.MYRISTYL<br>5.PKC_PHOSPHO_SITE<br>6.Pumilio RNA-binding repeat profile<br>7.Domain of unknown function | immunoglobulin-like |
| 11. | KJV50787 | No hit | Transmembrane region | 1.ASN_GLYCOSYLATION<br>2.CAMP_PHOSPHO_SITE<br>3.CK2_PHOSPHO_SITE<br>4.MYRISTYL<br>5.PKC_PHOSPHO_SITE<br>6.Serine-rich region<br>7.Threonine-rich region<br>8.Fibritin_C<br>Fibritin C-terminal region<br>9.H2-forming N5,N10-methylenetetrahydromethanopterin dehydrogenase | Immunoglobulin-like |
| 12. | KJV50815 | No hit | PA14 domain-containing protein | 1.ASN_GLYCOSYLATION<br>2.CAMP_PHOSPHO_SITE<br>3.CK2_PHOSPHO_SITE<br>4.PKC_PHOSPHO_SITE<br>5.TYR_PHOSPHO_SITE<br>6. BRCA2 repeat | 4-helical cytokines |

| | | | | | |
|---|---|---|---|---|---|
| 13. | KJV50818 | No hit | No hit | 1.AMIDATION ASN_GLYCOSYLATION<br>2.CK2_PHOSPHO_SITE<br>3.PKC_PHOSPHO_SITE<br>4.TYR_PHOSPHO_SITE | 4-helical cytokines |
| 14. | KJV50905 | No hit | No hit | 1.ASN_GLYCOSYLATION<br>2.MYRISTYL<br>3.PKC_PHOSPHO_SITE<br>4.bacterial Ig-like domain 1 | Immunoglobulin-like |
| 15. | KJV50906 | No hit | replicative DNA helicase domain protein | 1.ASN_GLYCOSYLATION<br>2.PKC_PHOSPHO_SITE<br>3.PKC_PHOSPHO_SITE | DNA-binding 3-helical bundle |
| 16. | KJV50907 | No hit | No hit | 1.CAMP_PHOSPHO_SITE<br>2.PKC_PHOSPHO_SITE | DNA-binding 3-helical bundle |
| 17. | KJV50939 | No hit | AAA domain protein | 1.AMIDATION<br>2.ASN_GLYCOSYLATION<br>3.ASN_GLYCOSYLATION<br>4.CAMP_PHOSPHO_SITE | immunoglobulin-like |
| 18. | KJV50940 | No hit | No hit | 1.CK2_PHOSPHO_SITE<br>2.MYRISTYL | OB-fold |
| 19. | KJV50970 | No hit | Transmembrane | 1.ASN_GLYCOSYLATION<br>2.CAMP_PHOSPHO_SITE<br>3.CK2_PHOSPHO_SITE<br>4.PKC_PHOSPHO_SITE | 4-helical cytokines |
| 20. | KJV50994 | No hit | No hit | 1.ASN_GLYCOSYLATION<br>2.CK2_PHOSPHO_SITE<br>3.PKC_PHOSPHO_SITE<br>4.TYR_PHOSPHO_SITE | 4-helical cytokines |
| 21. | KJV50999 | No hit | No hit | 1.ASN_GLYCOSYLATION<br>2.CK2_PHOSPHO_SITE<br>3.MYRISTYL<br>4.PKC_PHOSPHO_SITE | OB-fold |
| 22. | KJV51000 | No hit | peptide ABC transporter substrate-binding protein | 1.AMIDATION<br>2.ASN_GLYCOSYLATION<br>3.CAMP_PHOSPHO_SITE<br>4.CK2_PHOSPHO_SITE<br>5.MYRISTYL<br>6.PKC_PHOSPHO_SITE | immunoglobulin-like |
| 23. | KJV51002 | No hit | No hit | 1.ASN_GLYCOSYLATION<br>2.MYRISTYL<br>3.PKC_PHOSPHO_SITE<br>4.PKC_PHOSPHO_SITE | OB-fold |
| 24. | KJV51003 | No hit | Transmembrane | 1.ASN_GLYCOSYLATION<br>2.CAMP_PHOSPHO_SITE<br>3.PKC_PHOSPHO_SITE<br>4.BIG1 | immunoglobulin-like |
| 25. | KJV51004 | No hit | magnesium/cobalt efflux protein | empty | 4-helical cytokines |
| 26. | KJV51035 | No hit | No hit | 1.ASN_GLYCOSYLATION<br>2.MYRISTYL | cytochrome c |
| 27. | KJV51076 | No hit | Transmembrane | 1.ASN_GLYCOSYLATION<br>2.PKC_PHOSPHO_SITE<br>3.BIG1<br>4.CW_binding_2 | DNA-binding 3-helical bundle |
| 28. | KJV51080 | No hit | No hit | 1.MYRISTYL | 4-helical cytokines |
| 29. | KJV51128 | No hit | No hit | 1.AMIDATION<br>2.ASN_GLYCOSYLATION<br>3.CAMP_PHOSPHO_SITE<br>4.CK2_PHOSPHO_SITE<br>5.MYRISTYL<br>6.PKC_PHOSPHO_SITE<br>7.TYR_PHOSPHO_SITE<br>8.Pepsin-I3 | 4-helical cytokines |
| 30. | KJV51131 | No hit | No hit | 1.ASN_GLYCOSYLATION<br>2.CK2_PHOSPHO_SITE<br>3.MYRISTYL<br>4.PKC_PHOSPHO_SITE<br>5.TYR_PHOSPHO_SITE | 4-helical cytokines |
| 31. | KJV51134 | No hit | dnaA N-terminal domain protein | 1.CK2_PHOSPHO_SITE<br>2.MYRISTYL<br>3.PKC_PHOSPHO_SITE | 4-helical cytokines |
| 32. | KJV51142 | No hit | | 1.ASN_GLYCOSYLATION<br>2.CAMP_PHOSPHO_SITE<br>3.CK2_PHOSPHO_SITE | Belta-grasp |

| | | | | | |
|---|---|---|---|---|---|
| | | | | 4.MYRISTYL<br>5.PKC_PHOSPHO_SITE<br>6.PFTA<br>7.Hexapep | |
| 33. | KJV51205 | No hit | Transmembrane | 1.ASN_GLYCOSYLATION<br>2.CK2_PHOSPHO_SITE<br>3.MYRISTYL<br>4.PKC_PHOSPHO_SITE<br>5.DUF308 | Cupredoxins |
| 34. | KJV51252 | No hit | Transposase, YhgA-like family protein | 1.CK2_PHOSPHO_SITE<br>2.MYRISTYL<br>3.PKC_PHOSPHO_SITE | OB-fold |
| 35. | KJV51253 | No hit | No hit | 1.ASN_GLYCOSYLATION<br>2.CAMP_PHOSPHO_SITE<br>3.CK2_PHOSPHO_SITE<br>4.MYRISTYL<br>5.PKC_PHOSPHO_SITE | OB-fold |
| 36. | KJV51286 | No hit | No hit | 1.PKC_PHOSPHO_SITE | DNA-binding 3-helical bundle |
| 37. | KJV51289 | No hit | No hit | 1.CK2_PHOSPHO_SITE<br>2. PKC_PHOSPHO_SITE | Belta-grasp |
| 38. | KJV51373 | No hit | secA DEAD-like domain protein | 1.PKC_PHOSPHO_SITE<br>2.TYR_PHOSPHO_SITE | EF-hand |
| 39. | KJV51375 | No hit | No hit | 1.ASN_GLYCOSYLATION<br>2.MYRISTYL | immunoglobulin-like |
| 40. | KJV51376 | No hit | No hit | 1.CK2_PHOSPHO_SITE<br>2.PKC_PHOSPHO_SITE<br>3.TYR_PHOSPHO_SITE | thioredoxin-like |
| 41. | KJV51409 | No hit | No hit | 1.ASN_GLYCOSYLATION<br>2.CAMP_PHOSPHO_SITE<br>3.CK2_PHOSPHO_SITE<br>4.MYRISTYL<br>5.PKC_PHOSPHO_SITE<br>6.TYR_PHOSPHO_SITE<br>7.DNA_pol3_theta | immunoglobulin-like |
| 42. | KJV51411 | No hit | No hit | 1.AMIDATION<br>2.CAMP_PHOSPHO_SITE<br>3.CK2_PHOSPHO_SITE<br>4.MYRISTYL<br>5.SRR<br>6.UPF0546 | 4-helical cytokines |
| 43. | KJV51578 | No hit | No hit | 1.AMIDATION<br>2.CAMP_PHOSPHO_SITE<br>3.CK2_PHOSPHO_SITE<br>4.MYRISTYL<br>5.SRR<br>6.UPF0546 | immunoglobulin-like |
| 44. | KJV51579 | No hit | dnaA N-terminal domain protein | 1.ASN_GLYCOSYLATION<br>2.PKC_PHOSPHO_SITE<br>3.ASN_RICH<br>4.DUF270<br>5.SKN1<br>6.NUMOD3 | 4-helical cytokines |
| 45. | KJV51581 | No hit | No hit | 1.AMIDATION<br>2.CAMP_PHOSPHO_SITE<br>3.CK2_PHOSPHO_SITE<br>4.MYRISTYL<br>5.SRR<br>6.UPF0546 | DNA-binding 3-helical bundle |
| 46. | KJV51649 | No hit | rhodanese-like domain protein | 1.AMIDATION<br>2.ASN_GLYCOSYLATION<br>3.CK2_PHOSPHO_SITE<br>4.MYRISTYL<br>5.PKC_PHOSPHO_SITE<br>6.GLPE_MF_01009<br>7.YCEA_MF_00469<br>8.RHODANESE_3<br>9.AFP<br>10.Rhodanese | (TIM)-barrel |
| 47. | KJV51694 | No hit | No hit | 1.AMIDATION<br>2.CK2_PHOSPHO_SITE<br>3.MYRISTYL<br>4.PKC_PHOSPHO_SITE<br>5.TYR_PHOSPHO_SITE | thioredoxin-like |

| | | | | | |
|---|---|---|---|---|---|
| 48. | KJV51695 | No hit | No hit | 1.ASN_GLYCOSYLATION<br>2.MYRISTYL | Immunoglobulin-like |
| 49. | KJV51776 | No hit | No hit | 1.ASN_GLYCOSYLATION<br>2.MYRISTYL<br>3.PKC_PHOSPHO_SITE<br>4.BIG1 | DNA-binding 3-helical bundle |
| 50. | KJV51784 | No hit | No hit | 1.ASN_GLYCOSYLATION<br>2.MYRISTYL<br>3.PKC_PHOSPHO_SITE | DNA-binding 3-helical bundle |
| 51. | KJV51788 | No hit | No hit | 1.ASN_GLYCOSYLATION<br>2.CK2_PHOSPHO_SITE<br>3.PKC_PHOSPHO_SITE | DNA-binding 3-helical bundle |
| 52. | KJV51829 | No hit | No hit | 1.MYRISTYL<br>2.PKC_PHOSPHO_SITE | DNA-binding 3-helical bundle |
| 53. | KJV51877 | No hit | No hit | 1.PKC_PHOSPHO_SITE | immunoglobulin-like |
| 54. | KJV51880 | No hit | Leucine rich repeat-containing protein | 1.ASN_GLYCOSYLATION<br>2.CK2_PHOSPHO_SITE<br>3.BIG1<br>4.NHL | DNA-binding 3-helical bundle |
| 55. | KJV51881 | No hit | No hit | 1.MYRISTYL<br>2.PKC_PHOSPHO_SITE<br>3.NLS_BP | immunoglobulin-like |
| 56. | KJV51919 | No hit | ATP-binding protein | 1.PKC_PHOSPHO_SITE | 4-helical cytokines |
| 57. | KJV51964 | No hit | conjugal transfer protein TraN | 1.ASN_GLYCOSYLATION<br>2.CK2_PHOSPHO_SITE<br>3.MYRISTYL | 4-helical cytokines |
| 58. | KJV52046 | No hit | Transmembrane | 1.ASN_GLYCOSYLATION<br>2.CK2_PHOSPHO_SITE<br>3.PKC_PHOSPHO_SITE<br>4.TYR_PHOSPHO_SITE<br>5.RAS | 4-helical cytokines |
| 59. | KJV52048 | No hit | No hit | 1.ASN_GLYCOSYLATION<br>2.CAMP_PHOSPHO_SITE<br>3.PKC_PHOSPHO_SITE<br>4.ASN_RICH<br>5.NUMOD3 | Belta-grasp |
| 60. | KJV52143 | No hit | **No hit** | 1. CK2_PHOSPHO_SITE<br>2. PKC_PHOSPHO_SITE | 4-helical cytokines |
| 61. | KJV52144, | No hit | **PA14 domain-containing protein** | 1.CAMP_PHOSPHO_SITE | **immunoglobulin-like** |
| 62. | KJV52230 | No hit | **alpha-amylase** | 1.ASN_GLYCOSYLATION<br>2.PKC_PHOSPHO_SITE | DNA-binding 3-helical bundle |
| 63. | KJV52234 | No hit | helix-turn-helix domain-containing protein | 1.ASN_GLYCOSYLATION<br>2.CK2_PHOSPHO_SITE<br>3.MYRISTYL]<br>4.PKC_PHOSPHO_SITE<br>5.pfam_fs:DRTGG<br>6.pfam_ls:LSPR | OB-fold |
| 64. | KJV52319 | No hit | **No hit** | 1.ASN_GLYCOSYLATION<br>2.CK2_PHOSPHO_SITE<br>3.MYRISTYL<br>4.PKC_PHOSPHO_SITE | OB-fold |
| 65. | KJV52373 | No hit | **No hit** | 1.prf:BIG1 | DNA-binding 3-helical bundle |
| 66. | KJV52376 | No hit | **DUF4956 domain-containing protein** | 1.ASN_GLYCOSYLATION<br>2.CK2_PHOSPHO_SITE<br>3.MYRISTYL<br>4.PKC_PHOSPHO_SITE<br>5.TYR_PHOSPHO_SITE<br>6.PDZ | 4-helical cytokines |
| 67. | KJV52426 | No hiit | **dnaA N-terminal domain protein** | 1.ASN_GLYCOSYLATION<br>2.CAMP_PHOSPHO_SITE<br>3.PKC_PHOSPHO_SITE<br>4.NUMOD3 | OB-fold |
| 68. | KJV52428 | No hit | **No hit** | 1.PKC_PHOSPHO_SITE | DNA-binding 3-helical bundle |
| 69. | KJV52477 | No hit | **GGDEF domain-containing protein** | 1.ASN_GLYCOSYLATION<br>2.CK2_PHOSPHO_SITE<br>3.PKC_PHOSPHO_SITE<br>4.PFTA | 4-helical cytokines |

| | | | | | |
|---|---|---|---|---|---|
| 70. | KJV52478 | No hit | **No hit** | 1.ASN_GLYCOSYLATION<br>2.CK2_PHOSPHO_SITE<br>3.MYRISTYL<br>4.PKC_PHOSPHO_SITE<br>5.TYR_PHOSPHO_SITE | 4-helical cytokines |
| 71. | KJV52571 | No hit | **No hit** | empty | DNA-binding 3-helical bundle |
| 72. | KJV52622 | No hit | **No hit** | 1.CK2_PHOSPHO_SITE | 4-helical cytokines |
| 73. | KJV52681 | No hit | **THAP domain-containing protein 9** | 1.ASN_GLYCOSYLATION<br>2.CAMP_PHOSPHO_SITE<br>3.CK2_PHOSPHO_SITE<br>4.PKC_PHOSPHO_SITE<br>5.TYR_PHOSPHO_SITE<br>6.ASN_RICH | 4-helical cytokines |
| 74. | KJV52748 | No hit | **No hit** | | **DNA-binding 3-helical bundle** |
| 75. | KJV52749 | No hit | **ABC transporter domain-containing protein** | 1.ASN_GLYCOSYLATION MYRISTYL<br>2.PKC_PHOSPHO_SITE<br>3.pfam_fs:KID | OB-fold |
| 76. | KJV52750 | No hit | **No hit** | 1.CK2_PHOSPHO_SITE<br>2.PKC_PHOSPHO_SITE<br>3.BIG1<br>4.DUF1626 | DNA-binding 3-helical bundle |
| 77. | KJV52751 | No hit | **T9SS C-terminal target domain-containing protein** | 1.ASN_GLYCOSYLATION<br>2.CK2_PHOSPHO_SITE<br>3.freq_pat:MICROBODIES_CTER<br>4.MYRISTYL<br>5.PKC_PHOSPHO_SITE<br>6.TYR_PHOSPHO_SITE<br>7.SRR | OB-fold |
| 78. | KJV52864 | No hit | **No hit** | 1.ASN_GLYCOSYLATION<br>2.CK2_PHOSPHO_SITE<br>3.CK2_PHOSPHO_SITE<br>4.MYRISTYL<br>5.PKC_PHOSPHO_SITE | immunoglobulin-lik |
| 79. | KJV52869 | No hit | **No hit** | | 4-helical cytokines |
| 80. | KJV52928 | No hit | **amino acid adenylation domain-containing protein** | 1.ASN_GLYCOSYLATION<br>2.CK2_PHOSPHO_SITE<br>3.PKC_PHOSPHO_SITE<br>4.ASN_RICH<br>5.NUMOD3 | DNA-binding 3-helical bundle |
| 81. | KJV53004 | No hit | **No hit** | 1.PKC_PHOSPHO_SITE<br>2.TYR_PHOSPHO_SITE | DNA-binding 3-helical bundle |
| 82. | KJV53007 | No hit | **No hit** | 1.ASN_GLYCOSYLATION<br>2.CK2_PHOSPHO_SITE<br>3.MYRISTYL<br>4.PKC_PHOSPHO_SITE<br>5.Hexapep | immunoglobulin-like |
| 83. | KJV53065 | No hit | **No hit** | 1.ASN_GLYCOSYLATION<br>2.MYRISTYL | Cupredoxins |
| 84. | KJV53066 | No hit | **No hit** | 1.ASN_GLYCOSYLATION<br>2.CAMP_PHOSPHO_SITE<br>3.CK2_PHOSPHO_SITE<br>4.MYRISTYL [?]<br>5.PKC_PHOSPHO_SITE<br>6.TYR_PHOSPHO_SITE<br>7.DUF91<br>8.Transposase_31 | OB-fold |
| 85. | KJV53067 | No hit | **PA14 domain-containing protein** | 1.ASN_GLYCOSYLATION<br>2.CK2_PHOSPHO_SITE<br>3.MYRISTYL<br>4.PKC_PHOSPHO_SITE<br>5.BIG1<br>6.LDLRB | immunoglobulin-like |
| 86. | KJV53068 | No hit | **T9SS C-terminal target domain-containing protein** | 1.AMIDATION<br>2.ASN_GLYCOSYLATION<br>3.CK2_PHOSPHO_SITE<br>4.MYRISTYL [?]<br>5.PKC_PHOSPHO_SITE<br>6.SlyX | OB-fold |

| | | | | | |
|---|---|---|---|---|---|
| 87. | KJV53073 | No hit | **No hit** | 1.ASN_GLYCOSYLATION<br>2.CAMP_PHOSPHO_SITE<br>3.CK2_PHOSPHO_SITE<br>4.PKC_PHOSPHO_SITE | 4-helical cytokines |
| 88. | KJV53125 | No hit | **dnaA N-terminal domain protein** | 1.ASN_GLYCOSYLATION<br>2.CAMP_PHOSPHO_SITE<br>3.PKC_PHOSPHO_SITE<br>4.NUMOD3 | OB-fold |
| 89. | KJV53129 | No hit | **No hit** | 1.ASN_GLYCOSYLATION<br>2.CK2_PHOSPHO_SITE<br>3.MYRISTYL<br>4.PKC_PHOSPHO_SITE<br>5.KID | OB-fold |
| 90. | KJV53187 | No hit | **AAA domain protein** | 1.ASN_GLYCOSYLATION<br>2.CAMP_PHOSPHO_SITE<br>3.PKC_PHOSPHO_SITE<br>4.Hexapep<br>5.Hexapep<br>6.NUMOD3 | OB-fold |
| 91. | KJV53188 | No hit | **outer membrane autotransporter barrel domain-containing protein** | 1.ASN_GLYCOSYLATION<br>2.CK2_PHOSPHO_SITE<br>3.MYRISTYL [?]<br>4.PKC_PHOSPHO_SITE<br>5.TYR_PHOSPHO_SITE | OB-fold |
| 92. | KJV53189 | No hit | **No hit** | 1.CAMP_PHOSPHO_SITE<br>2.NEAT | OB-fold |
| 93. | KJV53192 | No hit | **transposase domain protein** | 1.CAMP_PHOSPHO_SITE<br>2.CK2_PHOSPHO_SITE<br>3.MYRISTYL<br>4.PKC_PHOSPHO_SITE<br>5.NLS_BP | 4-helical cytokines |
| 94. | KJV53203 | No hit | **No hit** | 1.CK2_PHOSPHO_SITE<br>2.MYRISTYL<br>3.PKC_PHOSPHO_SITE | ferredoxin-like |
| 95. | KJV53284 | No hit | **CarboxypepD_reg-like domain-containing protein** | 1.ASN_GLYCOSYLATION<br>2.CK2_PHOSPHO_SITE<br>3.MYRISTYL<br>4.ASN_RICH | beta-trefoil |
| 96. | KJV53363 | No hit | **No hit** | 1.CK2_PHOSPHO_SITE | 4-helical cytokines |
| 97. | KJV53442 | No hit | **No hit** | 1.AMIDATION<br>2.CAMP_PHOSPHO_SITE<br>3.CK2_PHOSPHO_SITE<br>4.MYRISTYL<br>5.SRR<br>6.UPF0546 | DNA-binding 3-helical bundle |
| 98. | KJV53524 | No hit | **transmembrane** | 1.ASN_GLYCOSYLATION<br>2.PKC_PHOSPHO_SITE<br>3.BIG1<br>4.CW_binding_2 | 4-helical cytokines |
| 99. | KJV53634 | No hit | **PAS domain-containing sensor histidine kinase** | 1.ASN_GLYCOSYLATION<br>2.CK2_PHOSPHO_SITE<br>3.MYRISTYL<br>4.PKC_PHOSPHO_SITE<br>5.YBAB_MF_00274<br>6.FRATAXIN_2<br>7.HTH_LYTTR<br>8.Borrelia_rep<br>9.DUF149 | EF-hand |
| 100. | KJV53715 | No hit | **No ht** | Empty | 4-helical cytokines |
| 101. | KJV53716 | No hit | EST/SMG like protein | 1. ASN_GLYCOSYLATION<br>2. CK2_PHOSPHO_SITE<br>3. MYRISTYL<br>4. PKC_PHOSPHO_SITE<br>5. Bacterial Ig-like domain2 (<br>6. ZF_THAP | Immunoglobulin-like |
| 102. | KJV53819 | No hit | No hit | 1.ASN_GLYCOSYLATION<br>2.CK2_PHOSPHO_SITE<br>3.MYRISTYL<br>4.PKC_PHOSPHO_SITE<br>5.BIG1<br>6.PXA | 4-helical cytokines |
| 103. | KJV53820 | No hit | No hit | 1.ASN_GLYCOSYLATION<br>2.CK2_PHOSPHO_SITE | 4-helical cytokines |

| | | | | | |
|---|---|---|---|---|---|
| | | | | 3.MYRISTYL<br>4.PKC_PHOSPHO_SITE<br>5.BIG1<br>6.PXA | |
| 104. | KJV53914 | No hit | Magnesium Transporter | 1.ASN_GLYCOSYLATION<br>2.CAMP_PHOSPHO_SITE<br>3.CK2_PHOSPHO_SITE<br>4.MYRISTYL<br>5.PKC_PHOSPHO_SITE<br>6.Pumilio RNA-binding repeat profile<br>7.Domain of unknown function | Immunoglobulin-like |
| 105. | KJV53916 | No hit | Transmembrane region | 1.ASN_GLYCOSYLATION<br>2.CAMP_PHOSPHO_SITE<br>3.CK2_PHOSPHO_SITE<br>4.PKC_PHOSPHO_SITE<br>5.TYR_PHOSPHO_SITE<br>6. BRCA2 repeat | 4-helical up-and-down bundle |
| 106. | KJV53919 | No hit | PA14 domain-containing protein | 1.ASN_GLYCOSYLATION<br>2.CAMP_PHOSPHO_SITE<br>3.CK2_PHOSPHO_SITE<br>4.MYRISTYL<br>5.PKC_PHOSPHO_SITE<br>6.Serine-rich region<br>7.Threonine-rich region<br>8.Fibritin_C<br>Fibritin C-terminal region<br>9.H2-forming N5,N10-methylenetetrahydromethanopterin dehydrogenase | Immunoglobulin-like |
| 107. | KJV53935 | No hit | **No hit** | 1.ASN_GLYCOSYLATION<br>2.MYRISTYL<br>3.PKC_PHOSPHO_SITE<br>4. bacterial Ig-like domain 1 | DNA-binding 3-helical bundle |
| 108. | KJV53937 | No hit | **No hit** | 1.AMIDATION ASN_GLYCOSYLATION<br>2.CK2_PHOSPHO_SITE<br>3.PKC_PHOSPHO_SITE<br>4.TYR_PHOSPHO_SITE | Immunoglobulin-like |
| 109. | KJV53939 | No hit | **toprim domain protein** | 1.ASN_GLYCOSYLATION<br>2.CAMP_PHOSPHO_SITE<br>3.CK2_PHOSPHO_SITE<br>4.MYRISTYL | ferredoxin-like |
| 110. | KJV54139 | Serpin signature type protein | | 1.CAMP_PHOSPHO_SITE<br>2.CK2_PHOSPHO_SITE<br>3.MYRISTYL | 4-helical cytokines |
| 111. | KJV54140 | No hit | **lccl domain containing protein** | 1.CAMP_PHOSPHO_SITE<br>2.CK2_PHOSPHO_SITE<br>3.MYRISTYL | OB-fold |
| 112. | KJV54141 | No hit | **No hit** | 1.MYRISTYL<br>2.PKC_PHOSPHO_SITE | DNA-binding 3-helical bundle |
| 113. | KJV54143 | No hit | **No hit** | 1.PKC_PHOSPHO_SITE<br>2.CRYSTALLIN_BETA_GAMMA | OB-fold |
| 114. | KJV54146 | No hit | **PREDICTED: probable L-type lectin-domain containing receptor kinase II.1** | 1.CAMP_PHOSPHO_SITE<br>2.PKC_PHOSPHO_SITE<br>3.BIG1 | OB-fold |
| 115. | KJV54147 | No hit | **PREDICTED: ankyrin repeat and BTB/POZ domain-containing protein 2-like** | 1.CK2_PHOSPHO_SITE<br>2.MYRISTYL<br>3.PKC_PHOSPHO_SITE<br>4.FAST_1 | immunoglobulin-like |
| 116. | KJV54167 | No hit | **No hit** | 1.ASN_GLYCOSYLATION<br>2.CAMP_PHOSPHO_SITE<br>3.CK2_PHOSPHO_SITE<br>4.MYRISTYL<br>5.PKC_PHOSPHO_SITE<br>6.NLS_BP<br>7.BRCA2<br>8.VAR1 | OB-fold |
| 117. | KJV54168 | No hit | **No hit** | 1.CK2_PHOSPHO_SITE<br>2.MYRISTYL<br>**3.PKC_PHOSPHO_SITE**<br>**4.TYR_PHOSPHO_SITE** | 4-helical cytokines |

| | | | | | |
|---|---|---|---|---|---|
| | | | | **5.RAP** | |
| 118. | KJV54170 | No hit | **No hit** | 1.CK2_PHOSPHO_SITE<br>2.MYRISTYL<br>3.TYR_PHOSPHO_SITE<br>4.RAP | DNA-binding 3-helical bundle |
| 119. | KJV54341 | No hit | **Exonuclease domain protein** | 1.MYRISTYL<br>2.PKC_PHOSPHO_SITE | 4-helical cytokines |
| 120. | KJV54343 | No hit | **GHKL domain-containing protein** | 1.ASN_GLYCOSYLATION<br>2.RVT_1 | 4-helical cytokines |
| 121. | KJV54362 | No hit | No hit | 1.ASN_GLYCOSYLATION<br>2.CK2_PHOSPHO_SITE<br>3.MYRISTYL<br>4.BIG1<br>5.BRCA2 | DNA-binding 3-helical bundle |
| 122. | KJV54368 | No hit | **DUF490 domain-containing protein** | 1.CK2_PHOSPHO_SITE<br>2.MYRISTYL<br>3.PKC_PHOSPHO_SITE<br>4.FAST_1 | 4-helical cytokines |
| 123. | KJV54370 | No hit | **No hit** | 1.ASN_GLYCOSYLATION<br>2.CAMP_PHOSPHO_SITE<br>3.CK2_PHOSPHO_SITE<br>4.MYRISTYL<br>5.PKC_PHOSPHO_SITE<br>6.TYR_PHOSPHO_SITE<br>7.NLS_BP<br>8.BRCA2<br>9.VAR1 | DNA-binding 3-helical bundle |
| 124. | KJV54388 | No hit | **No hit** | 1.CK2_PHOSPHO_SITE<br>2.MYRISTYL<br>3.PKC_PHOSPHO_SITE<br>4.RAP | 4-helical cytokines |
| 125. | KJV54398 | No hit | **PREDICTED: BRCT domain-containing protein At4g02110-like** | CK2_PHOSPHO_SITE | OB-fold |
| 126. | KJV54413 | No hit | **No hit** | Empty | conA-like lectin/glucanases |
| 127. | KJV54438 | No hit | **MULTISPECIES: FimV domain-containing protein** | 1.AMIDATION<br>2.ASN_GLYCOSYLATION<br>3.MYRISTYL<br>4.PKC_PHOSPHO_SITE<br>5.05_PXD<br>6.NHL<br>7.PMBR | immunoglobulin-like |
| 128. | KJV54459 | No hit | No hit | 1.ASN_GLYCOSYLATION<br>2.CK2_PHOSPHO_SITE<br>3.PKC_PHOSPHO_SITE<br>4.TYR_PHOSPHO_SITE<br>5.IG_LIKE | DNA-binding 3-helical bundle |
| 129. | KJV54463 | No hit | No hit | 1.ASN_GLYCOSYLATION | DNA-binding 3-helical bundle |
| 130. | KJV54464 | No hit | None | 1. CK2_PHOSPHO_SITE<br>2. MYRISTYL<br>3. PKC_PHOSPHO_SITE | OB-fold |
| 131. | KJV54465 | No hit | No Hit | 1. AMIDATION<br>2. CK2_PHOSPHO_SITE<br>3. MYRISTYL | 4-helical cytokines |
| 132. | KJV54466 | No hit | Chromodomain-Helicase-DNA-binding protein 7 | 1.MYRISTYL<br>2.UPF0546 | 4-helical cytokines |
| 133. | KJV54488 | No hit | No hit | 1.ASN_GLYCOSYLATION<br>2.Bacterial Ig-like domain2 | OB-fold |
| 134. | KJV54489 | PTR2 family proton/oligo peptide symporters | No hit | 1.ASN_GLYCOSYLATION<br>2.CK2_PHOSPHO_SITE<br>3.PKC_PHOSPHO_SITE<br>4.PTR2_2 | OB-fold |
| 135. | KJV54492 | No hit | phage integrase family protein | 1.ASN_GLYCOSYLATION<br>2.CK2_PHOSPHO_SITE<br>3.MYRISTYL | Immunoglobulin-like |

| | | | | | |
|---|---|---|---|---|---|
| | | | | 3.PKC_PHOSPHO_SITE<br>4.NUMOD3 | |
| 136. | KJV54506 | No hit | EST/SMG like protein | 1. ASN_GLYCOSYLATION<br>2. CK2_PHOSPHO_SITE<br>3. MYRISTYL<br>4. PKC_PHOSPHO_SITE<br>5. Bacterial Ig-like domain2 (<br>6. ZF_THAP | Immunoglobulin-like |
| 137. | KJV54508 | No hit | DNA binding protein | 1.ASN_GLYCOSYLATION<br>2.CAMP_PHOSPHO_SITE<br>3.CK2_PHOSPHO_SITE<br>4.MYRISTYL<br>5.PKC_PHOSPHO_SITE<br>6.Pumilio RNA-binding repeat profile<br>7.Domain of unknown function | 4-helical cytokines |
| 138. | KJV54527 | No hit | No hit | 1.ASN_GLYCOSYLATION<br>2.CK2_PHOSPHO_SITE<br>3.MYRISTYL<br>4.PKC_PHOSPHO_SITE<br>5.BIG1<br>6.PXA | 4-helical cytokines |
| 139. | KJV54528 | No hit | Magnesium Transporter | 1.ASN_GLYCOSYLATION<br>2.CK2_PHOSPHO_SITE<br>3.MYRISTYL<br>4.PKC_PHOSPHO_SITE<br>5.BIG1<br>6.PXA | Immunoglobulin-like |
| 140. | KJV54529 | No hit | Transmembrane region | 1.ASN_GLYCOSYLATION<br>2.CAMP_PHOSPHO_SITE<br>3.CK2_PHOSPHO_SITE<br>4.PKC_PHOSPHO_SITE<br>5.TYR_PHOSPHO_SITE<br>6. BRCA2 repeat | 4-helical up-and-down bundle |
| 141. | KJV54530 | No hit | PA14 domain-containing protein | 1.ASN_GLYCOSYLATION<br>2.CAMP_PHOSPHO_SITE<br>3.CK2_PHOSPHO_SITE<br>4.MYRISTYL<br>5.PKC_PHOSPHO_SITE<br>6.Serine-rich region<br>7.Threonine-rich region<br>8.Fibritin_C region<br>9. H2-forming N5<br>10.methylenetetrahydromethanopterin dehydrogenase | Immunoglobulin-like |
| 142. | KJV54535 | No hit | No hit | 1.ASN_GLYCOSYLATION<br>2.MYRISTYL<br>3.PKC_PHOSPHO_SITE<br>4. bacterial Ig-like domain 1 | DNA-binding 3-helical bundle |
| 143. | KJV54539 | No hit | No hit | 1.AMIDATION ASN_GLYCOSYLATION<br>2.CK2_PHOSPHO_SITE<br>3.PKC_PHOSPHO_SITE<br>4.TYR_PHOSPHO_SITE | Immunoglobulin-like |
| 144. | KJV54540 | No hit | **Transmembrane** | Empty | DNA-binding 3-helical bundle |
| 145. | KJV54555 | No hit | **DUF2312 domain-containing protein** | 1.ASN_GLYCOSYLATION<br>2.PKC_PHOSPHO_SITE<br>3.YNNN_MF_00797<br>4.DUF2312 | 4-helical up-and-down bundle |
| 146. | KJV54556 | No hit | **phage integrase family protein** | 1.AMIDATION<br>2.PKC_PHOSPHO_SITE | DNA-binding 3-helical bundle |
| 147. | KJV54581 | No hit | No hit | 1.PKC_PHOSPHO_SITE | Belta-grasp |
| 148. | KJV54582 | No hit | No hit | 1.ASN_GLYCOSYLATION<br>2.CAMP_PHOSPHO_SITE<br>3.CK2_PHOSPHO_SITE<br>4.PKC_PHOSPHO_SITE<br>5.DUF461 | immunoglobulin-like |
| 149. | KJV54587 | No hit | **dnaA N-terminal domain protein** | 1.ASN_GLYCOSYLATION<br>2.CAMP_PHOSPHO_SIT<br>3.CK2_PHOSPHO_SITE<br>4.MYRISTYL<br>5.PKC_PHOSPHO_SITE<br>6.NUMOD3 | OB-fold |

| | | | | | |
|---|---|---|---|---|---|
| 150. | KJV54614 | No hit | No hit | 1.ASN_GLYCOSYLATION<br>2.CK2_PHOSPHO_SITE<br>3.PKC_PHOSPHO_SITE<br>4.MYRISTYL | 4-helical cytokines |
| 151. | KJV54616 | No hit | No hit | 1.ASN_GLYCOSYLATION<br>2.CK2_PHOSPHO_SITE<br>3.PKC_PHOSPHO_SITE<br>4.TYR_PHOSPHO_SITE | 4-helical cytokines |
| 152. | KJV54666 | No hit | No hit | 1.ASN_GLYCOSYLATION<br>2.CK2_PHOSPHO_SITE<br>3.MYRISTYL<br>4.PKC_PHOSPHO_SITE | OB-fold |
| 153. | KJV54670 | No hit | peptide ABC transporter substrate-binding protein | 1.AMIDATION<br>2.ASN_GLYCOSYLATION<br>3.CAMP_PHOSPHO_SITE<br>4.CK2_PHOSPHO_SITE<br>5.MYRISTYL<br>6.PKC_PHOSPHO_SITE | immunoglobulin-like |
| 154. | KJV54671 | No hit | No hit | 1.ASN_GLYCOSYLATION<br>2.MYRISTYL<br>3.PKC_PHOSPHO_SITE<br>4.PKC_PHOSPHO_SITE | OB-fold |
| 155. | KJV54701 | No hit | Transmembrane | 1.ASN_GLYCOSYLATION<br>2.CAMP_PHOSPHO_SITE<br>3.PKC_PHOSPHO_SITE<br>4.BIG1 | immunoglobulin-like |
| 156. | KJV54705 | No hit | magnesium/cobalt efflux protein | empty | 4-helical cytokines |
| 157. | KJV54707 | No hit | No hit | 1.ASN_GLYCOSYLATION<br>2.MYRISTYL | cytochrome c |
| 158. | KJV54735 | No hit | Transmembrane | 1.ASN_GLYCOSYLATION<br>2.PKC_PHOSPHO_SITE<br>3.BIG1<br>4.CW_binding_2 | DNA-binding 3-helical bundle |
| 159. | KJV54779 | No hit | No hit | 1.MYRISTYL | 4-helical cytokines |
| 160. | KJV54783 | No hit | No hit | 1.AMIDATION<br>2.ASN_GLYCOSYLATION<br>3.CAMP_PHOSPHO_SITE<br>4.CK2_PHOSPHO_SITE<br>5.MYRISTYL<br>6.PKC_PHOSPHO_SITE<br>7.TYR_PHOSPHO_SITE<br>8.Pepsin-I3 | 4-helical cytokines |
| 161. | KJV54785 | No hit | No hit | 1.ASN_GLYCOSYLATION<br>2.CK2_PHOSPHO_SITE<br>3.MYRISTYL<br>4.PKC_PHOSPHO_SITE<br>5.TYR_PHOSPHO_SITE | 4-helical cytokines |
| 162. | KJV54789 | No hit | dnaA N-terminal domain protein | 1.CK2_PHOSPHO_SITE<br>2.MYRISTYL<br>3.PKC_PHOSPHO_SITE | 4-helical cytokines |
| 163. | KJV54829 | No hit | | 1.ASN_GLYCOSYLATION<br>2.CAMP_PHOSPHO_SITE<br>3.CK2_PHOSPHO_SITE<br>4.MYRISTYL<br>5.PKC_PHOSPHO_SITE<br>6.PFTA<br>7.Hexapep | Belta-grasp |
| 164. | KJV54870 | No hit | Transmembrane | 1.ASN_GLYCOSYLATION<br>2.CK2_PHOSPHO_SITE<br>3.MYRISTYL<br>4.PKC_PHOSPHO_SITE<br>5.DUF308 | Cupredoxins |
| 165. | KJV54874 | No hit | Transposase, YhgA-like family protein | 1.CK2_PHOSPHO_SITE<br>2.MYRISTYL<br>3.PKC_PHOSPHO_SITE | OB-fold |
| 166. | KJV54877 | No hit | No hit | 1.ASN_GLYCOSYLATION<br>2.CAMP_PHOSPHO_SITE<br>3.CK2_PHOSPHO_SITE<br>4.MYRISTYL<br>5.PKC_PHOSPHO_SITE | OB-fold |
| 167. | KJV54878 | No hit | No hit | 1.PKC_PHOSPHO_SITE | DNA-binding 3-helical bundle |

| | | | | | |
|---|---|---|---|---|---|
| 168. | KJV54879 | No hit | No hit | 1.CK2_PHOSPHO_SITE<br>2. PKC_PHOSPHO_SITE | Belta-grasp |
| 169. | KJV54906 | No hit | secA DEAD-like domain protein | 1.PKC_PHOSPHO_SITE<br>2.TYR_PHOSPHO_SITE | EF-hand |
| 170. | KJV54907 | No hit | No hit | 1.ASN_GLYCOSYLATION<br>2.MYRISTYL | immunoglobulin-like |
| 171. | KJV54908 | No hit | No hit | 1.CK2_PHOSPHO_SITE<br>2.PKC_PHOSPHO_SITE<br>3.TYR_PHOSPHO_SITE | thioredoxin-like |
| 172. | KJV54909 | No hit | No hit | 1.ASN_GLYCOSYLATION<br>2.CAMP_PHOSPHO_SITE<br>3.CK2_PHOSPHO_SITE<br>4.MYRISTYL<br>5.PKC_PHOSPHO_SITE<br>6.TYR_PHOSPHO_SITE<br>7.DNA_pol3_theta | immunoglobulin-like |
| 173. | KJV54913 | No hit | No hit | 1.AMIDATION<br>2.CAMP_PHOSPHO_SITE<br>3.CK2_PHOSPHO_SITE<br>4.MYRISTYL<br>5.SRR<br>6.UPF0546 | 4-helical cytokines |
| 174. | KJV54946 | No hit | No hit | 1.AMIDATION<br>2.CAMP_PHOSPHO_SITE<br>3.CK2_PHOSPHO_SITE<br>4.MYRISTYL<br>5.SRR<br>6.UPF0546 | immunoglobulin-like |
| 175. | KJV54971 | No hit | dnaA N-terminal domain protein | 1.ASN_GLYCOSYLATION<br>2.PKC_PHOSPHO_SITE<br>3.ASN_RICH<br>4.DUF270<br>5.SKN1<br>6.NUMOD3 | 4-helical cytokines |
| 176. | KJV54975 | No hit | peptide ABC transporter substrate-binding protein | 1.AMIDATION<br>2.ASN_GLYCOSYLATION<br>3.CAMP_PHOSPHO_SITE<br>4.CK2_PHOSPHO_SITE<br>5.MYRISTYL<br>6.PKC_PHOSPHO_SITE | immunoglobulin-like |
| 177. | KJV54977 | No hit | No hit | 1.ASN_GLYCOSYLATION<br>2.MYRISTYL<br>3.PKC_PHOSPHO_SITE<br>4.PKC_PHOSPHO_SITE | OB-fold |
| 178. | KJV54978 | No hit | Transmembrane | 1.ASN_GLYCOSYLATION<br>2.CAMP_PHOSPHO_SITE<br>3.PKC_PHOSPHO_SITE<br>4.BIG1 | immunoglobulin-like |
| 179. | KJV55027 | No hit | magnesium/cobalt efflux protein | empty | 4-helical cytokines |
| 180. | KJV55028 | No hit | No hit | 1.ASN_GLYCOSYLATION<br>2.MYRISTYL | cytochrome c |
| 181. | KJV55033 | No hit | Transmembrane | 1.ASN_GLYCOSYLATION<br>2.PKC_PHOSPHO_SITE<br>3.BIG1<br>4.CW_binding_2 | DNA-binding 3-helical bundle |
| 182. | KJV55034 | No hit | No hit | 1.MYRISTYL | 4-helical cytokines |
| 183. | KJV55035 | No hit | No hit | 1.AMIDATION<br>2.ASN_GLYCOSYLATION<br>3.CAMP_PHOSPHO_SITE<br>4.CK2_PHOSPHO_SITE<br>5.MYRISTYL<br>6.PKC_PHOSPHO_SITE<br>7.TYR_PHOSPHO_SITE<br>8.Pepsin-I3 | 4-helical cytokines |
| 184. | KJV55037 | No hit | **MULTISPECIES: FimV domain-containing protein** | 1.AMIDATION<br>2.ASN_GLYCOSYLATION<br>3.MYRISTYL<br>4.PKC_PHOSPHO_SITE<br>5.05_PXD<br>6.NHL<br>7.PMBR | immunoglobulin-like |

| | | | | | |
|---|---|---|---|---|---|
| 185. | KJV55079 | No hit | No hit | 1.ASN_GLYCOSYLATION<br>2.CK2_PHOSPHO_SITE<br>3.PKC_PHOSPHO_SITE<br>4.TYR_PHOSPHO_SITE<br>5.IG_LIKE | DNA-binding 3-helical bundle |
| 186. | KJV55080 | No hit | No hit | 1.ASN_GLYCOSYLATION | DNA-binding 3-helical bundle |
| 187. | KJV55165 | No hit | None | 1. CK2_PHOSPHO_SITE<br>2. MYRISTYL<br>3. PKC_PHOSPHO_SITE | OB-fold |
| 188. | KJV55217 | No hit | No Hit | 1. AMIDATION<br>2. CK2_PHOSPHO_SITE<br>3. MYRISTYL | 4-helical cytokines |
| 189. | KJV55220 | No hit | Chromodomain-Helicase-DNA-binding protein 7 | 1.MYRISTYL<br>2.UPF0546 | 4-helical cytokines |
| 190. | KJV55222 | No hit | No hit | 1.ASN_GLYCOSYLATION<br>2.Bacterial Ig-like domain2 | OB-fold |
| 191. | KJV55225 | PTR2 family proton/oligo peptide supporters | No hit | 1.ASN_GLYCOSYLATION<br>2.CK2_PHOSPHO_SITE<br>3.PKC_PHOSPHO_SITE<br>4.PTR2_2 | OB-fold |
| 192. | KJV55228 | No hit | Transmembrane | 1.PKC_PHOSPHO_SITE | immunoglobulin-like |
| 193. | KJV55230 | No hit | DNA repair protein RecN | 1.ASN_GLYCOSYLATION<br>2.CK2_PHOSPHO_SITE<br>3.MYRISTYL<br>4.PKC_PHOSPHO_SITE<br>5.BIG1<br>6.DUF618 | 4-helical cytokines |
| 194. | KJV55231 | No hit | **No hit** | 1.ASN_GLYCOSYLATION<br>2.PKC_PHOSPHO_SITE<br>3.BIG1 | Immunoglobulin-like |
| 195. | KJV55284 | Not hit | **Transmembrane** | 1.ASN_GLYCOSYLATION<br>2.CK2_PHOSPHO_SITE<br>3.MYRISTYL<br>4.PKC_PHOSPHO_SITE | Immunoglobulin-like |
| 196. | KJV55290 | No hit | **No hit** | 1.ASN_GLYCOSYLATION<br>2.MYRISTYL<br>3.PKC_PHOSPHO_SITE<br>4. bacterial Ig-like domain 1 | DNA-binding 3-helical bundle |
| 197. | KJV55293 | No hit | **No hit** | 1.AMIDATION ASN_GLYCOSYLATION<br>2.CK2_PHOSPHO_SITE<br>3.PKC_PHOSPHO_SITE<br>4.TYR_PHOSPHO_SITE | Immunoglobulin-like |
| 198. | KJV55294 | No hit | **toprim domain protein** | 1.CK2_PHOSPHO_SITE<br>2.MYRISTYL | ferredoxin-like |
| 199. | KJV55300 | No hit | **No hit** | 1.ASN_GLYCOSYLATION<br>2.CAMP_PHOSPHO_SITE<br>3.CK2_PHOSPHO_SITE<br>4.PKC_PHOSPHO_SITE<br>5.SERPIN | 4-helical cytokines |
| 200. | KJV55334 | No hit | **No hit** | 1.ASN_GLYCOSYLATION<br>2.CAMP_PHOSPHO_SITE<br>3.CK2_PHOSPHO_SITE<br>4.PKC_PHOSPHO_SITE<br>5.TYR_PHOSPHO_SITE<br>6. BRCA2 repeat | 4-helical up-and-down bundle |
| 201. | KJV55336 | No hit | **No hit** | 1.ASN_GLYCOSYLATION<br>2.CAMP_PHOSPHO_SITE<br>3.CK2_PHOSPHO_SITE<br>4.MYRISTYL<br>5.PKC_PHOSPHO_SITE | Immunoglobulin-like |
| 202. | KJV55342 | No hit | None | 1. CK2_PHOSPHO_SITE<br>2. MYRISTYL<br>3. PKC_PHOSPHO_SITE | OB-fold |
| 203. | KJV55344 | No hit | DNA binding protein, | 1. AMIDATION<br>2. CK2_PHOSPHO_SITE<br>3. MYRISTYL | 4-helical cytokines |
| 204. | KJV55346 | No hit | Chromodomain-Helicase-DNA-binding protein 7 | 1.MYRISTYL<br>2. UPF0546 | 4-helical cytokines |

| | | | | | |
|---|---|---|---|---|---|
| 205. | KJV55409 | No hit | No hit | 1.ASN_GLYCOSYLATION<br>2.Bacterial Ig-like domain2 | OB-fold |
| 206. | KJV55456 | PTR2 family proton/oligo peptide symporters | No hit | 1.ASN_GLYCOSYLATION<br>2.CK2_PHOSPHO_SITE<br>3.MYRISTYL<br>3.PKC_PHOSPHO_SITE<br>4.NUMOD3 | OB-fold |
| 207. | KJV55458 | No hit | phage integrase family protein | 1.ASN_GLYCOSYLATION<br>2.CK2_PHOSPHO_SITE<br>3.PKC_PHOSPHO_SITE<br>4.PTR2_2 | Immunoglobulin-like |
| 208. | KJV55462 | No hit | EST/SMG like protein | 1. ASN_GLYCOSYLATION<br>2. CK2_PHOSPHO_SITE<br>3. MYRISTYL<br>4. PKC_PHOSPHO_SITE<br>5. Bacterial Ig-like domain2 (<br>6. ZF_THAP | Immunoglobulin-like |
| 209. | KJV55465 | No hit | No hit | 1.ASN_GLYCOSYLATION<br>2.CK2_PHOSPHO_SITE<br>3.MYRISTYL<br>4.PKC_PHOSPHO_SITE<br>5.BIG1<br>6.PXA | 4-helical cytokines |
| 210. | KJV55474 | No hit | No hit | 1.ASN_GLYCOSYLATION<br>2.CK2_PHOSPHO_SITE<br>3.MYRISTYL<br>4.PKC_PHOSPHO_SITE<br>5.BIG1<br>6.PXA | 4-helical cytokines |
| 211. | KJV55527 | No hit | Magnesium Transporter | 1.ASN_GLYCOSYLATION<br>2.CAMP_PHOSPHO_SITE<br>3.CK2_PHOSPHO_SITE<br>4.MYRISTYL<br>5.PKC_PHOSPHO_SITE<br>6.Pumilio RNA-binding repeat profile<br>7.Domain of unknown function | Immunoglobulin-like |
| 212. | KJV55533 | No hit | Transmembrane region | 1.ASN_GLYCOSYLATION<br>2.CAMP_PHOSPHO_SITE<br>3.CK2_PHOSPHO_SITE<br>4.PKC_PHOSPHO_SITE<br>5.TYR_PHOSPHO_SITE<br>6. BRCA2 repeat | 4-helical up-and-down bundle |
| 213. | KJV55535 | No hit | PA14 domain-containing protein | 1.ASN_GLYCOSYLATION<br>2.CAMP_PHOSPHO_SITE<br>3.CK2_PHOSPHO_SITE<br>4.MYRISTYL<br>5.PKC_PHOSPHO_SITE<br>6.Serine-rich region<br>7.Threonine-rich region<br>8.Fibritin_C<br>Fibritin C-terminal region<br>9.H2-forming N5,N10-methylenetetrahydromethanopterin dehydrogenase | Immunoglobulin-like |
| 214. | KJV55597 | No hit | No hit | 1.ASN_GLYCOSYLATION<br>2.MYRISTYL<br>3.PKC_PHOSPHO_SITE<br>4. bacterial Ig-like domain 1 | DNA-binding 3-helical bundle |
| 215. | KJV55599 | No hit | No hit | 1.AMIDATION ASN_GLYCOSYLATION<br>2.CK2_PHOSPHO_SITE<br>3.PKC_PHOSPHO_SITE<br>4.TYR_PHOSPHO_SITE | Immunoglobulin-like |
| 216. | KJV55659 | No hit | Transmembrane region | 1.ASN_GLYCOSYLATION<br>2.CK2_PHOSPHO_SITE<br>3.MYRISTYL<br>4.PKC_PHOSPHO_SIT<br>5.TYR_PHOSPHO_SITE<br>6.ABL | 4-helical cytokines |
| 217. | KJV55666 | No hit | **No hit** | 1.CAMP_PHOSPHO_SITE<br>2.NLS_BP<br>3.14_MPO<br>4.R3H<br>5.NLS_BP | DNA-binding 3-helical bundle |

| | | | | | |
|---|---|---|---|---|---|
| 218. | KJV55667 | No hit | **No hit** | 1.CAMP_PHOSPHO_SITE<br>2.14_MPO<br>3.NLS_BP<br>4.R3H<br>5.NLS_BP | DNA-binding 3-helical bundle |
| 219. | KJV55680 | No hit | **No hit** | 1.ASN_GLYCOSYLATION<br>2.CK2_PHOSPHO_SITE<br>3.MYRISTYL<br>4.TYR_PHOSPHO_SITE<br>5.HAND_2 | OB-fold |
| 220. | KJV55734 | No hit | Transmembrane protein | 1.ASN_GLYCOSYLATION<br>2.CK2_PHOSPHO_SITE<br>3.MYRISTYL<br>4.PKC_PHOSPHO_SITE<br>5.ASN_RICH<br>6.BIG1<br>7.PROKAR_LIPOPROTEIN<br>8.OGFr_III | Immunoglobulin-like |
| 221. | KJV55744 | No hit | polyribonucleotide nucleotidyltransfe rase | 1.PKC_PHOSPHO_SITE | DNA-binding 3-helical bundle |
| 222. | KJV55746 | No hit | **No hit** | 1.ASN_GLYCOSYLATION<br>2.CAMP_PHOSPHO_SITE<br>3.CK2_PHOSPHO_SITE<br>4.MYRISTYL<br>5.TYR_PHOSPHO_SITE<br>6.HIS_RICH<br>7.Annexin | 4-helical cytokines |
| 223. | KJV55751 | No hit | **DUF167 domain** | 1.ASN_GLYCOSYLATION<br>2.CK2_PHOSPHO_SITE | Cupredoxins |
| 224. | KJV55806 | No Hit | **DUF167 domain** | 1.ASN_GLYCOSYLATION<br>2.CK2_PHOSPHO_SITE<br>3.MYRISTYL<br>4.YGGU_MF_00634<br>5.THIOREDOXIN_2<br>6.DUF167 | DNA-binding 3-helical bundle |
| 225. | KJV55810 | No hit | Transmembrane | 1.PKC_PHOSPHO_SITE | immunoglobulin-like |
| 226. | KJV55821 | No hit | DNA repair protein RecN | 1.ASN_GLYCOSYLATION<br>2.CK2_PHOSPHO_SITE<br>3.MYRISTYL<br>4.PKC_PHOSPHO_SITE<br>5.BIG1<br>6.DUF618 | 4-helical cytokines |
| 227. | KJV55871 | No hit | **No hit** | 1.ASN_GLYCOSYLATION<br>2.PKC_PHOSPHO_SITE<br>3.BIG1 | Immunoglobulin-like |
| 228. | KJV55874 | Not hit | **Transmembrane** | 1.ASN_GLYCOSYLATION<br>2.CK2_PHOSPHO_SITE<br>3.MYRISTYL<br>4.PKC_PHOSPHO_SITE<br>5.AMA-1<br>6.PAAR_motif<br>7.SKG6 | Immunoglobulin-like |
| 229. | KJV55882 | No hit | **No hit** | 1.ASN_GLYCOSYLATION<br>2.CK2_PHOSPHO_SITE<br>3.MYRISTYL<br>4.PKC_PHOSPHO_SITE<br>5.TYR_PHOSPHO_SITE<br>6.ESTERASE<br>7.Phage_fiber | Immunoglobulin-like |
| 230. | KJV55884 | No hit | **No hit** | Empty | DNA-binding 3-helical bundle |
| 231. | KJV55885 | | **d1l7va_ domain & d1hw1a1 domain** | 1.ASN_GLYCOSYLATION<br>2.CAMP_PHOSPHO_SITE<br>3.MYRISTYL<br>4.PKC_PHOSPHO_SITE<br>5.TAFH<br>6.Hexapep<br>7.NUMOD3 | Belta-grasp |
| 232. | KJV55951 | No hit | **d1l9ha_ domain** | 1. PKC_PHOSPHO_SITE | 4-helical cytokines |
| 233. | KJV55957 | No hit | **dnaA N-terminal domain protein** | 1.ASN_GLYCOSYLATION<br>2.CK2_PHOSPHO_SITE | DNA-binding 3-helical bundle |

| | | | | | |
|---|---|---|---|---|---|
| | | | | 3.ASN_RICH<br>4.DUF270<br>5.SKN1<br>6.NUMOD3 | |
| 234. | KJV55958 | No hit | **Transmembrane** | 1.ASN_GLYCOSYLATION<br>2.CK2_PHOSPHO_SITE<br>3.PKC_PHOSPHO_SITE | dnaA N-terminal domain protein |
| 235. | KJV55962 | No hit | No hit | CK2_PHOSPHO_SITE | OB-fold |
| 236. | KJV56036 | ApaG domain profile | **Co2+/Mg2+ efflux protein ApaG** | 1.CK2_PHOSPHO_SITE<br>2. MYRISTYL<br>3.PKC_PHOSPHO_SITE<br>4.APAG_MF_00791<br>5.APAG<br>6.DUF525 | immunoglobulin-like |
| 237. | KJV56037 | No hit | No hit | empty | 4-helical cytokines |
| 238. | KJV56040 | No hit | No hit | 1.ASN_GLYCOSYLATION<br>2.PKC_PHOSPHO_SITE<br>3.PFTA | 4-helical cytokines |
| 239. | KJV56045 | No hit | **phage integrase family protein** | 1.AMIDATION<br>2.ASN_GLYCOSYLATION<br>3.CK2_PHOSPHO_SITE<br>4.MYRISTYL<br>5.PKC_PHOSPHO_SITE<br>6.C_CAP_COFACTOR_C<br>7.PEPCK | DNA-binding 3-helical bundle |
| 240. | KJV56047 | No hit | putative transposase like protein | 1.CK2_PHOSPHO_SITE<br>2.MYRISTYL<br>3.Transposase_31 | cytochrome c |
| 241. | KJV56053 | No hit | No hit | empty | EF-hand |
| 242. | KJV56131 | No hit | d1fjja_ domain | 1.ASN_GLYCOSYLATION<br>2.MYRISTYL<br>3.PKC_PHOSPHO_SITE<br>4.PKC_PHOSPHO_SITE | mall inhibitors, toxins, lectins |
| 243. | KJV56132 | No hit | IGR00701 family protein | 1.ASN_GLYCOSYLATION<br>2.CAMP_PHOSPHO_SITE<br>3.CK2_PHOSPHO_SITE<br>4.PKC_PHOSPHO_SITE<br>5.G_PROTEIN_RECEP_F1_2<br>6.LDLRB<br>7.UPF0093 | Cupredoxins |
| 244. | KJV56137 | No hit | **Transmembrane** | 1.CK2_PHOSPHO_SITE<br>2.PKC_PHOSPHO_SITE<br>3.TYR_PHOSPHO_SITE | 4-helical cytokines |
| 245. | KJV56142 | No hit | **Transmembrane** | 1.AMIDATION<br>2.ASN_GLYCOSYLATION<br>3.CAMP_PHOSPHO_SITE<br>4.CK2_PHOSPHO_SITE<br>5.MYRISTYL<br>6.PKC_PHOSPHO_SITE<br>7.TYR_PHOSPHO_SITE<br>8.BRCA2 | viral coat and capsid proteins |
| 246. | KJV56143 | No hit | | 1.ASN_GLYCOSYLATION<br>2.CAMP_PHOSPHO_SITE<br>3.CK2_PHOSPHO_SITE<br>4.MYRISTYL<br>5.PKC_PHOSPHO_SITE | 4-helical cytokines |
| 247. | KJV56203 | No hit | **B3/B4 domain-containing protein** | 1.ASN_GLYCOSYLATION<br>2.MYRISTYL<br>3.PKC_PHOSPHO_SITE | Cytochrome c |
| 248. | KJV56204 | No hit | **Transmembrane** | 1.PKC_PHOSPHO_SITE | OB-fold |
| 249. | KJV56205 | No hit | No hit | 1.ASN_GLYCOSYLATION<br>2.CK2_PHOSPHO_SITE<br>3.PKC_PHOSPHO_SITE<br>4.ILE_RICH | immunoglobulin-like |
| 250. | KJV56209 | No hit | **Transmembrane** | Empty | DNA-binding 3-helical bundle |

| | | | | | |
|---|---|---|---|---|---|
| 251. | KJV56211 | No hit | No hit | 1.ASN_GLYCOSYLATION<br>2.CK2_PHOSPHO_SITE<br>3.MYRISTYL<br>4.PKC_PHOSPHO_SITE<br>5.BIG1<br>6.PXA | 4-helical cytokines |
| 252. | KJV56214 | No hit | No hit | 1.ASN_GLYCOSYLATION<br>2.CK2_PHOSPHO_SITE<br>3.MYRISTYL<br>4.PKC_PHOSPHO_SITE<br>5.BIG1<br>6.PXA | 4-helical cytokines |
| 253. | KJV56219 | No hit | Magnesium Transporter | 1.ASN_GLYCOSYLATION<br>2.CAMP_PHOSPHO_SITE<br>3.CK2_PHOSPHO_SITE<br>4.MYRISTYL<br>5.PKC_PHOSPHO_SITE<br>6.Pumilio RNA-binding repeat profile<br>7.Domain of unknown function | Immunoglobulin-like |
| 254. | KJV56296 | No hit | Transmembrane region | 1.ASN_GLYCOSYLATION<br>2.CAMP_PHOSPHO_SITE<br>3.CK2_PHOSPHO_SITE<br>4.PKC_PHOSPHO_SITE<br>5.TYR_PHOSPHO_SITE<br>6. BRCA2 repeat | 4-helical up-and-down bundle |
| 255. | KJV56300 | No hit | PA14 domain-containing protein | 1.ASN_GLYCOSYLATION<br>2.CAMP_PHOSPHO_SITE<br>3.CK2_PHOSPHO_SITE<br>4.MYRISTYL<br>5.PKC_PHOSPHO_SITE | Immunoglobulin-like |
| 256. | KJV56304 | No hit | No hit | 1.ASN_GLYCOSYLATION<br>2.MYRISTYL<br>3.PKC_PHOSPHO_SITE<br>4. bacterial Ig-like domain 1 | DNA-binding 3-helical bundle |
| 257. | KJV56305 | No hit | No hit | 1.AMIDATION ASN_GLYCOSYLATION<br>2.CK2_PHOSPHO_SITE<br>3.PKC_PHOSPHO_SITE<br>4.TYR_PHOSPHO_SITE | Immunoglobulin-like |
| 258. | KJV56385 | No hit | Transmembrane region | 1.ASN_GLYCOSYLATION<br>2.CK2_PHOSPHO_SITE<br>3.MYRISTYL<br>4.PKC_PHOSPHO_SIT<br>5.TYR_PHOSPHO_SITE<br>6.ABL | 4-helical cytokines |
| 259. | KJV56401 | No hit | **No hit** | 1.CAMP_PHOSPHO_SITE<br>2.NLS_BP<br>3.14_MPO<br>4.R3H<br>5.NLS_BP | DNA-binding 3-helical bundle |
| 260. | KJV56402 | No hit | **No hit** | 1.CAMP_PHOSPHO_SITE<br>2.14_MPO<br>3.NLS_BP<br>4.R3H<br>5.NLS_BP | DNA-binding 3-helical bundle |
| 261. | KJV56404 | No hit | **No hit** | 1.ASN_GLYCOSYLATION<br>2.CK2_PHOSPHO_SITE<br>3.MYRISTYL<br>4.TYR_PHOSPHO_SITE<br>5.HAND_2 | OB-fold |
| 262. | KJV56408 | No hit | Transmembrane protein | 1.ASN_GLYCOSYLATION<br>2.CK2_PHOSPHO_SITE<br>3.MYRISTYL<br>4.PKC_PHOSPHO_SITE<br>5.ASN_RICH<br>6.BIG1<br>7.PROKAR_LIPOPROTEIN<br>8.OGFr_III | Immunoglobulin-like |
| 263. | KJV56474 | No hit | polyribonucleotide nucleotidyltransferase | 1.PKC_PHOSPHO_SITE | DNA-binding 3-helical bundle |
| 264. | KJV56480 | No hit | **No hit** | 1.ASN_GLYCOSYLATION<br>2.CAMP_PHOSPHO_SITE<br>3.CK2_PHOSPHO_SITE<br>4.MYRISTYL<br>5.TYR_PHOSPHO_SITE | 4-helical cytokines |

| | | | | | |
|---|---|---|---|---|---|
| | | | | 6.HIS_RICH<br>7.Annexin | |
| 265. | KJV56570 | No hit | **DUF167 domain** | 1.ASN_GLYCOSYLATION<br>2.CK2_PHOSPHO_SITE | Cupredoxins |
| 266. | KJV56573 | No Hit | **DUF167 domain** | 1.ASN_GLYCOSYLATION<br>2.CK2_PHOSPHO_SITE<br>3.MYRISTYL<br>4.YGGU_MF_00634<br>5.THIOREDOXIN_2<br>6.DUF167 | DNA-binding 3-helical bundle |
| 267. | KJV56574 | No hit | Transmembrane | 1.PKC_PHOSPHO_SITE | Immunoglobulin-like |
| 268. | KJV56575 | No hit | DNA repair protein RecN | 1.ASN_GLYCOSYLATION<br>2.CK2_PHOSPHO_SITE<br>3.MYRISTYL<br>4.PKC_PHOSPHO_SITE<br>5.BIG1<br>6.DUF618 | 4-helical cytokines |
| 269. | KJV56581 | No hit | **No hit** | 1.ASN_GLYCOSYLATION<br>2.PKC_PHOSPHO_SITE<br>3.BIG1 | Immunoglobulin-like |
| 270. | KJV56583 | Not hit | **Transmembrane** | 1.ASN_GLYCOSYLATION<br>2.CK2_PHOSPHO_SITE<br>3.MYRISTYL<br>4.PKC_PHOSPHO_SITE<br>5.AMA-1<br>6.PAAR_motif<br>7.SKG6 | Immunoglobulin-like |
| 271. | KJV56669 | No hit | **No hit** | 1.ASN_GLYCOSYLATION<br>2.CK2_PHOSPHO_SITE<br>3.MYRISTYL<br>4.PKC_PHOSPHO_SITE<br>5.TYR_PHOSPHO_SITE<br>6.ESTERASE<br>7.Phage_fiber | Immunoglobulin-like |
| 272. | KJV56673 | No hit | peptide ABC transporter substrate-binding protein | 1.AMIDATION<br>2.ASN_GLYCOSYLATION<br>3.CAMP_PHOSPHO_SITE<br>4.CK2_PHOSPHO_SITE<br>5.MYRISTYL<br>6.PKC_PHOSPHO_SITE | immunoglobulin-like |
| 273. | KJV56675 | No hit | No hit | 1.ASN_GLYCOSYLATION<br>2.MYRISTYL<br>3.PKC_PHOSPHO_SITE<br>4.PKC_PHOSPHO_SITE | OB-fold |
| 274. | KJV56683 | No hit | Transmembrane | 1.ASN_GLYCOSYLATION<br>2.CAMP_PHOSPHO_SITE<br>3.PKC_PHOSPHO_SITE<br>4.BIG1 | immunoglobulin-like |
| 275. | KJV56684 | No hit | magnesium/cobalt efflux protein | empty | 4-helical cytokines |
| 276. | KJV56688 | No hit | No hit | 1.ASN_GLYCOSYLATION<br>2.MYRISTYL | cytochrome c |
| 277. | KJV56690 | No hit | Transmembrane | 1.ASN_GLYCOSYLATION<br>2.PKC_PHOSPHO_SITE<br>3.BIG1<br>4.CW_binding_2 | DNA-binding 3-helical bundle |
| 278. | KJV56780 | No hit | No hit | 1.MYRISTYL | 4-helical cytokines |
| 279. | KJV56783 | No hit | No hit | 1.AMIDATION<br>2.ASN_GLYCOSYLATION<br>3.CAMP_PHOSPHO_SITE<br>4.CK2_PHOSPHO_SITE<br>5.MYRISTYL<br>6.PKC_PHOSPHO_SITE<br>7.TYR_PHOSPHO_SITE | 4-helical cytokines |
| 280. | KJV56789 | No hit | No hit | 1.CK2_PHOSPHO_SITE<br>2.MYRISTYL | OB-fold |
| 281. | KJV56930 | No hit | Transmembrane | 1.ASN_GLYCOSYLATION<br>2.CAMP_PHOSPHO_SITE<br>3.CK2_PHOSPHO_SITE<br>4.PKC_PHOSPHO_SITE | 4-helical cytokines |

| | | | | | |
|---|---|---|---|---|---|
| 282. | KJV56935 | No hit | No hit | 1.ASN_GLYCOSYLATION<br>2.CK2_PHOSPHO_SITE<br>3.PKC_PHOSPHO_SITE<br>4.TYR_PHOSPHO_SITE | 4-helical cytokines |
| 283. | KJV57117 | No hit | No hit | 1.ASN_GLYCOSYLATION<br>2.CK2_PHOSPHO_SITE<br>3.MYRISTYL | OB-fold |
| 284. | KJV57120 | No hit | Peptide ABC transporter substrate-binding protein | 1.AMIDATION<br>2.ASN_GLYCOSYLATION<br>3.CAMP_PHOSPHO_SITE<br>4.CK2_PHOSPHO_SITE<br>5.MYRISTYL | Immunoglobulin-like |
| 285. | KJV57129 | No hit | No hit | 1.ASN_GLYCOSYLATION<br>2.MYRISTYL<br>3.PKC_PHOSPHO_SITE<br>4.PKC_PHOSPHO_SITE | OB-fold |
| 286. | KJV57131 | No hit | Transmembrane | 1.ASN_GLYCOSYLATION<br>2.CAMP_PHOSPHO_SITE<br>3.PKC_PHOSPHO_SITE<br>4.BIG1 | Immunoglobulin-like |
| 287. | KJV57139 | No hit | Magnesium/cobalt efflux protein | empty | 4-helical cytokines |
| 288. | KJV57144 | No hit | No hit | 1.ASN_GLYCOSYLATION<br>2.MYRISTYL | Cytochrome c |
| 289. | KJV57200 | No hit | Transmembrane | 1.ASN_GLYCOSYLATION<br>2.PKC_PHOSPHO_SITE<br>3.BIG1<br>4.CW_binding_2 | DNA-binding 3-helical bundle |
| 290. | KJV57203 | No hit | No hit | 1.MYRISTYL | 4-helical cytokines |
| 291. | KJV57204 | No hit | No hit | 1.AMIDATION<br>2.ASN_GLYCOSYLATION<br>3.CAMP_PHOSPHO_SITE<br>4.CK2_PHOSPHO_SITE<br>5.MYRISTYL<br>6.PKC_PHOSPHO_SITE<br>7.TYR_PHOSPHO_SITE | 4-helical cytokines |
| 292. | KJV57206 | No hit | No hit | 1.ASN_GLYCOSYLATION<br>2.CK2_PHOSPHO_SITE<br>3.MYRISTYL<br>4.PKC_PHOSPHO_SITE<br>5.TYR_PHOSPHO_SITE | 4-helical cytokines |
| 293. | KJV57207 | No hit | dnaA N-terminal domain protein | 1.CK2_PHOSPHO_SITE<br>2.MYRISTYL<br>3.PKC_PHOSPHO_SITE | 4-helical cytokines |
| 294. | KJV57212 | No hit | No hit | 1.ASN_GLYCOSYLATION<br>2.CAMP_PHOSPHO_SITE<br>3.CK2_PHOSPHO_SITE<br>4.MYRISTYL<br>5.PKC_PHOSPHO_SITE | Belta-grasp |
| 295. | KJV57216 | No hit | No hit | 1.ASN_GLYCOSYLATION<br>2.CAMP_PHOSPHO_SITE<br>3.CK2_PHOSPHO_SITE<br>4.PKC_PHOSPHO_SITE<br>5.TYR_PHOSPHO_SITE<br>6.NEBULIN<br>7.DUF2460 | Immunoglobulin-like |
| 296. | KJV57217 | No hit | | 1.ASN_GLYCOSYLATION<br>2.CAMP_PHOSPHO_SITE<br>3.CK2_PHOSPHO_SITE<br>4.PKC_PHOSPHO_SITE<br>5.TYR_PHOSPHO_SITE<br>6.ATP_GTP_A<br>7.LEUCINE_ZIPPER<br>8.EF_HAND_2<br>9.GBD_FH3<br>10.VHS<br>11.C_tripleX<br>12.Octapeptide | 4-helical cytokines |
| 297. | KJV57219 | No hit | No hit | 1.AMIDATION<br>2.ASN_GLYCOSYLATION<br>3.ATP_GTP_A<br>4.CAMP_PHOSPHO_SITE<br>5.CK2_PHOSPHO_SITE<br>6.LEUCINE_ZIPPER | cytochrome c |

| | | | | | |
|---|---|---|---|---|---|
| | | | | 7.PKC_PHOSPHO_SIT<br>8.EF_HAND_2<br>9.GBD_FH3<br>10.C_tripleX<br>11.Octapeptide | |
| 298. | KJV57225 | No hit | **No hit** | 1.MYRISTYL<br>2.PKC_PHOSPHO_SITE<br>3.TYR_PHOSPHO_SITE | DNA-binding 3-helical bundle |
| 299. | KJV57230 | No hit | **Transmembrane region** | 1.ASN_GLYCOSYLATION<br>2.CK2_PHOSPHO_SITE<br>3.LEUCINE_ZIPPER<br>4.MYRISTYL<br>5.PKC_PHOSPHO_SITE | viral coat and capsid proteins |
| 300. | KJV57297 | No hit | **No hit** | 1.ASN_GLYCOSYLATION<br>2.MYRISTYL<br>3.PKC_PHOSPHO_SITE<br>4.HIS_KIN | ferredoxin-like |
| 301. | KJV57301 | No hit | No hit | 1.ASN_GLYCOSYLATION (113-116)<br>2.CK2_PHOSPHO_SITE(67-69)<br>3.MYRISTYL(82-87)<br>4.PKC_PHOSPHO_SITE(68-70) | OB-fold |
| 302. | KJV57311 | No hit | No hit | 1.ASN_GLYCOSYLATION(112-115)<br>2.CK2_PHOSPHO_SITE(71-73)<br>3.MYRISTYL(86-89)<br>4.PKC_PHOSPHO_SITE(63-66) | OB-fold |
| 303. | KJV57315 | No hit | None | 1. CK2_PHOSPHO_SITE<br>2. MYRISTYL<br>3. PKC_PHOSPHO_SITE | OB-fold |
| 304. | KJV57318 | No hit | DNA binding protein, | 1. AMIDATION<br>2. CK2_PHOSPHO_SITE<br>3. MYRISTYL | 4-helical cytokines |
| 305. | KJV57330 | No hit | Chromodomain-Helicase-DNA-binding protein 7 | 1.MYRISTYL<br>2. UPF0546 | 4-helical cytokines |
| 306. | KJV57343 | No hit | No hit | 1.ASN_GLYCOSYLATION<br>2.Bacterial Ig-like domain2 | OB-fold |
| 307. | KJV57346 | PTR2 family proton/oligo peptide symporters | No hit | 1.ASN_GLYCOSYLATION<br>2.CK2_PHOSPHO_SITE<br>3.MYRISTYL<br>3.PKC_PHOSPHO_SITE<br>4.NUMOD3 | OB-fold |
| 308. | KJV57347 | No hit | phage integrase family protein | 1.ASN_GLYCOSYLATION<br>2.CK2_PHOSPHO_SITE<br>3.PKC_PHOSPHO_SITE<br>4.PTR2_2 | immunoglobulin-like |
| 309. | KJV57348 | No hit | EST/SMG like protein | 1. ASN_GLYCOSYLATION<br>2. CK2_PHOSPHO_SITE<br>3. MYRISTYL<br>4. PKC_PHOSPHO_SITE<br>5. Bacterial Ig-like domain2 (<br>6. ZF_THAP | Immunoglobulin-like |
| 310. | KJV57353 | No hit | No hit | 1.ASN_GLYCOSYLATION<br>2.CK2_PHOSPHO_SITE<br>3.MYRISTYL<br>4.PKC_PHOSPHO_SITE<br>5.BIG1<br>6.PXA | 4-helical cytokines |
| 311. | KJV57356 | No hit | No hit | 1.ASN_GLYCOSYLATION<br>2.CK2_PHOSPHO_SITE<br>3.MYRISTYL<br>4.PKC_PHOSPHO_SITE<br>5.BIG1<br>6.PXA | 4-helical cytokines |
| 312. | KJV57360 | No hit | Magnesium Transporter | 1.ASN_GLYCOSYLATION<br>2.CAMP_PHOSPHO_SITE<br>3.CK2_PHOSPHO_SITE<br>4.MYRISTYL<br>5.PKC_PHOSPHO_SITE<br>6.Pumilio RNA-binding repeat profile<br>7.Domain of unknown function | Immunoglobulin-like |

| | | | | | |
|---|---|---|---|---|---|
| 313. | KJV57361 | No hit | Transmembrane region | 1.ASN_GLYCOSYLATION<br>2.CAMP_PHOSPHO_SITE<br>3.CK2_PHOSPHO_SITE<br>4.PKC_PHOSPHO_SITE<br>5.TYR_PHOSPHO_SITE<br>6. BRCA2 repeat | 4-helical up-and-down bundle |
| 314. | KJV57362 | No hit | PA14 domain-containing protein | 1.ASN_GLYCOSYLATION<br>2.CAMP_PHOSPHO_SITE<br>3.CK2_PHOSPHO_SITE<br>4.MYRISTYL<br>5.PKC_PHOSPHO_SITE<br>6.Serine-rich region<br>7.Threonine-rich region<br>8.Fibritin_C<br>Fibritin C-terminal region<br>9.H2-forming N5,N10-methylenetetrahydromethanopterin dehydrogenase | Immunoglobulin-like |
| 315. | KJV57365 | No hit | **No hit** | 1.ASN_GLYCOSYLATION<br>2.MYRISTYL<br>3.PKC_PHOSPHO_SITE<br>4. bacterial Ig-like domain 1 | DNA-binding 3-helical bundle |
| 316. | KJV57366 | No hit | **No hit** | 1.AMIDATION ASN_GLYCOSYLATION<br>2.CK2_PHOSPHO_SITE<br>3.PKC_PHOSPHO_SITE<br>4.TYR_PHOSPHO_SITE | Immunoglobulin-like |
| 317. | KJV57369 | No hit | **toprim domain protein** | 1.CK2_PHOSPHO_SITE<br>2.MYRISTYL | ferredoxin-like |
| 318. | KJV57375 | Serpin signature type protein | | 1.ASN_GLYCOSYLATION<br>2.CAMP_PHOSPHO_SITE<br>3.CK2_PHOSPHO_SITE<br>4.PKC_PHOSPHO_SITE<br>5.SERPIN | 4-helical cytokines |
| 319. | KJV57379 | No hit | **lccl domain containing protein** | 1.ASN_GLYCOSYLATION<br>2.CK2_PHOSPHO_SITE<br>3.MYRISTYL<br>4.PKC_PHOSPHO_SITE<br>5.35_APXCYT<br>6.ASN_RICH<br>7.PPASE_TENSIN<br>8.IpgD | OB-fold |
| 320. | KJV57382 | No hit | **No hit** | EMPTY | DNA-binding 3-helical bundle |
| 321. | KJV57383 | No hit | **No hit** | 1.MYRISTYL<br>2.PKC_PHOSPHO_SITE | OB-fold |
| 322. | KJV57385 | No hit | **PREDICTED: probable L-type lectin-domain containing receptor kinase II.1** | 1.ASN_GLYCOSYLATION<br>2.CAMP_PHOSPHO_SITE<br>3.CK2_PHOSPHO_SITE<br>4.MYRISTYL<br>5.PKC_PHOSPHO_SITE<br>6.TYR_PHOSPHO_SITE<br>7.NEBULIN<br>8.SRR | OB-fold |
| 323. | KJV57393 | No hit | **PREDICTED: ankyrin repeat and BTB/POZ domain-containing protein 2-like** | 1.ASN_GLYCOSYLATION<br>2.CAMP_PHOSPHO_SITE<br>3.CK2_PHOSPHO_SITE<br>4.MYRISTYL<br>5.PKC_PHOSPHO_SITE<br>6.TYR_PHOSPHO_SITE<br>7.RFC<br>8. Ank | immunoglobulin-like |
| 324. | KJV57403 | No hit | **No hit** | 1.CK2_PHOSPHO_SITE<br>2.MYRISTYL | OB-fold |
| 325. | KJV57410 | No hit | **No hit** | 1.CK2_PHOSPHO_SITE<br>2.BIG1<br>3.Phage_holin_6 | 4-helical cytokines |
| 326. | KJV57412 | No hit | **No hit** | 1.PKC_PHOSPHO_SITE | DNA-binding 3-helical bundle |
| 327. | KJV57416 | No hit | **Exonuclease domain protein** | 1.ASN_GLYCOSYLATION<br>2.CAMP_PHOSPHO_SITE<br>3.CK2_PHOSPHO_SITE<br>4.MYRISTYL<br>5.PKC_PHOSPHO_SITE<br>6.TYR_PHOSPHO_SITE | 4-helical cytokines |

| | | | | | |
|---|---|---|---|---|---|
| | | | | 7.C_CAP_COFACTOR_C | |
| 328. | KJV57418 | No hit | **GHKL domain-containing protein** | 1.CK2_PHOSPHO_SITE<br>2.MICROBODIES_CTER<br>3.MYRISTYL<br>4.TYR_PHOSPHO_SITE | 4-helical cytokines |
| 329. | KJV57432 | No hit | **Error** | 1.ASN_GLYCOSYLATION<br>2.MYRISTYL<br>3.PKC_PHOSPHO_SITE<br>4.BIG1 | DNA-binding 3-helical bundle |
| 330. | KJV57451 | No hit | **DUF490 domain-containing protein** | | 4-helical cytokines |
| 331. | KJV57461 | No hit | **No hit** | 1.NLS_BP | DNA-binding 3-helical bundle |
| 332. | KJV57462 | No hit | **No hit** | Empty | 4-helical cytokines |
| 333. | KJV57464 | No hit | **PREDICTED: BRCT domain-containing protein At4g02110-like** | 1.ASN_GLYCOSYLATION<br>2.CAMP_PHOSPHO_SITE<br>3.CK2_PHOSPHO_SITE<br>4.MYRISTYL<br>5.PKC_PHOSPHO_SITE<br>6.TYR_PHOSPHO_SITE<br>7.ASN_RICH<br>8.Cas_APE2256<br>9.PepX_N<br>10.DUF2013<br>11.NUMOD3 | OB-fold |
| 334. | KJV57571 | No hit | **No hit** | 1.ASN_GLYCOSYLATION<br>2.MYRISTYL<br>3.PKC_PHOSPHO_SITE | conA-like lectin/glucanases |
| 335. | KJV57572 | No hit | **MULTISPECIES: FimV domain-containing protein** | 1.CK2_PHOSPHO_SITE<br>2.MYRISTYL<br>3.PKC_PHOSPHO_SITE<br>4.BCAS2<br>5.PAH<br>6.Topoisom_I | immunoglobulin-like |
| 336. | KJV57574 | No hit | **Transmembrane region** | 1.ASN_GLYCOSYLATION<br>2.MICROBODIES_CTER<br>3.MYRISTYL<br>4.PKC_PHOSPHO_SITE<br>5.DUF165 | 4-helical cytokines |
| 337. | KJV57580 | No hit | **No hit** | 1.CK2_PHOSPHO_SITE<br>2.MYRISTYL<br>3.PKC_PHOSPHO_SITE<br>4.BIG1 | OB-fold |
| 338. | KJV57582 | No hit | **No hit** | 1.ASN_GLYCOSYLATION<br>2.CAMP_PHOSPHO_SITE<br>3.CK2_PHOSPHO_SITE<br>4.MYRISTYL<br>5.PKC_PHOSPHO_SITE<br>6.TYR_PHOSPHO_SITE | beta-trefoil |
| 339. | KJV57585 | No hit | **No hit** | 1.MYRISTYL<br>2.PKC_PHOSPHO_SITE<br>3.TYR_PHOSPHO_SITE<br>4.BIG1<br>5.Allatostatin | Cupredoxins |
| 340. | KJV57590 | No hit | **No hit** | Empty | immunoglobulin-like |
| 341. | KJV57610 | No hit | **No hit** | 1.AMIDATION<br>2.MYRISTYL | DNA-binding 3-helical bundle |
| 342. | KJV57616 | No hit | **No hit** | 1.MYRISTYL<br>2.PKC_PHOSPHO_SITE<br>3.TYR_PHOSPHO_SITE | DNA-binding 3-helical bundle |
| 343. | KJV57620 | No hit | **Transmembrane region** | 1.ASN_GLYCOSYLATION<br>2.CK2_PHOSPHO_SITE<br>3.LEUCINE_ZIPPER<br>4.MYRISTYL<br>5.PKC_PHOSPHO_SITE | viral coat and capsid proteins |
| 344. | KJV57626 | No hit | **No hit** | 1.ASN_GLYCOSYLATION<br>2.MYRISTYL<br>3.PKC_PHOSPHO_SITE<br>4.HIS_KIN | ferredoxin-like |

OB-Fold protein= Oligonucleotide/Oligosaccharide-Binding (OB) fold protein.
