# Supplementary material for: In silico characterization of hypothetical proteins from Orientia tsutsugamushi str. Karp uncovers virulence genes
Source: Heliyon. 2019 Nov 1;5(10):e02734. doi: 10.1016/j.heliyon.2019.e02734 (PMC6838952; doi:10.1016/j.heliyon.2019.e02734)
Supplement: Supplementary file 4 [file mmc4.pdf]

S.No	Accession No.	Amino acid Composition based		Dipeptide Composition Based		PSI-BLAST created PSSM Profiles		Higher order Dipeptide Composition Based		Cascade of SVMs and PSI-BLAST		Average Scores
		Results	Scores	Results	Scores	Results	Scores	Results	Scores	Results	Scores	
1.	KJV57131	Virulent	0.9178	Virulent	2.2957	Virulent	1.2206	Virulent	2.6358	Virulent	0.9184	1.59766
2.	KJV57379	Virulent	1.5868	Virulent	1.7882	Virulent	1.4959	Virulent	1.2774	Virulent	1.5373	1.53712
3.	KJV57416	Virulent	1.511	Virulent	1.3622	Virulent	1.5987	Virulent	2.0421	Virulent	0.9692	1.49664
4.	KJV50735	Virulent	1.0381	Virulent	1.8509	Virulent	1.0601	Virulent	2.2208	Virulent	0.8616	1.4063
5.	KJV55958	Virulent	1.0906	Virulent	1.6763	Virulent	1.4196	Virulent	1.8863	Virulent	0.9477	1.4041
6.	KJV50994	Virulent	0.9864	Virulent	1.8571	Virulent	1.0425	Virulent	2.2675	Virulent	0.8448	1.39966
7.	KJV53007	Virulent	0.9266	Virulent	1.8414	Virulent	1.1017	Virulent	2.2594	Virulent	0.8394	1.3937
8.	KJV52681	Virulent	1.4108	Virulent	1.873	Virulent	1.1693	Virulent	1.4896	Virulent	1.0128	1.3911
9.	KJV56935	Virulent	0.9485	Virulent	1.8072	Virulent	1.1589	Virulent	2.1562	Virulent	0.8693	1.38802
10.	KJV54168	Virulent	1.2096	Virulent	1.6667	Virulent	1.2093	Virulent	1.7423	Virulent	1.021	1.36978
11.	KJV53284	Virulent	1.3103	Virulent	1.0041	Virulent	1.2612	Virulent	2.2527	Virulent	0.9769	1.36104
12.	KJV57348	Virulent	0.9076	Virulent	1.772	Virulent	1.4456	Virulent	1.6504	Virulent	0.9341	1.34194
13.	KJV54735	Virulent	1.4234	Virulent	1.5279	Virulent	1.4345	Virulent	1.1387	Virulent	1.085	1.3219
14.	KJV53188	Virulent	1.08	Virulent	1.5717	Virulent	1.1006	Virulent	1.8276	Virulent	1.0163	1.31924
15.	KJV55734	Virulent	0.9327	Virulent	1.1912	Virulent	1.2217	Virulent	2.2464	Virulent	0.963	1.311
16.	KJV53939	Virulent	1.3339	Virulent	1.511	Virulent	1.0233	Virulent	1.551	Virulent	1.0762	1.29908
17.	KJV55659	Virulent	1.2404	Virulent	1.8791	Virulent	0.8758	Virulent	1.4898	Virulent	0.9889	1.2948
18.	KJV54587	Virulent	1.6516	Virulent	1.4787	Virulent	1.2528	Virulent	1.019	Virulent	1.0679	1.294
19.	KJV54139	Virulent	1.4621	Virulent	1.2146	Virulent	1.1112	Virulent	1.51	Virulent	1.1151	1.2826
20.	KJV56583	Virulent	1.3002	Virulent	0.9569	Virulent	1.5489	Virulent	1.4896	Virulent	1.0944	1.278
21.	KJV54508	Virulent	1.3796	Virulent	1.0061	Virulent	1.4037	Virulent	1.439	Virulent	1.1215	1.26998
22.	KJV55874	Virulent	1.3446	Virulent	0.9225	Virulent	1.5398	Virulent	1.3943	Virulent	1.1028	1.2608
23.	KJV55533	Virulent	1.239	Virulent	1.8973	Virulent	0.8824	Virulent	1.2263	Virulent	1.0019	1.24938
24.	KJV56683	Virulent	1.3132	Virulent	1.2083	Virulent	0.9426	Virulent	1.6811	Virulent	1.075	1.24404
25.	KJV57311	Virulent	0.9809	Virulent	1.7789	Virulent	1.1815	Virulent	1.3329	Virulent	0.8875	1.23234
26.	KJV56036	Virulent	1.3556	Virulent	1.8793	Virulent	0.8037	Virulent	1.1186	Virulent	0.9595	1.22334
27.	KJV53935	Virulent	1.3824	Virulent	0.9454	Virulent	1.142	Virulent	1.4598	Virulent	1.1236	1.21064

28.	KJV55746	Virulent	1.0308	Virulent	1.5521	Virulent	0.9978	Virulent	1.373	Virulent	1.0921	1.20916
29.	KJV56143	Virulent	1.1156	Virulent	1.0511	Virulent	1.1646	Virulent	1.5631	Virulent	1.1072	1.20032
30.	KJV57393	Virulent	0.9855	Virulent	1.159	Virulent	1.2236	Virulent	1.5162	Virulent	1.0911	1.19508
31.	KJV52864	Virulent	1.2226	Virulent	1.4901	Virulent	1.0702	Virulent	1.0677	Virulent	1.102	1.19052
32.	KJV57120	Virulent	1.4178	Virulent	0.9323	Virulent	1.4383	Virulent	0.967	Virulent	1.0375	1.15858
33.	KJV54670	Virulent	1.1082	Virulent	1.3983	Virulent	0.9956	Virulent	1.1537	Virulent	1.1142	1.154
34.	KJV57203	Virulent	1.023	Virulent	1.0318	Virulent	1.0671	Virulent	1.5494	Virulent	1.0971	1.15368
35.	KJV56211	Virulent	1.086	Virulent	1.2792	Virulent	1.08	Virulent	1.1946	Virulent	1.124	1.15276
36.	KJV51002	Virulent	1.1881	Virulent	1.1188	Virulent	1.0868	Virulent	1.2358	Virulent	1.1316	1.15222
37.	KJV56684	Virulent	1.0339	Virulent	0.9725	Virulent	1.195	Virulent	1.4313	Virulent	1.1025	1.14704
38.	KJV50818	Virulent	1.2394	Virulent	1.081	Virulent	0.9281	Virulent	1.2893	Virulent	1.1164	1.13084
39.	KJV53916	Virulent	1.1855	Virulent	0.9014	Virulent	1.0891	Virulent	1.3351	Virulent	1.1172	1.12566
40.	KJV52751	Virulent	1.2581	Virulent	0.6884	Virulent	1.3906	Virulent	1.1715	Virulent	1.0925	1.12022
41.	KJV57626	Virulent	1.1033	Virulent	0.9177	Virulent	1.2272	Virulent	1.104	Virulent	1.0994	1.09032
42.	KJV50787	Virulent	0.821	Virulent	1.134	Virulent	0.8971	Virulent	1.4924	Virulent	1.0829	1.08548
43.	KJV54906	Virulent	1.1008	Virulent	1.138	Virulent	0.9251	Virulent	1.1233	Virulent	1.1113	1.0797
44.	KJV57212	Virulent	0.736	Virulent	1.3844	Virulent	0.7176	Virulent	1.4391	Virulent	1.0767	1.07076
45.	KJV57216	Virulent	1.199	Virulent	1.4498	Virulent	1.3006	Virulent	0.4069	Virulent	0.9915	1.06956
46.	KJV52478	Virulent	1.1052	Virulent	0.6497	Virulent	1.2606	Virulent	1.2378	Virulent	1.082	1.06706
47.	KJV56675	Virulent	0.9893	Virulent	1.0009	Virulent	0.7595	Virulent	1.507	Virulent	1.0611	1.06356
48.	KJV50671	Virulent	1.1341	Virulent	0.8286	Virulent	1.0583	Virulent	1.1183	Virulent	1.0939	1.04664
49.	KJV54785	Virulent	0.9488	Virulent	0.9501	Virulent	0.9662	Virulent	1.2497	Virulent	1.0915	1.04126
50.	KJV55465	Virulent	1.2074	Virulent	0.6517	Virulent	0.8766	Virulent	1.4166	Virulent	1.0525	1.04096
51.	KJV52046	Virulent	1.125	Virulent	0.4258	Virulent	1.0812	Virulent	1.5058	Virulent	1.0433	1.03622
52.	KJV53129	Virulent	1.3451	Virulent	0.9869	Virulent	1.2255	Virulent	0.5632	Virulent	1.055	1.03514
53.	KJV54170	Virulent	1.0829	Virulent	0.9067	Virulent	1.0249	Virulent	1.0456	Virulent	1.0894	1.0299
54.	KJV57117	Virulent	1.2103	Virulent	1.004	Virulent	1.1659	Virulent	0.6582	Virulent	1.0633	1.02034
55.	KJV57230	Virulent	0.7618	Virulent	1.4071	Virulent	0.9733	Virulent	0.8748	Virulent	1.0668	1.01676
56.	KJV57144	Virulent	0.7842	Virulent	0.9406	Virulent	0.8525	Virulent	1.4167	Virulent	1.0648	1.01176
57.	KJV56401	Virulent	1.3022	Virulent	1.3618	Virulent	0.821	Virulent	0.5723	Virulent	0.9869	1.00884
58.	KJV54671	Virulent	1.4225	Virulent	0.7659	Virulent	0.9325	Virulent	0.8532	Virulent	1.0672	1.00826
59.	KJV51409	Virulent	0.9735	Virulent	0.8943	Virulent	1.05	Virulent	1.0433	Virulent	1.0738	1.00698

60.	KJV57217	Virulent	1.0185	Virulent	0.8513	Virulent	0.9405	Virulent	1.1414	Virulent	1.0786	1.00606
61.	KJV53065	Virulent	0.9979	Virulent	0.7991	Virulent	0.9951	Virulent	1.1638	Virulent	1.0733	1.00584
62.	KJV54489	Virulent	0.9321	Virulent	1.2993	Virulent	0.7014	Virulent	1.0264	Virulent	1.069	1.00564
63.	KJV56570	Virulent	0.8126	Virulent	1.35	Virulent	0.9879	Virulent	0.7792	Virulent	1.0559	0.99712
64.	KJV54529	Virulent	1.1278	Virulent	0.5458	Virulent	1.2207	Virulent	1.0201	Virulent	1.0577	0.99442
65.	KJV56673	Virulent	1.1365	Virulent	0.7685	Virulent	1.1447	Virulent	0.7944	Virulent	1.0521	0.97924
66.	KJV51205	Virulent	0.9906	Virulent	0.7121	Virulent	1.0368	Virulent	1.0913	Virulent	1.0558	0.97732
67.	KJV53068	Virulent	0.6477	Virulent	0.9665	Virulent	0.881	Virulent	1.3241	Virulent	1.0631	0.97648
68.	KJV51134	Virulent	1.0823	Virulent	0.9394	Virulent	0.79	Virulent	0.9757	Virulent	1.0627	0.97002
69.	KJV57225	Virulent	0.6233	Virulent	1.2356	Virulent	1.0152	Virulent	0.899	Virulent	1.0586	0.96634
70.	KJV57330	Virulent	0.6367	Virulent	0.7327	Virulent	0.8716	Virulent	1.5829	Virulent	0.997	0.96418
71.	KJV55035	Virulent	1.287	Virulent	0.3833	Virulent	1.2092	Virulent	0.8951	Virulent	1.0424	0.9634
72.	KJV50815	Virulent	0.8807	Virulent	0.4889	Virulent	1.0878	Virulent	1.3247	Virulent	1.0296	0.96234
73.	KJV55885	Virulent	1.0173	Virulent	1.6749	Virulent	0.7524	Virulent	0.4647	Virulent	0.8919	0.96024
74.	KJV52749	Virulent	1.2862	Virulent	0.5955	Virulent	0.9974	Virulent	0.8503	Virulent	1.0502	0.95592
75.	KJV55474	Virulent	1.0033	Virulent	0.7473	Virulent	0.9026	Virulent	1.0635	Virulent	1.052	0.95374
76.	KJV52048	Virulent	1.0586	Virulent	0.8171	Virulent	0.7957	Virulent	1.0362	Virulent	1.0532	0.95216
77.	KJV53125	Virulent	0.9876	Virulent	1.1251	Virulent	0.7898	Virulent	0.7973	Virulent	1.0496	0.94988
78.	KJV57347	Virulent	1.2314	Virulent	0.9564	Virulent	0.9914	Virulent	0.5197	Virulent	1.0447	0.94872
79.	KJV56783	Virulent	1.1607	Virulent	0.6007	Virulent	1.4688	Virulent	0.4946	Virulent	1.0117	0.9473
80.	KJV54616	Virulent	0.8321	Virulent	1.3414	Virulent	0.669	Virulent	0.8055	Virulent	1.0324	0.93608
81.	KJV53914	Virulent	1.298	Virulent	0.6545	Virulent	0.9257	Virulent	0.7527	Virulent	1.0447	0.93512
82.	KJV54779	Virulent	1.0309	Virulent	0.7733	Virulent	0.8376	Virulent	0.9628	Virulent	1.0438	0.92968
83.	KJV54492	Virulent	0.8392	Virulent	0.6928	Virulent	1.1458	Virulent	0.9527	Virulent	1.0158	0.92926
84.	KJV54582	Virulent	1.0547	Virulent	0.6405	Virulent	1.0622	Virulent	0.84	Virulent	1.0304	0.92556
85.	KJV54666	Virulent	1.1241	Virulent	0.9135	Virulent	0.9737	Virulent	0.564	Virulent	1.0377	0.9226
86.	KJV56404	Virulent	1.1995	Virulent	0.962	Virulent	0.8933	Virulent	0.5119	Virulent	1.0326	0.91986
87.	KJV55597	Virulent	1.1909	Virulent	0.1939	Virulent	0.8132	Virulent	1.4374	Virulent	0.9511	0.9173
88.	KJV50999	Virulent	1.0279	Virulent	0.8713	Virulent	0.9911	Virulent	0.6442	Virulent	1.0311	0.91312
89.	KJV54506	Virulent	1.1145	Virulent	0.4227	Virulent	0.9155	Virulent	1.0755	Virulent	1.0251	0.91066
90.	KJV57204	Virulent	1.11	Virulent	0.5048	Virulent	0.8954	Virulent	1.0147	Virulent	1.0279	0.91056
91.	KJV55806	Virulent	0.5881	Virulent	0.842	Virulent	1.0315	Virulent	1.0672	Virulent	1.0239	0.91054

92.	KJV57343	Virulent	0.9859	Virulent	0.5843	Virulent	1.0189	Virulent	0.9309	Virulent	1.0225	0.9085
93.	KJV55027	Virulent	1.1972	Virulent	0.7639	Virulent	0.778	Virulent	0.753	Virulent	1.0306	0.90454
94.	KJV52928	Virulent	0.7029	Virulent	1.1447	Virulent	0.4543	Virulent	1.166	Virulent	1.0397	0.90152
95.	KJV55079	Virulent	0.8054	Virulent	1.2172	Virulent	1.0189	Virulent	0.4379	Virulent	1.0103	0.89794
96.	KJV55680	Virulent	0.9905	Virulent	0.5723	Virulent	0.9077	Virulent	0.9934	Virulent	1.0212	0.89702
97.	KJV56930	Virulent	0.9072	Virulent	0.651	Virulent	1.0838	Virulent	0.8339	Virulent	1.0086	0.8969
98.	KJV55220	Virulent	1.1643	Virulent	0.7067	Virulent	0.8317	Virulent	0.7463	Virulent	1.0297	0.89574
99.	KJV55033	Virulent	1.3498	Virulent	0.6582	Virulent	0.6954	Virulent	0.7524	Virulent	1.015	0.89416
100.	KJV51579	Virulent	0.9531	Virulent	1.0089	Virulent	0.6436	Virulent	0.84	Virulent	1.025	0.89412
101.	KJV52376	Virulent	0.8042	Virulent	0.6618	Virulent	1.4457	Virulent	0.5632	Virulent	0.9883	0.89264
102.	KJV53634	Virulent	1.0679	Virulent	1.4053	Virulent	0.1297	Virulent	1.1949	Virulent	0.6459	0.88874
103.	KJV50970	Virulent	0.9413	Virulent	0.9651	Virulent	0.5765	Virulent	0.9394	Virulent	1.0186	0.88818
104.	KJV51003	Virulent	0.902	Virulent	0.9027	Virulent	0.755	Virulent	0.8358	Virulent	1.0328	0.88566
105.	KJV56669	Virulent	0.902	Virulent	0.9027	Virulent	0.755	Virulent	0.8358	Virulent	1.0328	0.88566
106.	KJV57301	Virulent	1.1	Virulent	0.5113	Virulent	0.8318	Virulent	0.9625	Virulent	1.0178	0.88468
107.	KJV54368	Virulent	0.7699	Virulent	0.6969	Virulent	0.7805	Virulent	1.1512	Virulent	1.0228	0.88426
108.	KJV56305	Virulent	1.1035	Virulent	0.8505	Virulent	0.781	Virulent	0.6547	Virulent	1.022	0.88234
109.	KJV50645	Virulent	0.8531	Virulent	0.969	Virulent	0.634	Virulent	0.9403	Virulent	1.0129	0.88186
110.	KJV57129	Virulent	0.8714	Virulent	0.6887	Virulent	0.8139	Virulent	0.9648	Virulent	1.0167	0.8711
111.	KJV55957	Virulent	0.7826	Virulent	0.8789	Virulent	0.5311	Virulent	1.1291	Virulent	1.0277	0.86988
112.	KJV52426	Virulent	0.9641	Virulent	0.6918	Virulent	0.8574	Virulent	0.8131	Virulent	1.0138	0.86804
113.	KJV57297	Virulent	0.9001	Virulent	0.8163	Virulent	0.4929	Virulent	1.1335	Virulent	0.9932	0.8672
114.	KJV53715	Virulent	1.1024	Virulent	0.629	Virulent	0.9416	Virulent	0.6392	Virulent	1.0168	0.8658
115.	KJV54829	Virulent	1.4474	Virulent	0.6592	Virulent	0.1338	Virulent	1.4757	Virulent	0.5728	0.85778
116.	KJV52319	Virulent	0.7764	Virulent	0.9531	Virulent	0.8673	Virulent	0.6549	Virulent	1.0184	0.85402
117.	KJV55217	Virulent	1.2416	Virulent	0.5426	Virulent	0.7111	Virulent	0.7541	Virulent	1.011	0.85208
118.	KJV55225	Virulent	1.02	Virulent	1.5233	Virulent	0.1867	Virulent	0.8614	Virulent	0.6433	0.84694
119.	KJV50518	Virulent	0.8094	Virulent	0.8758	Virulent	0.5977	Virulent	0.9215	Virulent	1.0285	0.84658
120.	KJV56573	Virulent	0.52	Virulent	1.6472	Virulent	0.686	Virulent	0.4323	Virulent	0.9185	0.8408
121.	KJV53187	Virulent	0.8236	Virulent	1.3751	Virulent	0.7407	Virulent	0.3057	Virulent	0.9203	0.83308
122.	KJV51286	Virulent	1.0977	Virulent	0.6447	Virulent	0.6003	Virulent	0.8234	Virulent	0.9957	0.83236
123.	KJV56304	Virulent	0.6164	Virulent	1.1613	Virulent	0.7722	Virulent	0.5509	Virulent	1.0256	0.82528

124.	KJV56575	Virulent	0.9216	Virulent	0.5246	Virulent	1.0089	Virulent	0.6751	Virulent	0.9893	0.8239
125.	KJV57572	Virulent	0.7561	Virulent	0.6545	Virulent	0.7722	Virulent	0.9164	Virulent	1.0048	0.8208
126.	KJV51375	Virulent	0.7954	Virulent	0.6636	Virulent	0.818	Virulent	0.8259	Virulent	0.9988	0.82034
127.	KJV54614	Virulent	0.6905	Virulent	0.5106	Virulent	0.7748	Virulent	1.0767	Virulent	0.9922	0.80896
128.	KJV57620	Virulent	1.2588	Virulent	0.4917	Virulent	0.9604	Virulent	0.315	Virulent	1.0121	0.8076
129.	KJV55951	Virulent	0.939	Virulent	0.5548	Virulent	0.8242	Virulent	0.7244	Virulent	0.9936	0.8072
130.	KJV54539	Virulent	0.9298	Virulent	0.5183	Virulent	0.8757	Virulent	0.705	Virulent	0.99	0.80376
131.	KJV51411	Virulent	0.9879	Virulent	0.6089	Virulent	0.7208	Virulent	0.7008	Virulent	0.9997	0.80362
132.	KJV51373	Virulent	0.7793	Virulent	0.4851	Virulent	1.0874	Virulent	0.6864	Virulent	0.977	0.80304
133.	KJV53067	Virulent	1.0303	Virulent	0.603	Virulent	0.8638	Virulent	0.5135	Virulent	1.0028	0.80268
134.	KJV57616	Virulent	1.3041	Virulent	0.4373	Virulent	0.4613	Virulent	0.8589	Virulent	0.9474	0.8018
135.	KJV57574	Virulent	0.7623	Virulent	0.8122	Virulent	0.3106	Virulent	1.1724	Virulent	0.9506	0.80162
136.	KJV51695	Virulent	0.9425	Virulent	0.548	Virulent	0.7824	Virulent	0.7397	Virulent	0.9944	0.8014
137.	KJV51131	Virulent	1.0349	Virulent	0.6062	Virulent	0.7729	Virulent	0.5864	Virulent	1.0023	0.80054
138.	KJV51776	Virulent	1.1469	Virulent	0.5828	Virulent	0.6833	Virulent	0.5698	Virulent	0.9998	0.79652
139.	KJV50672	Virulent	0.4181	Virulent	1.0618	Virulent	0.7467	Virulent	0.6657	Virulent	1.0822	0.7949
140.	KJV51142	Virulent	0.5182	Virulent	1.569	Virulent	0.6371	Virulent	0.3472	Virulent	0.8907	0.79244
141.	KJV55165	Virulent	0.9985	Virulent	0.5513	Virulent	0.5778	Virulent	0.8316	Virulent	0.9922	0.79028
142.	KJV51289	Virulent	1.0134	Virulent	0.5219	Virulent	0.7677	Virulent	0.6494	Virulent	0.9984	0.79016
143.	KJV56131	Virulent	0.9078	Virulent	0.6112	Virulent	0.6365	Virulent	0.7805	Virulent	0.9984	0.78688
144.	KJV53363	Virulent	1.1014	Virulent	0.4208	Virulent	0.5661	Virulent	0.8567	Virulent	0.985	0.786
145.	KJV52230	Virulent	0.7584	Virulent	0.6654	Virulent	0.7284	Virulent	0.7611	Virulent	1.0013	0.78292
146.	KJV57382	Virulent	0.9106	Virulent	0.5433	Virulent	0.7099	Virulent	0.7504	Virulent	0.9942	0.78168
147.	KJV51784	Virulent	0.8297	Virulent	0.529	Virulent	0.7885	Virulent	0.7629	Virulent	0.9872	0.77946
148.	KJV56045	Virulent	0.7706	Virulent	1.1445	Virulent	0.2205	Virulent	0.716	Virulent	1.0419	0.7787
149.	KJV54466	Virulent	0.8766	Virulent	0.6846	Virulent	0.5316	Virulent	0.8013	Virulent	0.999	0.77862
150.	KJV57464	Virulent	0.6901	Virulent	0.7889	Virulent	1.1364	Virulent	0.2719	Virulent	1.0039	0.77824
151.	KJV54909	Virulent	1.0193	Virulent	0.5082	Virulent	0.7028	Virulent	0.6544	Virulent	0.9993	0.7768
152.	KJV54878	Virulent	1.1135	Virulent	0.4763	Virulent	0.6518	Virulent	0.6368	Virulent	1.0033	0.77634
153.	KJV56219	Virulent	0.7531	Virulent	0.5711	Virulent	0.6763	Virulent	0.8653	Virulent	1.0001	0.77318
154.	KJV50625	Virulent	0.7428	Virulent	1.0544	Virulent	0.8106	Virulent	0.2734	Virulent	0.9811	0.77246
155.	KJV53073	Virulent	0.6411	Virulent	0.9776	Virulent	0.3096	Virulent	0.9285	Virulent	1.0038	0.77212

156.	KJV54370	Virulent	0.8656	Virulent	0.4961	Virulent	0.5718	Virulent	0.928	Virulent	0.9912	0.77054
157.	KJV56137	Virulent	0.7524	Virulent	0.5559	Virulent	0.7453	Virulent	0.7962	Virulent	0.9923	0.76842
158.	KJV50906	Virulent	0.743	Virulent	0.7097	Virulent	0.4801	Virulent	0.88	Virulent	1.0177	0.7661
159.	KJV54141	Virulent	0.6432	Virulent	0.8378	Virulent	0.6612	Virulent	0.6747	Virulent	1.0078	0.76494
160.	KJV56300	Virulent	0.9703	Virulent	0.4857	Virulent	0.6369	Virulent	0.7325	Virulent	0.9971	0.7645
161.	KJV53524	Virulent	0.7462	Virulent	0.6983	Virulent	0.5106	Virulent	0.8474	Virulent	1.0182	0.76414
162.	KJV54528	Virulent	0.8333	Virulent	0.4988	Virulent	0.8146	Virulent	0.6826	Virulent	0.9843	0.76272
163.	KJV57346	Virulent	0.8224	Virulent	0.8043	Virulent	0.5905	Virulent	0.5893	Virulent	0.9982	0.76094
164.	KJV57375	Virulent	0.9004	Virulent	0.4866	Virulent	0.6	Virulent	0.8114	Virulent	0.9956	0.7588
165.	KJV55810	Virulent	0.901	Virulent	0.5294	Virulent	0.6833	Virulent	0.6806	Virulent	0.9958	0.75802
166.	KJV57360	Virulent	0.7304	Virulent	0.5375	Virulent	0.8253	Virulent	0.709	Virulent	0.9836	0.75716
167.	KJV56402	Virulent	0.7785	Virulent	0.6107	Virulent	0.725	Virulent	0.6723	Virulent	0.9955	0.7564
168.	KJV57410	Virulent	0.894	Virulent	0.5068	Virulent	0.7138	Virulent	0.6669	Virulent	0.9938	0.75506
169.	KJV57207	Virulent	0.7557	Virulent	0.5648	Virulent	0.641	Virulent	0.8039	Virulent	1.0034	0.75376
170.	KJV50624	Virulent	1.0036	Virulent	0.5065	Virulent	0.652	Virulent	0.5997	Virulent	1.0018	0.75272
171.	KJV55871	Virulent	1.0036	Virulent	0.5065	Virulent	0.652	Virulent	0.5997	Virulent	1.0018	0.75272
172.	KJV55462	Virulent	0.7365	Virulent	0.6224	Virulent	0.5285	Virulent	0.8566	Virulent	1.0167	0.75214
173.	KJV51253	Virulent	0.6137	Virulent	1.0495	Virulent	0.6912	Virulent	0.4036	Virulent	1.0016	0.75192
174.	KJV57318	Virulent	0.799	Virulent	0.5657	Virulent	0.5149	Virulent	0.8582	Virulent	1.0057	0.7487
175.	KJV50939	Virulent	0.7247	Virulent	0.6108	Virulent	0.5744	Virulent	0.8171	Virulent	1.016	0.7486
176.	KJV55231	Virulent	0.7547	Virulent	0.4567	Virulent	0.8245	Virulent	0.7203	Virulent	0.9815	0.74754
177.	KJV54975	Virulent	0.977	Virulent	0.4234	Virulent	0.3428	Virulent	1.0731	Virulent	0.9203	0.74732
178.	KJV51881	Virulent	0.6308	Virulent	0.6393	Virulent	0.4143	Virulent	1.0051	Virulent	1.0299	0.74388
179.	KJV54413	Virulent	0.9003	Virulent	0.4912	Virulent	0.5814	Virulent	0.7241	Virulent	1.0017	0.73974
180.	KJV56132	Virulent	0.8884	Virulent	0.5527	Virulent	0.401	Virulent	0.8677	Virulent	0.9848	0.73892
181.	KJV56040	Virulent	0.6171	Virulent	0.608	Virulent	0.7035	Virulent	0.7452	Virulent	1.015	0.73776
182.	KJV55293	Virulent	0.8169	Virulent	0.6034	Virulent	0.5367	Virulent	0.7219	Virulent	1.0088	0.73754
183.	KJV56296	Virulent	0.8162	Virulent	0.8142	Virulent	0.5291	Virulent	0.5305	Virulent	0.9891	0.73582
184.	KJV55336	Virulent	0.1736	Virulent	0.879	Virulent	1.0301	Virulent	0.5064	Virulent	1.087	0.73522
185.	KJV53203	Virulent	0.8462	Virulent	0.3987	Virulent	0.9782	Virulent	0.465	Virulent	0.9875	0.73512
186.	KJV54147	Virulent	0.8086	Virulent	0.5286	Virulent	0.4716	Virulent	0.855	Virulent	1.0055	0.73386
187.	KJV54783	Virulent	0.6488	Virulent	0.8354	Virulent	0.4707	Virulent	0.6686	Virulent	1.0343	0.73156

188.	KJV52869	Virulent	0.732	Virulent	0.5539	Virulent	0.6409	Virulent	0.7096	Virulent	1.0079	0.72886
189.	KJV54978	Virulent	0.7174	Virulent	0.473	Virulent	0.801	Virulent	0.6667	Virulent	0.9862	0.72886
190.	KJV51076	Virulent	0.7002	Virulent	0.6851	Virulent	0.4564	Virulent	0.7627	Virulent	1.0313	0.72714
191.	KJV54438	Virulent	1.023	Virulent	0.4578	Virulent	0.813	Virulent	0.3198	Virulent	1.0037	0.72346
192.	KJV54877	Virulent	0.6008	Virulent	0.4376	Virulent	0.9689	Virulent	0.6264	Virulent	0.9834	0.72342
193.	KJV51080	Virulent	0.6671	Virulent	0.4818	Virulent	0.7647	Virulent	0.7065	Virulent	0.9936	0.72274
194.	KJV50707	Virulent	0.7779	Virulent	0.9682	Virulent	0.4927	Virulent	0.4243	Virulent	0.948	0.72222
195.	KJV57610	Virulent	0.7729	Virulent	0.5155	Virulent	0.7542	Virulent	0.5669	Virulent	0.9935	0.7206
196.	KJV53189	Virulent	0.6727	Virulent	0.5181	Virulent	0.6708	Virulent	0.7293	Virulent	1.0088	0.71994
197.	KJV57571	Virulent	0.7504	Virulent	0.5164	Virulent	0.6425	Virulent	0.6831	Virulent	1.0068	0.71984
198.	KJV57369	Virulent	0.7221	Virulent	0.4986	Virulent	0.6746	Virulent	0.6965	Virulent	1.0036	0.71908
199.	KJV50905	Virulent	0.9629	Virulent	0.4649	Virulent	0.637	Virulent	0.5277	Virulent	1.0021	0.71892
200.	KJV51578	Virulent	0.9629	Virulent	0.4649	Virulent	0.637	Virulent	0.5277	Virulent	1.0021	0.71892
201.	KJV54946	Virulent	0.6383	Virulent	0.4709	Virulent	0.6783	Virulent	0.7968	Virulent	1.0077	0.7184
202.	KJV57585	Virulent	1.0586	Virulent	0.4849	Virulent	0.4015	Virulent	0.6582	Virulent	0.9885	0.71834
203.	KJV53004	Virulent	0.9135	Virulent	0.5602	Virulent	0.4192	Virulent	0.6862	Virulent	1	0.71582
204.	KJV56574	Virulent	0.7533	Virulent	0.8978	Virulent	0.9113	Virulent	0.5461	Virulent	0.4659	0.71488
205.	KJV55882	Virulent	0.3856	Virulent	0.7203	Virulent	0.8847	Virulent	0.5224	Virulent	1.0492	0.71244
206.	KJV52234	Virulent	0.8616	Virulent	0.8004	Virulent	0.7968	Virulent	0.1241	Virulent	0.9766	0.7119
207.	KJV51004	Virulent	0.714	Virulent	0.5851	Virulent	0.5698	Virulent	0.6619	Virulent	1.0236	0.71088
208.	KJV56690	Virulent	0.714	Virulent	0.5851	Virulent	0.5698	Virulent	0.6619	Virulent	1.0236	0.71088
209.	KJV51964	Virulent	0.6895	Virulent	0.5464	Virulent	0.5377	Virulent	0.7404	Virulent	1.0304	0.70888
210.	KJV55290	Virulent	0.8422	Virulent	0.5018	Virulent	0.8326	Virulent	0.3594	Virulent	0.9963	0.70646
211.	KJV51788	Virulent	0.4941	Virulent	0.4439	Virulent	0.5535	Virulent	1.0058	Virulent	1.034	0.70626
212.	KJV54701	Virulent	0.7338	Virulent	0.4873	Virulent	0.6421	Virulent	0.6529	Virulent	1.0109	0.7054
213.	KJV54167	Virulent	1.0921	Virulent	0.3064	Virulent	0.7108	Virulent	0.4017	Virulent	1.0139	0.70498
214.	KJV52428	Virulent	0.7151	Virulent	0.5406	Virulent	0.5227	Virulent	0.7118	Virulent	1.0302	0.70408
215.	KJV56688	Virulent	0.7979	Virulent	0.5345	Virulent	0.4941	Virulent	0.6706	Virulent	1.0227	0.70396
216.	KJV56142	Virulent	0.7227	Virulent	0.496	Virulent	0.7691	Virulent	0.5315	Virulent	0.9966	0.70318
217.	KJV53919	Virulent	0.729	Virulent	0.5384	Virulent	0.5544	Virulent	0.6685	Virulent	1.0253	0.70312
218.	KJV54459	Virulent	0.6099	Virulent	0.5621	Virulent	0.6674	Virulent	0.6514	Virulent	1.024	0.70296
219.	KJV54463	Virulent	0.6834	Virulent	0.5415	Virulent	0.5767	Virulent	0.6852	Virulent	1.0279	0.70294

220.	KJV53716	Virulent	0.5598	Virulent	0.9746	Virulent	0.4373	Virulent	0.5275	Virulent	1.0149	0.70282
221.	KJV55751	Virulent	0.8029	Virulent	0.4923	Virulent	0.5232	Virulent	0.6695	Virulent	1.0219	0.70196
222.	KJV55037	Virulent	0.7243	Virulent	0.5184	Virulent	0.5666	Virulent	0.6677	Virulent	1.0248	0.70036
223.	KJV57383	Virulent	0.8073	Virulent	0.4691	Virulent	0.7072	Virulent	0.5128	Virulent	1.0037	0.70002
224.	KJV55962	Virulent	0.6996	Virulent	0.5936	Virulent	0.5488	Virulent	0.6163	Virulent	1.0313	0.69792
225.	KJV54581	Virulent	0.639	Virulent	0.489	Virulent	0.6896	Virulent	0.6521	Virulent	1.0138	0.6967
226.	KJV52143	Virulent	0.7129	Virulent	0.9379	Virulent	0.349	Virulent	0.5257	Virulent	0.948	0.6947
227.	KJV54789	Virulent	0.6578	Virulent	0.488	Virulent	0.6272	Virulent	0.6657	Virulent	1.0239	0.69252
228.	KJV53820	Virulent	0.6862	Virulent	0.5136	Virulent	0.4832	Virulent	0.7363	Virulent	1.0421	0.69228
229.	KJV54143	Virulent	0.8836	Virulent	0.5132	Virulent	0.3416	Virulent	0.7141	Virulent	1.0056	0.69162
230.	KJV55344	Virulent	0.8836	Virulent	0.5132	Virulent	0.3416	Virulent	0.7141	Virulent	1.0056	0.69162
231.	KJV54398	Virulent	0.7849	Virulent	0.5319	Virulent	0.4329	Virulent	0.6691	Virulent	1.0319	0.69014
232.	KJV55458	Virulent	0.952	Virulent	0.4461	Virulent	0.3565	Virulent	0.6854	Virulent	1.008	0.6896
233.	KJV55409	Virulent	0.6905	Virulent	0.489	Virulent	0.5618	Virulent	0.6672	Virulent	1.0331	0.68832
234.	KJV55535	Virulent	0.6924	Virulent	0.4843	Virulent	0.535	Virulent	0.681	Virulent	1.0377	0.68608
235.	KJV54488	Virulent	0.9103	Virulent	0.3578	Virulent	0.5268	Virulent	0.6047	Virulent	1.0292	0.68576
236.	KJV54527	Virulent	0.669	Virulent	0.5369	Virulent	0.5107	Virulent	0.6625	Virulent	1.0463	0.68508
237.	KJV54913	Virulent	0.6693	Virulent	0.5064	Virulent	0.514	Virulent	0.6826	Virulent	1.0457	0.6836
238.	KJV54556	Virulent	0.6609	Virulent	0.5164	Virulent	0.5075	Virulent	0.6758	Virulent	1.049	0.68192
239.	KJV53937	Virulent	0.5789	Virulent	0.6745	Virulent	0.3561	Virulent	0.7189	Virulent	1.0745	0.68058
240.	KJV54707	Virulent	0.6956	Virulent	0.4878	Virulent	0.513	Virulent	0.6597	Virulent	1.0438	0.67998
241.	KJV52571	Virulent	0.6637	Virulent	0.4882	Virulent	0.5677	Virulent	0.6411	Virulent	1.039	0.67994
242.	KJV55294	Virulent	0.6637	Virulent	0.4882	Virulent	0.5677	Virulent	0.6411	Virulent	1.039	0.67994
243.	KJV50907	Virulent	0.615	Virulent	0.5386	Virulent	0.508	Virulent	0.6725	Virulent	1.0592	0.67866
244.	KJV57403	Virulent	0.512	Virulent	0.4973	Virulent	0.6788	Virulent	0.6585	Virulent	1.0426	0.67784
245.	KJV57461	Virulent	0.6995	Virulent	0.5099	Virulent	0.4721	Virulent	0.6547	Virulent	1.0499	0.67722
246.	KJV52477	Virulent	0.6502	Virulent	0.4977	Virulent	0.5127	Virulent	0.6726	Virulent	1.0518	0.677
247.	KJV54540	Virulent	0.5922	Virulent	0.4927	Virulent	0.6321	Virulent	0.6241	Virulent	1.0383	0.67588
248.	KJV51035	Virulent	0.7665	Virulent	0.4302	Virulent	0.533	Virulent	0.6117	Virulent	1.0371	0.6757
249.	KJV54874	Virulent	0.7483	Virulent	0.4376	Virulent	0.4982	Virulent	0.6436	Virulent	1.0432	0.67418
250.	KJV55666	Virulent	0.6812	Virulent	0.4976	Virulent	0.486	Virulent	0.6535	Virulent	1.0526	0.67418
251.	KJV55222	Virulent	0.6356	Virulent	0.4536	Virulent	0.5669	Virulent	0.6702	Virulent	1.0438	0.67402

252.	KJV52373	Virulent	0.6602	Virulent	0.4974	Virulent	0.4793	Virulent	0.6699	Virulent	1.0573	0.67282
253.	KJV55667	Virulent	0.7185	Virulent	0.5157	Virulent	0.4418	Virulent	0.6322	Virulent	1.0523	0.6721
254.	KJV54908	Virulent	0.6541	Virulent	0.4905	Virulent	0.5134	Virulent	0.6446	Virulent	1.054	0.67132
255.	KJV56037	Virulent	0.6159	Virulent	0.4984	Virulent	0.5216	Virulent	0.6513	Virulent	1.0597	0.66938
256.	KJV57580	Virulent	0.3522	Virulent	0.2488	Virulent	1.0968	Virulent	0.5899	Virulent	1.0579	0.66912
257.	KJV52144	Virulent	0.6639	Virulent	0.4904	Virulent	0.4717	Virulent	0.6596	Virulent	1.0599	0.6691
258.	KJV51880	Virulent	0.7694	Virulent	0.7443	Virulent	0.6776	Virulent	0.1659	Virulent	0.9776	0.66696
259.	KJV57590	Virulent	0.6499	Virulent	0.4866	Virulent	0.4691	Virulent	0.6627	Virulent	1.0639	0.66644
260.	KJV54341	Virulent	0.6339	Virulent	0.5412	Virulent	0.4571	Virulent	0.6308	Virulent	1.0684	0.66628
261.	KJV57418	Virulent	0.7408	Virulent	0.4969	Virulent	0.4723	Virulent	0.5657	Virulent	1.0506	0.66526
262.	KJV57366	Virulent	0.8043	Virulent	0.5223	Virulent	0.4247	Virulent	0.5319	Virulent	1.0413	0.6649
263.	KJV55080	Virulent	0.6686	Virulent	0.5276	Virulent	0.3795	Virulent	0.6768	Virulent	1.0679	0.66408
264.	KJV51919	Virulent	1.016	Virulent	0.3996	Virulent	0.2745	Virulent	0.614	Virulent	1.0045	0.66172
265.	KJV54465	Virulent	0.6341	Virulent	0.4891	Virulent	0.4402	Virulent	0.6551	Virulent	1.0751	0.65872
266.	KJV55884	Virulent	0.649	Virulent	0.4926	Virulent	0.4319	Virulent	0.6424	Virulent	1.0739	0.65796
267.	KJV56581	Virulent	0.6138	Virulent	0.4435	Virulent	0.4109	Virulent	0.7369	Virulent	1.0784	0.6567
268.	KJV54971	Virulent	0.6095	Virulent	0.607	Virulent	0.3062	Virulent	0.6739	Virulent	1.0769	0.6547
269.	KJV57462	Virulent	0.7536	Virulent	0.4779	Virulent	0.3806	Virulent	0.5863	Virulent	1.0635	0.65238
270.	KJV56203	Virulent	0.6205	Virulent	0.624	Virulent	0.1794	Virulent	0.8094	Virulent	1.0262	0.6519
271.	KJV56205	Virulent	0.5801	Virulent	0.5325	Virulent	0.709	Virulent	0.3973	Virulent	1.0328	0.65034
272.	KJV51877	Virulent	0.6458	Virulent	0.5785	Virulent	0.2796	Virulent	0.6628	Virulent	1.0695	0.64724
273.	KJV56474	Virulent	0.5873	Virulent	0.5043	Virulent	0.3975	Virulent	0.6433	Virulent	1.0968	0.64584
274.	KJV55599	Virulent	0.8771	Virulent	0.4687	Virulent	0.2225	Virulent	0.6494	Virulent	1.0105	0.64564
275.	KJV57451	Virulent	0.8981	Virulent	0.5973	Virulent	0.1143	Virulent	0.7219	Virulent	0.8945	0.64522
276.	KJV55300	Virulent	0.5514	Virulent	0.4935	Virulent	0.4294	Virulent	0.6455	Virulent	1.1013	0.64422
277.	KJV57582	Virulent	0.9256	Virulent	0.6391	Virulent	0.6406	Virulent	0.0573	Virulent	0.9561	0.64374
278.	KJV57385	Virulent	0.5118	Virulent	0.3568	Virulent	1.0253	Virulent	0.2799	Virulent	1.0378	0.64232
279.	KJV57353	Virulent	0.4931	Virulent	0.6468	Virulent	0.4629	Virulent	0.5065	Virulent	1.0981	0.64148
280.	KJV57361	Virulent	0.621	Virulent	0.4511	Virulent	0.3772	Virulent	0.6598	Virulent	1.0953	0.64088
281.	KJV54535	Virulent	0.8402	Virulent	0.5042	Virulent	0.2283	Virulent	0.6061	Virulent	1.0198	0.63972
282.	KJV55284	Virulent	0.6889	Virulent	0.4981	Virulent	0.2447	Virulent	0.6969	Virulent	1.0652	0.63876
283.	KJV55744	Virulent	0.3984	Virulent	0.5909	Virulent	0.4076	Virulent	0.6427	Virulent	1.1435	0.63662

284.	KJV57412	Virulent	0.5799	Virulent	0.5011	Virulent	0.2993	Virulent	0.6746	Virulent	1.1075	0.63248
285.	KJV56780	Virulent	0.6516	Virulent	0.5008	Virulent	0.2647	Virulent	0.6524	Virulent	1.0869	0.63128
286.	KJV53192	Virulent	0.6409	Virulent	0.5601	Virulent	0.1802	Virulent	0.7096	Virulent	1.0518	0.62852
287.	KJV51128	Virulent	0.7697	Virulent	0.4255	Virulent	0.3799	Virulent	0.4846	Virulent	1.0816	0.62826
288.	KJV51649	Virulent	1.252	Virulent	0.3937	Non-Virulent	-0.072	Virulent	0.9522	Virulent	0.6021	0.6256
289.	KJV50500	Virulent	0.681	Virulent	0.4507	Virulent	0.2681	Virulent	0.6364	Virulent	1.0916	0.62556
290.	KJV54870	Virulent	0.5248	Virulent	0.5667	Virulent	0.3783	Virulent	0.6324	Virulent	1.012	0.62284
291.	KJV51829	Virulent	0.8247	Virulent	0.4773	Virulent	0.4481	Virulent	0.3128	Virulent	1.0466	0.6219
292.	KJV57315	Virulent	0.6176	Virulent	0.4837	Virulent	0.2885	Virulent	0.6117	Virulent	1.1079	0.62188
293.	KJV54388	Virulent	0.8823	Virulent	0.2322	Virulent	0.3025	Virulent	0.5868	Virulent	1.0874	0.61824
294.	KJV57362	Virulent	0.9643	Virulent	0.1822	Virulent	0.4272	Virulent	0.4281	Virulent	1.084	0.61716
295.	KJV54140	Non-Virulent	-0.065	Virulent	0.9953	Virulent	0.6844	Virulent	0.3842	Virulent	1.081	0.61598
296.	KJV52748	Virulent	0.7932	Virulent	0.4665	Virulent	0.1456	Virulent	0.6038	Virulent	1.0286	0.60754
297.	KJV57356	Virulent	0.7403	Virulent	0.2807	Virulent	0.4163	Virulent	0.5768	Virulent	1.0224	0.6073
298.	KJV55456	Virulent	0.3041	Virulent	0.4757	Virulent	0.457	Virulent	0.6418	Virulent	1.1578	0.60728
299.	KJV57432	Virulent	0.5965	Virulent	0.4861	Virulent	0.2052	Virulent	0.6403	Virulent	1.1058	0.60678
300.	KJV57365	Virulent	0.0902	Virulent	0.4597	Virulent	0.6656	Virulent	0.6316	Virulent	1.1551	0.60044
301.	KJV54530	Virulent	0.9545	Virulent	0.4733	Non-Virulent	-0.067	Virulent	0.8454	Virulent	0.741	0.58944
302.	KJV55821	Virulent	0.9927	Virulent	1.1516	Virulent	0.166	Virulent	0.1583	Virulent	0.4746	0.58864
303.	KJV56789	Virulent	0.7823	Virulent	0.4984	Non-Virulent	-0.032	Virulent	0.8234	Virulent	0.8542	0.58526
304.	KJV52622	Virulent	0.658	Virulent	0.5288	Virulent	0.1015	Virulent	0.5882	Virulent	1.041	0.5835
305.	KJV56053	Virulent	0.3017	Virulent	0.4844	Virulent	0.3442	Virulent	0.6043	Virulent	1.1744	0.5818
306.	KJV57139	Virulent	0.4591	Virulent	0.4668	Virulent	0.1531	Virulent	0.6926	Virulent	1.128	0.57992
307.	KJV56480	Virulent	0.3896	Virulent	0.4646	Virulent	0.2706	Virulent	0.5982	Virulent	1.1685	0.5783
308.	KJV55228	Virulent	0.737	Virulent	0.3057	Virulent	0.1992	Virulent	0.4967	Virulent	1.1311	0.57394
309.	KJV51000	Virulent	0.5054	Virulent	0.9542	Virulent	0.5084	Virulent	0.0186	Virulent	0.8731	0.57194
310.	KJV56209	Virulent	0.6086	Virulent	0.4334	Virulent	0.1495	Virulent	0.4967	Virulent	1.12	0.56164
311.	KJV54146	Virulent	0.5603	Virulent	0.4004	Virulent	0.1225	Virulent	0.5985	Virulent	1.1247	0.56128
312.	KJV52750	Virulent	0.3696	Virulent	0.3972	Virulent	0.5606	Virulent	0.3341	Virulent	1.1114	0.55458
313.	KJV55034	Virulent	0.3958	Virulent	0.4849	Virulent	1.0361	Non-Virulent	-0.257	Virulent	0.8992	0.5118
314.	KJV57206	Virulent	0.2624	Virulent	0.3496	Virulent	0.3877	Virulent	0.4035	Virulent	1.14	0.50864

315.	KJV54555	Virulent	0.1823	Virulent	0.1711	Virulent	0.636	Virulent	0.4384	Virulent	1.097	0.50496
316.	KJV57219	Virulent	0.1012	Virulent	0.0954	Virulent	1.1822	Non-Virulent	-0.08	Virulent	1.1175	0.48326
317.	KJV56204	Virulent	0.3022	Virulent	0.3269	Non-Virulent	-0.006	Virulent	0.755	Virulent	1.0187	0.47936
318.	KJV50940	Virulent	0.2442	Virulent	0.2884	Virulent	0.2795	Virulent	0.4459	Virulent	1.1253	0.47666
319.	KJV56047	Non-Virulent	-0.082	Virulent	0.4047	Virulent	0.4734	Virulent	0.5141	Virulent	1.0598	0.474
320.	KJV54343	Virulent	0.5956	Virulent	0.4787	Non-Virulent	-0.194	Virulent	0.6388	Virulent	0.7937	0.46256
321.	KJV57200	Virulent	0.3414	Virulent	0.3916	Virulent	0.0994	Virulent	0.3635	Virulent	1.0849	0.45616
322.	KJV56214	Virulent	1.2887	Virulent	0.4404	Non-Virulent	-0.424	Virulent	0.6419	Virulent	0.1995	0.4293
323.	KJV55028	Virulent	0.5439	Virulent	0.1359	Non-Virulent	-0.18	Virulent	0.8249	Virulent	0.8092	0.42678
324.	KJV56408	Virulent	0.1209	Virulent	0.423	Non-Virulent	-0.031	Virulent	0.6403	Virulent	0.9115	0.41294
325.	KJV54879	Virulent	0.3042	Virulent	0.357	Non-Virulent	-0.07	Virulent	0.428	Virulent	0.937	0.39124
326.	KJV55342	Virulent	0.2659	Virulent	0.2229	Non-Virulent	-0.029	Virulent	0.4175	Virulent	0.9263	0.36072
327.	KJV51252	Virulent	0.1665	Non-Virulent	-0.246	Virulent	0.8591	Virulent	0.0108	Virulent	0.9721	0.3525
328.	KJV50599	Virulent	0.3035	Virulent	0.1632	Non-Virulent	-0.015	Virulent	0.3466	Virulent	0.9173	0.34312
329.	KJV54907	Virulent	0.9064	Non-Virulent	-0.51	Virulent	0.2455	Non-Virulent	-0.034	Virulent	0.9265	0.30688
330.	KJV51581	Virulent	0.2203	Virulent	0.2395	Non-Virulent	-0.002	Virulent	0.2245	Virulent	0.7908	0.29462
331.	KJV53442	Virulent	0.2159	Virulent	0.1385	Non-Virulent	-0.006	Virulent	0.3208	Virulent	0.8027	0.29438
332.	KJV56385	Non-Virulent	-0.203	Virulent	0.0453	Virulent	0.6629	Virulent	0.0984	Virulent	0.7043	0.26158
333.	KJV54977	Virulent	0.0755	Virulent	0.1419	Virulent	0.0067	Virulent	0.3229	Virulent	0.6251	0.23442
334.	KJV51376	Virulent	0.3987	Virulent	0.1063	Virulent	0.1351	Non-Virulent	-0.206	Virulent	0.5647	0.19976
335.	KJV54464	Virulent	0.1489	Virulent	0.1342	Non-Virulent	-0.256	Virulent	0.4175	Virulent	0.409	0.17072
336.	KJV54705	Virulent	0.476	Non-Virulent	-0.334	Virulent	0.1876	Non-Virulent	-0.061	Virulent	0.5314	0.16
337.	KJV55346	Non-Virulent	-0.301	Virulent	0.2717	Virulent	0.2927	Virulent	0.2006	Virulent	0.3181	0.15642
338.	KJV54362	Virulent	0.1271	Non-Virulent	-0.095	Virulent	0.144	Non-Virulent	-0.126	Virulent	0.148	0.03962
339.	KJV51694	Non-Virulent	-0.017	Virulent	0.4981	Virulent	0.0368	Non-Virulent	-0.413	Non-Virulent	-0.181	-0.01522
340.	KJV55334	Virulent	0.4217	Non-Virulent	-0.409	Virulent	0.0829	Virulent	0.0959	Non-Virulent	-1.063	-0.1743

341.	KJV55527	Non-Virulent	-0.33	Non-Virulent	-0.286	Non-Virulent	-0.643	Non-Virulent	-0.163	Non-Virulent	-1.017	-0.4878
342.	KJV53819	Non-Virulent	-0.737	Non-Virulent	-0.39	Non-Virulent	-0.178	Non-Virulent	-0.179	Non-Virulent	-1.07	-0.5108
343.	KJV53066	Non-Virulent	-0.735	Non-Virulent	-1.189	Virulent	0.1623	Non-Virulent	-0.689	Non-Virulent	-0.808	-0.65174
344.	KJV55230	Non-Virulent	-1.127	Non-Virulent	-1.075	Non-Virulent	-0.083	Non-Virulent	-0.285	Non-Virulent	-0.956	-0.7052
