# Supplementary material for: In silico characterization of hypothetical proteins from Orientia tsutsugamushi str. Karp uncovers virulence genes
Source: Heliyon. 2019 Nov 1;5(10):e02734. doi: 10.1016/j.heliyon.2019.e02734 (PMC6838952; doi:10.1016/j.heliyon.2019.e02734)
Supplement: Supplementary file 5 [file mmc5.pdf]

S5_Table: List of proteins with its known function, Biological process and Sub cellular localization

S.No	ENTRY	Protein names	functions	Biological process	Sub cellular localization
1.	A0A0F3MMA2	Adenylate kinase	Nucleotide binding, ATP binding	nucleotide biosynthetic process	cytoplasm
2.	A0A0F3MSQ6	DNA gyrase subunit B	Metal binding, ATP binding,	DNA-dependent DNA replication	Cytoplasm
3.	A0A0F3MIU5	**DNA ligase**	Catalytic activity,DNA binding, DNA ligase (NAD+) activity	DNA repair and Replication	Cytoplasm
4.	A0A0F3MBH1	Nucleoside diphosphate kinase	Metal binding, ATP binding, nucleoside diphosphate kinase activity	biosynthetic process	Cytoplasm
5.	A0A0F3MLZ5	ATP synthase subunit alpha	ATP binding, proton-transporting ATPase activity	ATP hydrolysis coupled proton transport	Cell membrane
6.	A0A0F3MKN9	GTPase Obg	GTPase activity, Hydrolase,	ribosome biogenesis	Cytoplasm
7.	A0A0F3MS09	ATP synthase subunit beta	ATP binding, roton-transporting ATP synthase activity	ATP hydrolysis coupled proton transport	Cell membrane
8.	A0A0F3MF60	Heme A synthase	oxidoreductase activity	Heme a biosynthetic process	Cell membrane
9.	A0A0F3MN09	DNA repair protein RadA	ATP binding, damaged DNA binding, DNA-dependent ATPase activity, metal ion binding	recombinational repair	
10.	A0A0F3MCY3	DNA primase	DNA, Magnesium ion,Zin ion binding, Nucleotidyltransferase	DNA replication	primosome complex
11.	A0A0F3MPB8	NA-directed RNA polymerase subunit beta	DNA binding, DNA-directed RNA polymerase activity, ribonucleoside binding,	ranscription, DNA-templated	DNA-directed RNA polymerase
12.	A0A0F3MRH7	Cell division protein FtsZ	GTPase activity, GTP binding	barrier septum assembly,	Cytoplasm, cell division site
13.	A0A0F3MPE5	Protein RecA	ATP binding, damaged DNA binding, DNA-dependent ATPase activity,	DNA recombination, DNA repair, SOS response	Cytoplasm
14.	A0A0F3MG82	Guanylate kinase	guanylate kinase activity, ATP binding	**Kinase**	Cytoplasm

15.	A0A0F3MLZ2	Cytochrome b	electron carrier activity, ubiquinol-cytochrome-c reductase activity	mitochondrial electron transport, ubiquinol to cytochrome c	mitochondrion
16.	A0A0F3ML94	Replicative DNA helicase	ATP binding, DNA binding, DNA helicase activity	DNA replication, synthesis of RNA primer	primosome complex
17.	A0A0F3MNH4	Ferrochelatase	ferrochelatase activity, metal ion binding	heme biosynthetic process	Cytoplasm
18.	A0A0F3MK73	Thymidylate kinase	ATP binding, Thymidylate kinase activity	dTDP biosynthetic process	
19.	A0A0F3MLZ2	Cytochrome b	electron carrier activity, ubiquinol-cytochrome-c reductase activity	mitochondrial electron transport	mitochondrion
20.	A0A0F3MIE4	Protein GrpE	adenyl-nucleotide exchange factor activity	protein folding	Cytoplasm
21.	A0A0F3MNH3	Aspartokinase	aspartate kinase activity	lysine biosynthetic process via diaminopimelate	
22.	A0A0F3MQ17	RNA pyrophosphohydrolase	in phosphorus-containing anhydrides, metal ion binding	hydrolase activity	
23.	A0A0F3MHE4	Elongation factor G	GTPase activity, ranslation elongation factor activity	Protein biosythesis	Cytoplasm
24.	A0A0F3MN93	Hemolysin C	flavin adenine dinucleotide binding, oxidoreductase activity		
25.	A0A0F3MMB8	Methionyl-tRNA formyltransferase	methionyl-tRNA formyltransferase activity	Protein biosynthesis	
26.	A0A0F3MPE6	Thioredoxin	protein disulfide oxidoreductase activity	cell redox homeostasis, glycerol ether metabolic process	cell
27.	A0A0F3M6P2	DNA helicase	ATP binding, ATP-dependent DNA helicase activity, DNA binding	Hydrolase	Cytoplasm
28.	A0A0F3MFK0	Aminotransferase	pyridoxal phosphate binding, transaminase activity	biosynthetic process	Cytoplasm
29.	A0A0F3MK82	DNA polymerase I	DNA binding, DNA-directed DNA polymerase activity, nuclease activity	DNA-dependent DNA replication	
30.	A0A0F3MPF0	RIP metalloprotease RseP	metal ion binding, metalloendopeptidase activity,		integral component of

			Metalloprotease		membrane
31.	A0A0F3MIV6	Metallopeptidase M24 family protein	metalloaminopeptidase activity		
32.	A0A0F3MKT7	Cell division protein FtsA	FtsZ-dependent cytokinesis	Cell cycle, Cell division	cell division site, cytoplasmic side of plasma membrane
33.	A0A0F3MJ99	tRNA-dihydrouridine synthase	flavin adenine dinucleotide binding	tRNA dihydrouridine synthase activity	
34.	A0A0F3MF86	ajor Facilitator Superfamily protein	transmembrane transport	Transportation	integral component of membrane
35.	A0A0F3MSQ2	Amino acid permease family protein	mino acid transmembrane transporter activity	transmembrane transport	Membrane
36.	A0A0F3MPD3	Na+/H+ ion antiporter subunit	cation transmembrane transporter activity	transmembrane transport	integral component of membrane
37.	A0A0F3MJ14	Mce related family protein	phospholipid transport		integral component of membrane
38.	A0A0F3MFD0	TraE family protein	conjugation		integral component of membrane
39.	A0A0F3MFR6	Type IV secretion-system coupling DNA-binding domain protein	DNA binding	DNA replication, synthesis of RNA primer	ntegral component of membrane
40.	A0A0F3MQ09	ATP synthase family protein	proton-transporting ATP synthase activity, rotational mechanism	ATP synthesis coupled proton transport	proton-transporting ATP synthase complex, catalytic core F
41.	B3CSR0	D-alanine--D-alanine ligase	ATP binding, D-alanine-D-alanine ligase activity, magnesium ion binding, manganese ion binding	cell wall organization,peptidoglycan biosynthetic	Cytoplasm

				process,regulation of cell shape	
42.	A0A0F3MLW3	ATP-dependent zinc metalloprotease FtsH	ATPase activity , ATP binding, metalloendopeptidase activity, zinc ion binding	protein catabolic process	Cell membrane
43.	A5CDC6	ATP synthase subunit c	hydrogen ion transmembrane transporter activity, lipid binding	ATP hydrolysis coupled proton transport, ATP synthesis coupled proton transport	Cell inner membrane
44.	A5CD80	Uridylate kinase	ATP binding, UMP kinase activity	'de novo' CTP biosynthetic process	Cytoplasm
45.	B3CVL2	Ribonuclease 3	metal ion binding,ribonuclease III activity, rRNA binding	tRNA,rRNA,mRNA processing, rRNA catabolic process	Cytoplasm
46.	A0A0F3MHW1	Zinc-binding dehydrogenase family protein	oxidoreductase activity, zinc ion binding		
47.	A0A0F3MIY6	Aconitase family protein	4 iron, 4 sulfur cluster binding	metabolic process	
48.	A0A0F3MKJ2	Hormone receptor domain protein	G-protein coupled receptor activity		membrane
49.	A0A0F3M9E3	tRNA-specific 2-thiouridylase MnmA	ATP binding, methyltransferase activity, sulfurtransferase activity, tRNA binding	tRNA modification	Cytoplasm
50.	A0A0F3PBA1	Serine hydroxymethyltransferase	glycine hydroxymethyltransferase activity, methyltransferase activity, pyridoxal phosphate binding	glycine biosynthetic process from serine, tetrahydrofolate interconversion	Cytoplasm
51.	A0A0F3RKM4	Phosphatidylserine decarboxylase proenzyme	phosphatidylserine decarboxylase activity,	phosphatidylethanolamine biosynthetic process	Cell membrane
52.	A0A0F3MDM5	DNA gyrase subunit B	ATP binding, DNA binding, DNA topoisomerase type II (ATP-hydrolyzing) activity, DNA topoisomerase type II (ATP-hydrolyzing) activity	DNA-dependent DNA replication, DNA topological change	Cytoplasm
53.	B3CUJ4	ATP-dependent zinc metalloprotease FtsH	ATPase activity , ATP binding, metalloendopeptidase activity, zinc ion	protein catabolic process	Cell inner membrane, Multi-

			binding		pass membrane protein , Cytoplasmic side
54.	A5CDU5	Serine--tRNA ligase	ATP binding, serine-tRNA ligase activity	selenocysteine biosynthetic process, seryl-tRNA aminoacylation, selenocysteinyl-tRNA(Sec) biosynthetic process	cytoplasm
55.	A5CF58	Bifunctional protein FolD	methenyltetrahydrofolate cyclohydrolase activity, methylenetetrahydrofolate dehydrogenase (NADP+) activity	folic acid-containing compound biosynthetic process,<br><br>histidine biosynthetic process,<br><br>methionine biosynthetic process ,<br><br>purine nucleotide biosynthetic process,<br><br>tetrahydrofolate interconversion	
56.	B3CUN9	Lon protease	ATP binding,ATP-dependent peptidase activity,sequence-specific DNA binding, serine-type endopeptidase activity	cellular response to stress, misfolded or incompletely synthesized protein catabolic process	cytoplasm
57.	A5CD21	UDP-N-acetylenolpyruvoylglucosamine reductase	flavin adenine dinucleotide binding, UDP-N-acetylmuramate dehydrogenase activity	cell cycle,cell division, cell wall organization, peptidoglycan biosynthetic process,regulation of cell shape	cytoplasm
58.	B3CSR0	D-alanine--D-alanine ligase	ATP binding, D-alanine-D-alanine ligase	cell wall organization,	cytoplasm

			activity, mg and mn ion binding	peptidoglycan biosynthetic process, regulation of cell shape	
59.	A0A0F3MS85	ATP-dependent zinc metalloprotease FtsH	ATPase activity , ATP binding, metalloendopeptidase activity, zinc ion binding	protein catabolic process	Cell membrane
60.	B3CQT8	ATP synthase subunit c	hydrogen ion transmembrane transporter activity, lipid binding	ATP hydrolysis coupled proton transport, ATP synthesis coupled proton transport, ATP synthesis, Hydrogen ion transport, Ion transport, Transport	Cell inner membrane
